# Morbidity and mortality due to shigella and enterotoxigenic *Escherichia coli* diarrhoea: the Global Burden of Disease Study 1990–2016

**DOI:** 10.1016/S1473-3099(18)30475-4

**Published:** 2018-11

**Authors:** Ibrahim A Khalil, Christopher Troeger, Brigette F Blacker, Puja C Rao, Alexandria Brown, Deborah E Atherly, Thomas G Brewer, Cyril M Engmann, Eric R Houpt, Gagandeep Kang, Karen L Kotloff, Myron M Levine, Stephen P Luby, Calman A MacLennan, William K Pan, Patricia B Pavlinac, James A Platts-Mills, Firdausi Qadri, Mark S Riddle, Edward T Ryan, David A Shoultz, A Duncan Steele, Judd L Walson, John W Sanders, Ali H Mokdad, Christopher J L Murray, Simon I Hay, Robert C Reiner

**Affiliations:** a Institute for Health Metrics and Evaluation, Seattle WA, USA; b Center for Vaccine Innovation and Access, PATH, Seattle, WA, USA; c Maternal, Newborn, Child Health & Nutrition, PATH, Seattle, WA, USA; d Drug Development, PATH, Seattle, WA, USA; e Department of Global Health, University of Washington School of Public Health, Seattle, WA, USA; f Department of Epidemiology, University of Washington School of Public Health, Seattle, WA, USA; g Department of Pediatrics, University of Washington School of Medicine, Seattle, WA, USA; h Department of Medicine, University of Washington School of Medicine, Seattle, WA, USA; i Department of Epidemiology, University of Washington School of Medicine, Seattle, WA, USA; j Division of Infectious Diseases and International Health, University of Virginia, Charlottesville, VA, USA; k Translational Health Science and Technology Institute, Faridabad, India; l Department of Pediatrics, University of Maryland School of Medicine, Baltimore, MD, USA; m Department of Medicine, University of Maryland School of Medicine, Baltimore, MD, USA; n Center for Vaccine Development and Global Health, University of Maryland School of Medicine, Baltimore, MD, USA; o Division of Infectious Diseases and Geographic Medicine, Stanford University, Stanford, CA, USA; p Enteric and Diarrheal Diseases, Bill & Melinda Gates Foundation, Seattle, WA, USA; q Duke Global Health Institute, Duke University, Durham, NC, USA; r Infectious Diseases Division, International Center for Diarrhoeal Disease Research, Bangladesh, Dhaka, Bangladesh; s Uniformed Services University, Bethesda, MD, USA; t Division of Infectious Diseases, Department of Medicine, Massachusetts General Hospital, Boston, MA, USA; u Department of Medicine, Harvard Medical School, Boston, MA, USA; v Department of Immunology & Infectious Diseases, Harvard School of Public Health, Boston, MA, USA; w Albers School of Business & Economics, Seattle University, Seattle, WA, USA; x Wake Forest University School of Medicine, Winston-Salem, NC, USA; y Big Data Institute, Li Ka Shing Centre for Health Information and Discovery, University of Oxford, Oxford, UK

## Abstract

**Background:**

Shigella and enterotoxigenic *Escherichia coli* (ETEC) are bacterial pathogens that are frequently associated with diarrhoeal disease, and are a significant cause of mortality and morbidity worldwide. The Global Burden of Diseases, Injuries, and Risk Factors study 2016 (GBD 2016) is a systematic, scientific effort to quantify the morbidity and mortality due to over 300 causes of death and disability. We aimed to analyse the global burden of shigella and ETEC diarrhoea according to age, sex, geography, and year from 1990 to 2016.

**Methods:**

We modelled shigella and ETEC-related mortality using a Bayesian hierarchical modelling platform that evaluates a wide range of covariates and model types on the basis of vital registration and verbal autopsy data. We used a compartmental meta-regression tool to model the incidence of shigella and ETEC, which enforces an association between incidence, prevalence, and remission on the basis of scientific literature, population representative surveys, and health-care data. We calculated 95% uncertainty intervals (UIs) for the point estimates.

**Findings:**

Shigella was the second leading cause of diarrhoeal mortality in 2016 among all ages, accounting for 212 438 deaths (95% UI 136 979–326 913) and about 13·2% (9·2–17·4) of all diarrhoea deaths. Shigella was responsible for 63 713 deaths (41 191–93 611) among children younger than 5 years and was frequently associated with diarrhoea across all adult age groups, increasing in elderly people, with broad geographical distribution. ETEC was the eighth leading cause of diarrhoea mortality in 2016 among all age groups, accounting for 51 186 deaths (26 757–83 064) and about 3·2% (1·8–4·7) of diarrhoea deaths. ETEC was responsible for about 4·2% (2·2–6·8) of diarrhoea deaths in children younger than 5 years.

**Interpretation:**

The health burden of bacterial diarrhoeal pathogens is difficult to estimate. Despite existing prevention and treatment options, they remain a major cause of morbidity and mortality globally. Additional emphasis by public health officials is needed on a reduction in disease due to shigella and ETEC to reduce disease burden.

**Funding:**

Bill & Melinda Gates Foundation.

## Introduction

According to recent global disease burden estimates, diarrhoea accounts for more than 1 million deaths and about 4% of the total global disability-adjusted life-years (DALYS) per year across all age groups.^1^, ^2^, ^3^ We have previously reported the number of diarrhoea deaths attributable to shigella (212 400 deaths, 95% uncertainty interval [UI] 137 000–326 900) and to enterotoxigenic *Escherichia coli* (ETEC; 51 186 deaths, 26 757–83 064).^1^ Here, we will extend those results by focusing on the burden of shigella and ETEC. Although mortality rates from diarrhoeal diseases have decreased since 1990, diarrhoea morbidity remains high, particularly in low-income and middle-income countries (LMICs), where access to care, relevant microbiological diagnostics, water quality, and sanitation are poor, and adequate health-care facilities, diagnostics, and treatment interventions are scarce.^1^, ^4^ Shigella and ETEC are among the leading causes of diarrhoea in children and adults in LMICs, and among travellers and military personnel from high-income countries.^5^, ^6^, ^7^, ^8^, ^9^, ^10^, ^11^ The detection of bacterial pathogens, especially shigella, through conventional approaches was, in the past, restricted by inconsistent diagnostic accuracy and inaccurate surveillance methods. The use of real-time PCR diagnostics has substantially improved the detection of shigella and ETEC pathogens and, therefore, has increased the fraction of moderate and severe diarrhoea cases that are attributable to these pathogens.^12^, ^13^

ETEC is one of the first symptomatic enteric illnesses for many children, causing several hundred million cases of diarrhoea each year, mostly in young children.^14^, ^15^ Repeated ETEC infections are common among children in low-income countries because of the multiple pathotypes (enterotoxin and colonisation factor combinations) associated with the disease; however, the decrease in the incidence of symptomatic illness with increasing age shows that protective immunity develops,^14^, ^16^, ^17^, ^18^ and the incidence of ETEC diarrhoea in low-income countries peaks in the first 2 years of life. ETEC is the most common cause of diarrhoea in travellers, affecting individuals from high-income countries who visit endemic areas in LMICs.^19^ A systematic review suggests that ETEC was detected in 30·4% of cases of diarrhoea in travellers, with the highest rates seen in those travelling to areas with a high prevalence of ETEC.^11^

**Research in context**

**Evidence before this study**

Sources for this analysis of the global burden of shigella and enterotoxigenic *Escherichia coli* (ETEC) diarrhoea include population representative surveys, scientific literature, and health-care utilisation data. We searched PubMed, with no language restrictions, for studies published between Jan 1, 1990, and Dec 31, 2017, with the following search string: (diarrhoea [title] OR diarrhoea [MeSH terms] OR diarrhoea [title] OR diarrhoea [MeSH terms] AND (shigell* [title/abstract] OR enterotoxigenic e. coli [title/abstract]) AND (aetiolog* [title/abstract] OR aetiology [MeSH Terms] OR cause [title/abstract] OR pathogen [title/abstract]) NOT (colitis [title/abstract] OR enterocolitis [title/abstract] OR inflammatory bowel [title/abstract] OR irritable [title/abstract] OR Crohn* [title/abstract] OR HIV [title] OR treatment [title] OR therapy [title]) NOT (appendicitis [title/abstract] OR esophag* [title/abstract] OR surger* [title/abstract] OR gastritis [title/abstract] OR liver [title/abstract] OR case report [title] OR case-report [title] OR therapy [title] OR treatment [title]) AND humans [Mesh]). The Maternal and Child Epidemiology Estimation group (MCEE) estimated 42 000 deaths among children younger than 5 years due to ETEC and 28 000 deaths due to shigella. The MCEE modelling approach was categorical, meaning that if a pathogen was present in a diarrhoeal stool sample, diarrhoea was attributed to that pathogen, and used conventional bacterial culture methods for diagnostic detection. The Global Burden of Diseases, Injuries, and Risk Factors (GBD) study 2016 used molecular diagnostics.

**Added value of this study**

Our analysis uses the GBD study to estimate shigella and ETEC incidence, disability-adjusted life-years, and mortality across every country for each sex and all ages from 1990 to 2016. We estimated that shigella was responsible for about 210 000 deaths among all ages, including about 63 700 among children younger than 5 years, and that ETEC was responsible for about 51 200 deaths among all ages and about 18 700 deaths in children younger than 5 years. Our results challenge some previous estimates with regard to the relative and absolute magnitude of the health burden associated with diarrhoea caused by shigella and ETEC.

**Implications of all the available evidence**

Our study calls for a widespread improvement in the quality and quantity of data, including improved surveillance systems and utilisation of standard reporting mechanisms and case definitions. Refined burden estimates for the acute and long-term burden of shigella and ETEC are needed to guide funders and public health officials to make evidence-based decisions for the alleviation of diarrhoeal diseases, with particular attention to the development of effective and attainable vaccines. Data on the burden of diarrhoeal diseases caused by shigella and ETEC will help public health officials to identify proper age appropriate vaccination schedules and target regions where the burden of these pathogens is substantial.

Although shigellosis occurs worldwide, the greatest burden is among children in low-income countries. Repeated infection is not uncommon because of the multiple serotypes associated with illness, and the decrease in the incidence of disease with increasing age shows that immunity eventually develops.^20^, ^21^ Shigella is also a major cause of illness among travellers, deployed military personnel, and expatriates in LMICs, and is associated with persistent diarrhoea (≥14 days) in these populations.^22^, ^23^ Among travellers, shigella and ETEC are associated with chronic functional bowel disorders among 10–15% of individuals after acute episodes of disease. Both pathogens can be associated with irritable bowel syndrome and shigella can also trigger reactive arthritis.^24^

Both shigella and ETEC are important causes of diarrhoea and dysentery in people older than 5 years, with an estimated 100 million episodes occurring annually among those aged 5–14 years.^1^, ^9^ Both agents also have epidemic potential in both younger and older age groups.^25^, ^26^ Repeated infections and symptomatic episodes due to these pathogens can also induce or exacerbate stunting and other forms of malnutrition, reduce immune function, and increase the propensity for subsequent chronic inflammatory bowel disease.^27^, ^28^, ^29^, ^30^ These infections can also hinder cognitive development, with adverse consequences on school performance and economic status.^29^, ^31^, ^32^, ^33^ Oral rehydration salts and, when appropriate and available, antimicrobials are used as treatment.^34^ The rise of antibiotic-resistant enteric bacteria,^35^, ^36^ particularly shigella, has made the prevention of infectious diarrhoea, and the need for an effective vaccine, an even greater public health priority.^37^, ^38^

Shigella and ETEC vaccine candidates are currently in various phases of research and development.^14^, ^21^, ^38^, ^39^ ETEC and *Shigella* spp are antigenically diverse, encompassing two toxins and over 25 colonisation factors for ETEC, and 50 serotypes or subtypes for shigella, which makes the development of vaccines challenging.^16^, ^40^ Efforts to develop vaccines for ETEC have focused on inducing antitoxin and anti colonisation antigen immunity, because studies show that antibodies against both antigen types can contribute to protection and thus have potential for vaccines. The most common colonisation factors associated with ETEC diarrhoea are CFA/I, CS3, and CS6. The basis of most shigella vaccines is the O-polysaccharide, which confers protective immunity that is specific to serotype and subserotype.^41^, ^42^, ^43^, ^44^ The most common shigella serotypes are *Shigella flexneri* 2a, 6, 3a, and *Shigella sonnei.*^20^, ^40^ Live attenuated, killed whole cell, and subunit vaccines containing various combinations and presentations of these antigens are in clinical development alone or as combined vaccines against shigella and ETEC.^38^, ^45^

To inform vaccine development priorities, the disease burdens of shigella and ETEC need to be characterised at regional and national levels. Co-infecting pathogens, asymptomatic infections, antigenic diversity, and variability of diagnostic methods can complicate the determination of diarrhoeal aetiology for children in LMICs.^6^ Analyses with sensitive real-time PCR detection methods in seven LMICs have shown that the global disease burden for shigella is worse than previously estimated.^13^ Here, we describe the global burden of shigella and ETEC incidence and mortality and demonstrate the need for additional strategies to prevent infection from these bacterial pathogens, which might include separate or combination vaccines and other suitable interventions—such as access to safe water, improved sanitation, and enhanced food hygiene.

## Methods

### Overview

Detailed methods on the Global Burden of Disease (GBD) Study and on diarrhoea estimation in GBD have already been published.^1^, ^4^ We describe these methods briefly, focusing on aetiological attribution and changes from previous GBD methods.

### Estimation of diarrhoea-related mortality

Diarrhoea-related mortality was modelled in the Cause of Death Ensemble model (CODEm) platform.^2^, ^46^ CODEm is a Bayesian, hierarchical, space-time, ensemble modelling tool. CODEm produces various submodels that include a diverse set of covariates and model types, including spatiotemporal Gaussian process regression and mixed-effects models. Each submodel is weighted on the basis of out-of-sample predictive validity and contributes draws to a final set of 1000 draws. These predictive regression models produce estimates of cause-specific mortality for each age, sex, geography, and year on the basis of vital registration, verbal autopsy, and surveillance system data.

### Estimation of diarrhoea-related morbidity

Diarrhoea-related morbidity, including incidence and prevalence, was modelled in DisMod-MR (version 2.1).^47^ DisMod is a Bayesian, hierarchical meta-regression tool. Like CODEm, DisMod uses space-time information and covariates to produce modelled estimates for each age, year, geography, and sex. DisMod also contains a compartmental model where incidence, prevalence, and mortality are related in a series of ordinary differential equations. Data for these models are input from the scientific literature, surveys that are representative of the population, and hospital and health-care utilisation records.

### Estimation of diarrhoeal aetiology

The cause of diarrhoea is estimated separately from mortality and morbidity.^1^, ^4^ Most diarrhoeal aetiologies, including shigella and ETEC, are attributed via a counterfactual approach called population attributable fraction (PAF). Our approach accounted for pathogen codetection, detection in healthy individuals, and does not necessitate a one pathogen to one episode relationship. The population attributable fraction is defined as:

$$\text{PAF}=\text{proportion}\times \left(1-\frac{1}{\text{odds ratio}}\right)$$

where the odds ratio (OR) is the odds of diarrhoea given pathogen detection and the proportion is the modelled frequency of detection of the pathogen in diarrhoea samples. The ORs are based on results from the Global Enteric Multicenter Study (GEMS),^6^, ^13^ which captures moderate and severe diarrhoeal episodes. By contrast to previous rounds of GBD that followed the GEMS age groups, for GBD 2016, we defined ORs for children younger than 1 year and for all age groups older than 1 year. Because of an absence of ORs in older children and adults, we used the ORs of children aged older than 1 year in GEMS for all GBD age groups older than 1 year. The proportion estimates are from DisMod models where the input data are from scientific literature and modelled for each age, sex, year, and geography. Data extracted from the scientific literature were inclusive of all *Shigella* spp and for both heat stable (ST)-ETEC and heat labile (LT)-ETEC.

### Determination of a molecular case definition

Diarrhoea aetiologies are based on molecular diagnostic case definitions. We did a systematic reanalysis of the GEMS samples using real-time PCR. Our modelling strategy requires that the continuous real-time PCR test results be dichotomised into positive and negative results. To do this, we identified the lowest cycle threshold at which the diagnostic accuracy, defined as the ability to discriminate between cases and controls, was maximised. We fitted a Loess curve to each cycle threshold distribution of aetiology and the proportion of diarrhoea cases that were correctly identified (appendix p 5).

Because most of the scientific literature did not use molecular diagnostics, we adjusted our model estimates from the culture diagnostic-based results to our molecular-based case definition by estimating the diagnostic sensitivity and specificity of the culture diagnostic results to the real-time PCR results in the GEMS samples. We defined an ETEC-positive stool sample as one with either *estA* or *eltB E coli* genes in the primary GEMS laboratory results and the lower cycle threshold score for ST (both *STh* and *STp* genes) or LT gene targets in the real-time PCR reanalysis (appendix p 6). Therefore, our results are combined for ST-ETEC and LT-ETEC.

To attribute diarrhoea episodes and deaths to shigella and ETEC, we multiplied the PAF estimates by the diarrhoea episode and total diarrhoea deaths. All estimates in GBD are produced at the draw level with uncertainty carried through each step of the process. We present mean values from these 1000 draws with uncertainty represented by the 2·5 and 97·5 percentiles of the distributions.

### Role of the funding source

The sponsor of the study had no role in study design, data collection, data analysis, data interpretation, or writing of the report. The corresponding author had full access to all the data in the study and had final responsibility for the decision to submit for publication.

## Results

**Figure 1:**
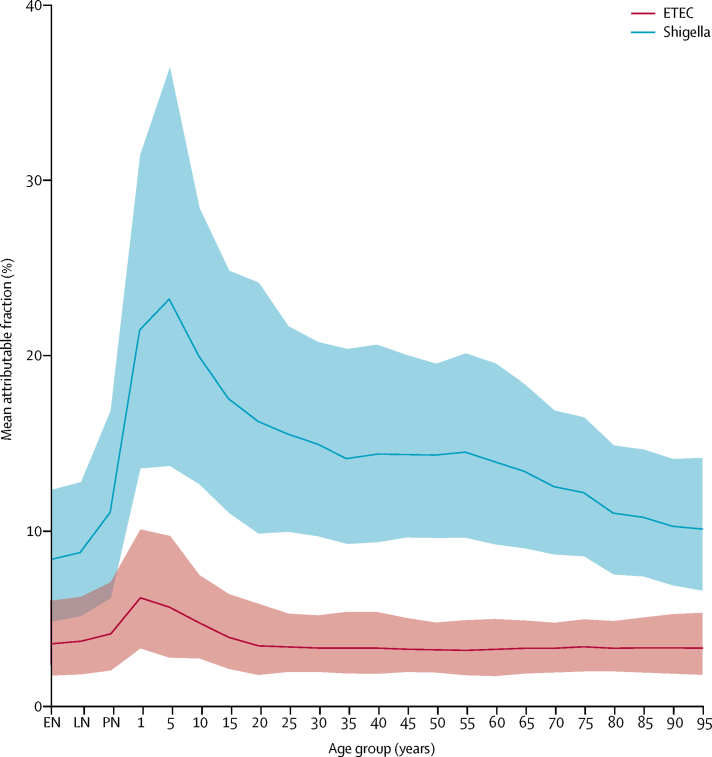
The age distribution of the population attributable fraction of diarrhoea mortality at the global level in 2016 for shigella and ETEC The population attributable fraction represents the proportion of diarrhoea deaths that are due to each pathogen. Ribbons are 95% uncertainty intervals around the mean estimates. ETEC=enterotoxigenic *Escherichia coli*. EN=early neonatal. LN=late neonatal. PN=postnatal.

As previously reported,^1^ shigella was responsible for an estimated 212 438 deaths (95% UI 136 979–326 913; table) globally among all ages in 2016, which accounts for roughly 13·2% of all diarrhoea deaths (9·2–17·4; figure 1). Shigella was the second leading cause of diarrhoea mortality in 2016 among all ages. Among children younger than 5 years, shigella was responsible for an estimated 63 713 deaths (41 191–93 611; table),^1^ representing a slightly higher PAF in this age group than in all age groups (14·0%, 9·2–20·1; figure 1). Among all ages, although mortality did not differ between men and women, the mean estimated mortality rate was slightly higher among women (3·2 per 100 000 women) than among men (2·6 per 100 000 men). The diarrhoea mortality rate attributable to shigella decreased by 55·5% (52·3–56·9) between 1990 and 2016, from 6·45 deaths per 100 000 (4·29–9·27) in 1990 to 2·87 deaths per 100 000 (1·85–4·42) in 2016. The greatest number of deaths due to shigella among all ages was in south Asia (table).

**Table:** Mortality for enterotoxigenic *Escherichia coli* and shigella in children aged younger than 5 years and people of all ages in 2016 by Global Burden of Diseases region

	**Younger than 5 years**				**All ages**			
	Deaths	Deaths per 100 000	Incidence per 1000	Cases	Deaths	Deaths per 100 000	Incidence per 1000	Cases
**Shigella**								
Global	63 713 (41 191–93 611)	10·1 (6·5–14·8)	116·2 (64·3–198·6)	74 771 591 (41 395 286–127 742 524)	212 438 (136 979–326 913)	2·9 (1·9–4·4)	36·4 (23·9–49·8)	269 191 131 (176 677 465–368 995 635)
High-income North America	13 (7–20)	0·1 (0·0–0·1)	7·0 (2·4–13·9)	149 441 (52 237–299 100)	666 (444–902)	0·2 (0·1–0·3)	3·6 (2·2–5·1)	1 284 736 (804 562–1 820 497)
Australasia	0 (0–1)	0·0 (0·0–0·0)	1·7 (0·5–3·9)	3168 (870–7035)	14 (8–20)	0·0 (0·0–0·1)	1·3 (0·7–2·0)	36 416 (18 800–57 873)
High-income Asia-Pacific	0 (0–1)	0·0 (0·0–0·0)	0·2 (0·1–1·1)	1778 (717–8373)	35 (6–74)	0·0 (0·0–0·0)	0·1 (0·0–0·2)	11 608 (5854–34 659)
Western Europe	3 (1–5)	0·0 (0·0–0·0)	4·4 (0·7–14·2)	97 088 (14 470–315 796)	253 (74–468)	0·1 (0·0–0·1)	0·7 (0·2–1·7)	305 157 (85 895–732 647)
Southern Latin America	18 (11–26)	0·4 (0·2–0·5)	102·0 (51·7–172·5)	510 676 (258 860–863 584)	160 (111–212)	0·2 (0·2–0·3)	18·9 (12·2–26·8)	1 236 711 (793 600–1 748 503)
Eastern Europe	5 (3–9)	0·0 (0·0–0·1)	18·0 (3·1–42·5)	246 225 (42 744–582 156)	16 (9–24)	0·0 (0·0–0·0)	3·0 (0·7–6·3)	637 552 (158 281–1 326 809)
Central Europe	2 (1–3)	0·0 (0·0–0·1)	24·7 (3·3–59·9)	141 247 (19 115–341 975)	21 (12–32)	0·0 (0·0–0·0)	3·2 (0·7–6·7)	371 186 (85 778–780 121)
Central Asia	51 (24–87)	0·5 (0·3–0·9)	11·7 (1·9–28·5)	128 492 (21 248–314 213)	62 (32–104)	0·1 (0·0–0·1)	3·4 (0·9–7·2)	307 312 (76 571–645 813)
Central Latin America	543 (339–818)	2·4 (1·5–3·6)	149·7 (79·0–272·8)	3 345 795 (1 766 692–6 097 394)	1448 (1033–1948)	0·6 (0·4–0·8)	39·0 (24·1–57·0)	9 911 117 (6 129 519–14 485 425)
Andean Latin America	49 (29–77)	0·7 (0·4–1·2)	69·6 (33·7–121·4)	456 738 (221 153–796 428)	123 (77–206)	0·2 (0·1–0·3)	23·5 (13·8–34·9)	1 403 049 (827 826–2 085 319)
Caribbean	85 (34–175)	2·1 (0·8–4·4)	14·7 (1·7–44·3)	60 519 (7022–182 851)	152 (72–266)	0·3 (0·2–0·6)	3·7 (1·0–8·8)	169 689 (46 754–401 393)
Tropical Latin America	182 (117–265)	1·1 (0·7–1·6)	311·6 (174·3–489·1)	4 443 578 (2 485 666–6 973 325)	683 (484–885)	0·3 (0·2–0·4)	57·4 (37·1–79·1)	12 324 291 (7 970 931–16 976 528)
East Asia	133 (79–211)	0·2 (0·1–0·3)	22·6 (11·4–40·4)	1 493 428 (755 242–2 669 927)	391 (238–677)	0·0 (0·0–0·0)	5·2 (3·2–7·6)	7 376 462 (4 478 357–10 810 483)
Southeast Asia	2427 (1503–3719)	4·3 (2·6–6·5)	149·3 (78·9–269·6)	8 775 161 (4 639 168–15 844 104)	13 337 (7670–21 358)	2·0 (1·2–3·3)	56·5 (36·5–77·7)	37 127 957 (23 990 739–51 027 995)
Oceania	141 (67–265)	10·0 (4·8–18·7)	345·3 (199·9–564·1)	476 835 (275 998–778 874)	743 (419–1259)	6·6 (3·7–11·2)	190·9 (128·1–252·9)	2 133 859 (1 431 886–2 826 367)
North Africa and Middle East	1823 (952–3075)	2·9 (1·5–4·9)	107·3 (48·3–208·1)	6 814 981 (3 068 712–13 219 732)	2744 (1566–4259)	0·5 (0·3–0·7)	40·4 (24·3–60·9)	23 221 621 (13 967 835–35 010 094)
South Asia	10 443 (6658–15 566)	6·8 (4·3–10·1)	89·5 (51·1–143·0)	14 308 810 (8 166 722–22 853 542)	78 392 (47 670–134 099)	4·6 (2·8–7·9)	43·2 (28·9–57·2)	73 683 839 (49 272 837–97 539 370)
Southern sub-Saharan Africa	1741 (1117–2652)	20·2 (13·0–30·8)	189·4 (109·5–301·6)	1 716 531 (992 173–2 733 071)	4726 (2946–7279)	6·1 (3·8–9·5)	107·9 (73·3–139·2)	8 351 367 (5 673 379–10 780 978)
Western sub-Saharan Africa	29 027 (17 665–45 045)	44·9 (27·3–69·7)	210·6 (117·0–355·1)	13 751 383 (7 637 743–23 188 393)	45 813 (28 828–68 003)	11·5 (7·2–17·1)	82·5 (53·2–116·0)	32 898 776 (21 218 837–46 292 357)
Eastern sub-Saharan Africa	14 934 (9448–22 501)	23·9 (15·1–36·0)	259·0 (142·8–448·5)	16 154 581 (8 907 765–27 978 045)	57 473 (36 018–90 963)	14·8 (9·3–23·5)	133·1 (87·0–181·5)	51 479 886 (33 640 524–70 237 981)
Central sub-Saharan Africa	2094 (950–3702)	10·1 (4·6–17·8)	97·1 (33·6–205·2)	2 062 550 (714 274–4 357 213)	5186 (2966–8346)	4·4 (2·5–7·1)	39·4 (19·5–69·7)	4 660 748 (2 307 383–8 243 199)
**Enterotoxigenic *Escherichia coli***								
Global	18 669 (9800–30 659)	3·0 (1·6–4·9)	116·8 (61·7–202·6)	75 163 376 (39 689 144–130 352 142)	51 186 (26 757–83 064)	0·7 (0·4–1·1)	30·1 (19·6–43·6)	222 637 561 (144 947 450–322 845 099)
High-income North America	0 (0–0)	0·0 (0·0–0·0)	0·2 (0·1–0·3)	4301 (2817–6078)	6 (4–7)	0·0 (0·0–0·0)	0·1 (0·0–0·1)	24 644 (17 158–31 715)
Australasia	0 (0–0)	0·0 (0·0–0·0)	0·2 (0·0–1·0)	313 (71–1813)	0 (0–0)	0·0 (0·0–0·0)	0·1 (0·0–0·1)	1532 (791–4153)
High-income Asia-Pacific	0 (0–0)	0·0 (0·0–0·0)	0·3 (0·1–1·6)	2157 (463–12 190)	2 (1–3)	0·0 (0·0–0·0)	0·0 (0·0–0·1)	7655 (3726–22 502)
Western Europe	3 (1–6)	0·0 (0·0–0·0)	20·9 (5·9–48·6)	464 291 (131 365–1 079 493)	174 (41–388)	0·0 (0·0–0·1)	3·5 (1·9–5·7)	1 487 136 (797 182–2 439 141)
Southern Latin America	0 (0–0)	0·0 (0·0–0·0)	1·7 (0·4–10·3)	8722 (1872–51 414)	1 (0–1)	0·0 (0·0–0·0)	0·2 (0·1–0·9)	15 869 (5518–61 028)
Eastern Europe	8 (4–13)	0·1 (0·0–0·1)	123·9 (71·3–204·2)	1 695 395 (976 167–2 794 484)	23 (14–33)	0·0 (0·0–0·0)	23·1 (15·4–32·7)	4 907 596 (3 267 953–6 934 008)
Central Europe	4 (2–8)	0·1 (0·0–0·1)	213·7 (90·2–416·3)	1 220 079 (514 888–2 376 811)	40 (22–64)	0·0 (0·0–0·1)	29·7 (18·0–46·7)	3 441 408 (2 088 315–5 412 845)
Central Asia	74 (37–127)	0·8 (0·4–1·3)	82·6 (45·8–138·7)	909 028 (504 335–1 527 024)	89 (48–144)	0·1 (0·1–0·2)	25·5 (16·6–37·1)	2 286 663 (1 489 518–3 330 103)
Central Latin America	150 (77–242)	0·7 (0·3–1·1)	111·4 (54·9–205·4)	2 490 169 (1 227 153–4 590 038)	462 (276–677)	0·2 (0·1–0·3)	29·8 (18·6–45·7)	7 578 241 (4 715 595–11 602 381)
Andean Latin America	43 (24–73)	0·6 (0·4–1·1)	224·4 (126·1–367·5)	1 472 041 (826 886–2 410 738)	127 (75–213)	0·2 (0·1–0·4)	81·1 (54·3–113·8)	4 849 344 (3 247 750–6 800 556)
Caribbean	104 (47–203)	2·6 (1·2–5·1)	145·4 (80·3–246·0)	599 771 (331 223–1 015 079)	211 (112–359)	0·5 (0·2–0·8)	47·2 (31·1–67·0)	2 164 245 (1 425 531–3 073 148)
Tropical Latin America	51 (24–89)	0·3 (0·2–0·6)	273·6 (149·7–453·2)	3 901 845 (2 134 792–6 462 186)	206 (120–306)	0·1 (0·1–0·1)	51·4 (33·3–74·2)	11 042 389 (7 153 152–15 919 339)
East Asia	1 (0–1)	0·0 (0·0–0·0)	0·3 (0·2–0·4)	17 775 (11 027–27 005)	3 (2–5)	0·0 (0·0–0·0)	0·1 (0·1–0·1)	151 553 (102 926–200 656)
Southeast Asia	390 (158–747)	0·7 (0·3–1·3)	82·0 (35·6–163·5)	4 820 721 (2 091 257–9 610 935)	1632 (777–2839)	0·2 (0·1–0·4)	30·8 (18·6–47·7)	20 197 076 (12 235 267–31 296 874)
Oceania	39 (17–78)	2·8 (1·2–5·5)	243·2 (135·9–412·8)	335 773 (187 598–569 904)	193 (100–325)	1·7 (0·9–2·9)	131·1 (85·8–185·6)	1 464 861 (959 231–2 073 550)
North Africa and Middle East	2076 (1063–3532)	3·3 (1·7–5·6)	266·0 (132·6–479·9)	16 894 231 (8 422 229–30 482 176)	2815 (1578–4584)	0·5 (0·3–0·8)	71·2 (43·2–110·4)	40 936 712 (24 862 627–63 496 927)
South Asia	4482 (2318–7382)	2·9 (1·5–4·8)	99·8 (58·7–161·4)	15 952 557 (9 373 596–25 785 888)	22 942 (10 613–42 231)	1·3 (0·6–2·5)	40·6 (27·1–57·1)	69 281 143 (46 178 724–97 325 901)
Southern sub-Saharan Africa	212 (80–387)	2·5 (0·9–4·5)	71·3 (36·6–123·1)	645 778 (331 917–1 116 070)	379 (142–666)	0·5 (0·2–0·9)	24·0 (14·0–37·0)	1 857 703 (1 081 496–2 868 515)
Western sub-Saharan Africa	5197 (2032–9574)	8·0 (3·1–14·8)	106·4 (47·5–198·0)	6 950 968 (3 099 062–12 929 044)	6487 (2746–11 454)	1·6 (0·7–2·9)	29·0 (15·7–48·6)	11 577 006 (6 265 456–19 407 424)
Eastern sub-Saharan Africa	5485 (2889–8941)	8·8 (4·6–14·3)	243·1 (126·8–426·8)	15 163 112 (7 912 373–26 621 991)	14 832 (8531–23 472)	3·8 (2·2–6·1)	93·4 (60·1–137·6)	36 127 390 (23 237 093–53 252 943)
Central sub-Saharan Africa	351 (46–811)	1·7 (0·2–3·9)	64·5 (24·0–126·6)	1 370 060 (508 515–2 688 078)	562 (91–1239)	0·5 (0·1–1·1)	19·4 (8·4–34·2)	2 299 091 (996 979–4 043 864)

**Figure 2:**
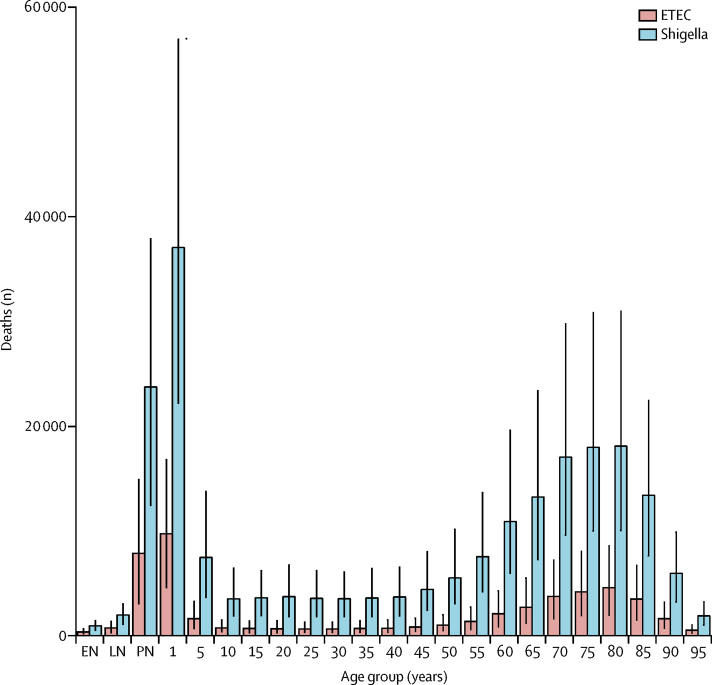
The age distribution of shigella and ETEC deaths globally in 2016 Error bars are 95% uncertainty interval. ETEC=enterotoxigenic *Escherichia coli*. EN=early neonatal. LN=late neonatal. PN=postnatal.

The greatest estimated number of under-5 deaths due to shigella was in western sub-Saharan Africa (29 027 deaths, 17 665–45 045) and the highest rates of mortality due to shigella in this age group were in sub-Saharan Africa, where mortality rates were greater than 10 per 100 000 people per year in northern, western, eastern, and central regions (table). Under-5 diarrhoea mortality attributable to shigella was lowest in western Europe. Shigella was isolated 1·98 (95% UI 1·63–2·34) times more frequently among patients admitted to hospital for diarrhoea than among patients with diarrhoea who were not admitted to hospital (appendix p 3). The global incidence of shigella-related diarrhoea among children younger than 5 years was 116·2 episodes per 1000 child-years (95% UI 64·3–198·6) and ranged from 0·2 episodes (0·1–1·1) in the high-income Asia-Pacific region to 345·3 episodes (199·9–564·1) in Oceania (table). Shigella was often associated with diarrhoeal burden and mortality across adult age groups, increasingly so among elderly people (figure 1, figure 2) and was the most common cause of diarrhoea among adults older than 70 years (74 400 deaths, 42 400–128 700).

ETEC was the eighth leading cause of diarrhoea mortality in 2016 among all age groups globally, accounting for an estimated 51 186 deaths (95% UI 26 757–83 064; table); about 3·2% (1·8–4·7) of all diarrhoea deaths were attributable to ETEC. The mortality rate for diarrhoea attributable to ETEC did not significantly differ between men and women (0·7 deaths [0·4–1·2] per 100 000 men and 0·7 deaths [0·3–1·3] per 100 000 women). Between 1990 and 2016, the diarrhoea mortality rate attributable to ETEC decreased faster than the rate attributable to shigella (60·6% decrease, 60·1–62·5) from 1·75 deaths per 100 000 (0·96–2·81) in 1990, to 0·69 deaths per 100 000 (0·36–1·12) in 2016. ETEC was responsible for a similar proportion of diarrhoea deaths among children younger than 5 years old (4·2%, 2·2–6·9) as it was in all ages (3·1%, 1·7–4·6; Figure 1, Figure 2), and was responsible for an estimated 18 669 deaths (9900–30 659) in this age group (table).

**Figure 3:**
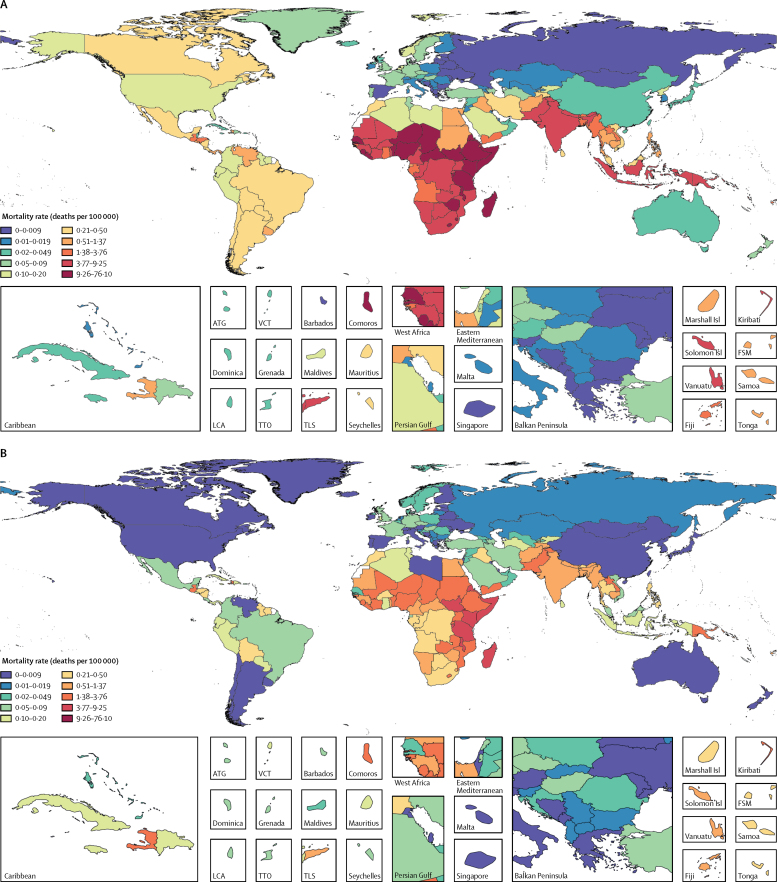
Shigella (A) and enterotoxigenic *Escherichia coli* (B) diarrhoea mortality rate per 100 000 people in 2016 for all ages ATG=Antigua and Barbuda. VCT=Saint Vincent and the Grenadines. LCA=Saint Lucia. TTO=Trinidad and Tobago. Isl=Islands. FSM=Federated States of Micronesia. TLS=Timor-Leste.

The greatest estimated number of under-5 deaths due to ETEC was in eastern sub-Saharan Africa (5485 deaths, 2889–8941) and the global mortality rate among children younger than 5 years ranged from less than 0·1 per 100 000 in many regions to 8·8 per 100 000 (4·6–14·3) in eastern sub-Saharan Africa (table, figure 3). The greatest number of deaths due to ETEC among all ages was in south Asia (table). ETEC was isolated 0·84 (0·71–0·98) times more frequently in patients admitted to hospital for diarrhoea than in patients who were not admitted to hospital for diarrhoea (appendix p 3). The incidence of ETEC-attributable diarrhoea among children younger than 5 years was 116·8 per 1000 child-years (61·7–202·6), which was similar to the incidence for shigella diarrhoea in this age group.

**Figure 4:**
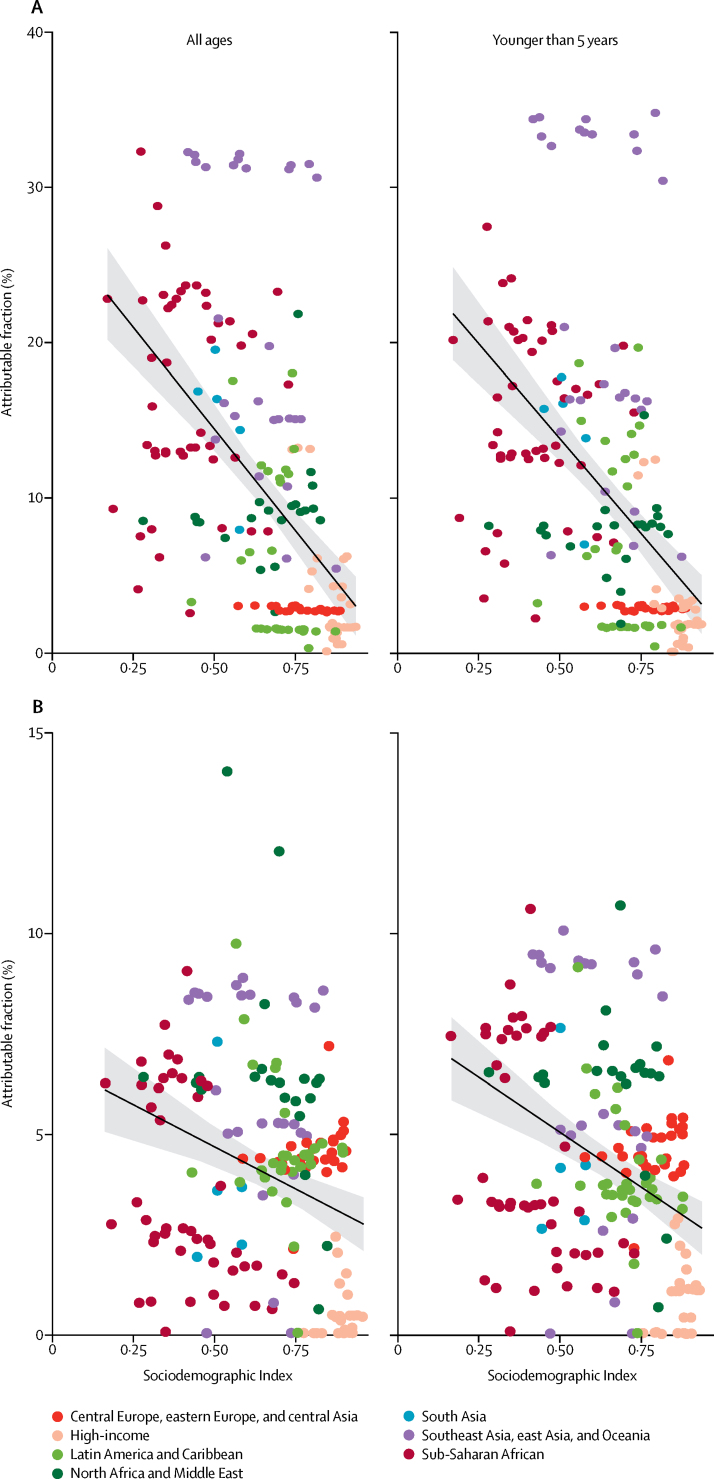
Shigella (A) and ETEC (B) population attributable fraction by sociodemographic index, 2016 Sociodemographic index is a measure of the relative development of a country, with high values indicating more development. Ribbons are 95% uncertainty intervals. Point colours are GBD super-regions. ETEC=enterotoxigenic *Escherichia coli*.

The burden of shigella and ETEC varied by geographical region (figure 3). Differences in the mortality rate by country and region depended on the population attributable fraction of diarrhoea (appendix pp 7–8) and on the underlying diarrhoea-related mortality rates. The mortality rates due to shigella were greater than for ETEC in nearly every super-region, except the two super-regions of central Europe, eastern Europe, and central Asia (0·036 ETEC deaths per 100 000 people, 95% UI 0·022–0·055; 0·024 shigella deaths per 100 000, 0·014–0·037), and north Africa and the Middle East (0·49 ETEC deaths per 100 000 people, 0·27–0·80; 0·48 shigella deaths per 100 000 people, 0·27–0·74). The proportion of diarrhoea deaths among all ages due to shigella ranged from less than 5% in Europe and central Asia to more than 15% in eastern and southern sub-Saharan Africa and southeast Asia (appendix p 8). The distribution of ETEC attributable fractions among all ages ranged from less than 2% in east Asia (China) to more than 11% in Tunisia and Sudan (appendix p 8). The attributable fraction of under-5 diarrhoea mortality was higher for shigella than for ETEC in most countries, with some exceptions in central and eastern Europe and in central Asia (figure 4, appendix p 8).

The use of bacterial culture to detect shigella and ETEC in diarrhoeal stool samples is likely to miss a substantial proportion of infections.^13^ Compared with the real-time PCR case definition, which was determined on the basis of the ability to discriminate between cases and controls in the Global Enteric Multicenter Study, the sensitivity of bacterial culture was 42% for shigella (95% UI 40–45) and 47% for ETEC (44–50). By contrast, the specificities for bacterial culture for shigella (99%, 98·7–99·3) and culture and molecular subtyping for ETEC (97%, 96·5–97·5) were high (appendix p 3). Testing for shigella by itself improved the frequency of detection, increasing the rate of detection by 89% (32–174; appendix p 3). We found that shigella was strongly associated with diarrhoea, particularly in people older than 1 year. The odds of symptomatic diarrhoea when shigella was detected in a stool sample was 3·47 (1·90–5·82) in children younger than 1 year and 6·33 (2·46–13·79) in those older than 1 year, suggesting that 71–84% of diarrhoea episodes with shigella detected in the stool samples are attributable to the pathogen (appendix p 3). ETEC was not as strongly associated with diarrhoea; the OR of diarrhoea when ETEC was detected in a stool sample was 1·65 (1·24–2·18) in children younger than 1 year and 2·08 (1·58–2·71) in those older than 1 year. Shigella is also strongly associated with severe diarrhoea; the frequency at which shigella was detected in patients admitted to hospital for diarrhoea was 98% (63–134) higher than in patients who were not admitted to hospital for diarrhoea (appendix p 3).

**Figure 5:**
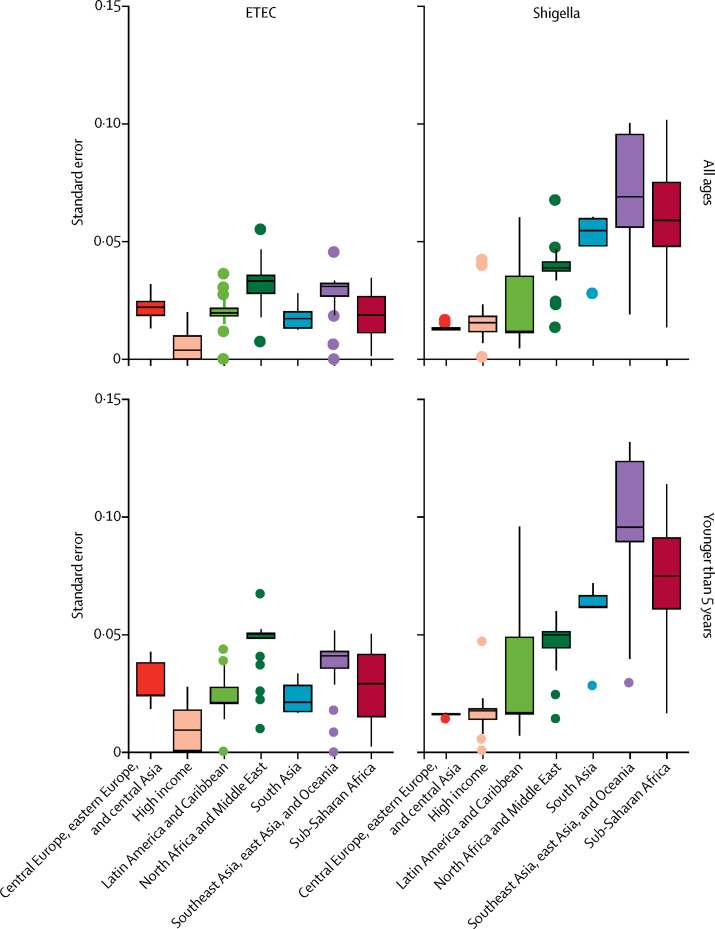
Association between standard error of PAF and GBD super region Data are the standard error of the mean PAF. ETEC=enterotoxigenic *Escherichia coli*. PAF=population attributable fraction.

A sociodemographic index was developed for GBD 2015 to measure the relative development of a country, with high values indicating more development.^48^ The proportion of diarrhoea deaths attributed to ETEC was moderately correlated with sociodemographic index (*r*^2^ −0·33, 95% UI −0·42 to −0·24). Shigella is strongly correlated (*r*^2^ −0·54, −0·60 to −0·46) with a highly negative slope, indicating that these causes, shigella especially, are focused in low-income countries (figure 4). The attributable fraction of shigella and ETEC was inversely related with the UIs for those estimates (figure 5). This finding highlights that the areas with the greatest burden of these two causes of diarrhoea are those with relatively poor health-care infrastructure, disease surveillance, and laboratory capacity for the detection of these pathogens.

## Discussion

Shigella and ETEC are two of the leading causes of diarrhoea mortality;^1^, ^2^ together they were responsible for more than 250 000 deaths in 2016 and about 20% of diarrhoea deaths worldwide. Other groups have estimated a substantial burden of shigella and ETEC diarrhoea among older children and adults^9^ and among children younger than 2 years^18^ that varies by location. Our study estimated 63 713 deaths from shigella and 18 669 deaths from ETEC among children younger than 5 years, and 74 402 deaths from shigella and 18 152 deaths from ETEC among adults older than 70 years. We show that the burden of diarrhoea attributable to shigella and ETEC, while decreasing, remains a substantial cause of mortality and disability globally. These results call for efforts to improve treatment, increase prevention, and reassess the effects on people older than 5 years.

Control of the burden of shigella is challenging for various reasons. First, shigella has a low infectious dose and is transmitted through the faecal-oral route via direct person-to-person transmission, contaminated food and water, and fomites.^49^ Second, the variety of shigella species and serotypes increases the possibility of reinfection.^50^ Shigella invades the mucosal lining of the colon and often causes dysentery that necessitates antibiotic therapy, not just oral rehydration, which further complicates treatment. However, dysentery is not quantified in GBD. Further, the emergence of multidrug-resistant strains of shigella threatens the administration of effective, affordable treatment and highlights the importance of infection prevention.^35^, ^36^, ^51^ The WHO's Global Antimicrobial Resistance Surveillance System^52^ identified shigella as a priority pathogen for the development of new interventions.

Our findings highlight the burden of shigella mortality in elderly people, which necessitates interventions that can decrease fatality, especially in high burden areas. Specialised improved quality of care with prompt rehydration, nutritional supplementation, and guidelines for the proper use of antibiotics when needed, can alleviate the high burden in this population and shigella case fatality in general. ETEC is primarily transmitted via food and water contaminated by faeces, causing secretory diarrhoea mediated by adherence (without invasion) and enterotoxin production within the small intestine. ETEC produce ST or LT enterotoxins, or both, which stimulate the release of fluid and electrolytes from the intestinal epithelium, resulting in watery diarrhoea.^16^

Diarrhoea early in childhood can impede the absorption of nutrients in the gut, leading to malnutrition.^27^, ^30^ Although many studies that analyse diarrhoea morbidity focus on all-cause diarrhoea, some pathogens appear to have a greater effect on childhood growth than others do. Both shigella and ETEC have been significantly associated with reduced linear growth per diarrhoeal episode.^53^, ^54^ Furthermore, ETEC and shigella were the diarrhoeal pathogens contributing the fourth and fifth most years livedigu with disability (YLDs), after rotavirus, *Campylobacter* spp, and adenovirus.^1^, ^47^ Consequently, prevention is crucial to address the overall burden.

Shigella affects people of all ages and is a predominant cause of diarrhoea mortality throughout adolescence and adulthood. Our analysis shows that shigella was the leading cause of death among adults older than 70 years. Although routine immunisation programmes are an attractive option for the prevention of shigella, our results suggest that such programmes might miss a substantial burden of shigella mortality in this age group.

The long-term solution for disease reduction is an integrated approach that includes improved water quality, sanitation and handwashing, optimised nutrition, better access to medical care, and vaccines. A combined shigella–ETEC vaccine is also being investigated, partly because both pathogens affect similar geographical settings and populations.^45^ However, the development of such a vaccine has been hampered by numerous technical challenges and an insufficient market for research and development. Vaccines against shigella and ETEC are expected to have benefits beyond the prevention or reduction of diarrhoea, yet data assessing the long-term economic and health effects of these two infections are currently more restricted than those measuring mortality are.^28^, ^30^

Our results differ from previous estimates in some respects. The Child Health Epidemiology Research Group—now called the Maternal and Child Epidemiology Estimation group (MCEE)^8^—estimated that, in 2010, 42 000 deaths (95% UI 20 000–76 000) among children younger than 5 years were due to ETEC and 28 000 deaths (12 000–53 000) were due to shigella, whereas GBD 2016 estimated that 28 300 deaths (15 000–46 000) were due to ETEC and 99 400 deaths (64 800–144 700) were due to shigella in 2010. There are several reasons for these differences. First, unlike GBD 2016, the MCEE approach was categorical; if a pathogen was present in a diarrhoeal stool sample, diarrhoea was attributed to that pathogen. Second, the MCEE approach used conventional bacterial culture methods for diagnostic detection, whereas GBD 2016 used molecular diagnostics. Finally, the envelope (ie, the total number of under-5 diarrhoea deaths) was different between the two groups.

A systematic reanalysis^13^ of the GEMS stool samples using PCR to detect shigella and ETEC is largely consistent with our findings. Shigella and ETEC were responsible for a similar fraction of severe diarrhoea episodes among children younger than 1 year, with a growing proportion due to shigella in children aged 1–2 years and 2–5 years in GEMS.^6^, ^13^ Our results, however, suggest that the attributable fraction is higher for shigella in all age categories than was reported in the systematic reanalysis,^13^ including the under-1 year age groups.^1^ Full details are given in the appendix.

Our findings have several limitations. First, our estimates of mortality, morbidity, and aetiological attribution for shigella and ETEC are restricted by availability of data, particularly data sparsity in regions of the world with a high diarrhoea burden. In addition, scarce data are available for the neonatal age group. Although adjustment for factors such as maternal immunity might help to improve our model estimates, quantification of the effect of maternal immunity is restricted by the availability of data. We account for this limitation by including UIs with each of our estimates, and our modelling approach allows us to make inferences for places and times with little data, based on more reliable estimates from other periods and regions to generate the best possible estimates. There is also a general scarcity of data on diarrhoea among populations older than 5 years and, although we model causes for diarrhoea in these age groups, the ORs from the oldest age group in GEMS—under 5 years old—are assumed to be representative in older ages. Second, this analysis only accounts for the acute phase of diarrhoea in our YLD estimates for the two pathogens. Consequently, our DALY estimates severely underestimate diarrhoea-associated long-term sequaelae, such as stunting and cognitive impairment.^28^ We plan to do more studies on this topic as more data are generated to inform these outcomes for shigella and ETEC diarrhoea, which will provide better estimates on the comprehensive burden of these pathogens.

In summary, our findings give an insight into the global burden of shigella and ETEC diarrhoea globally, spanning over 25 years for both sexes and all ages. Such refined burden estimates for the mortality, morbidity, and long-term effects of shigella and ETEC are needed to guide funders, public health officials, and policy makers. Refined burden estimates will help these individuals and organisations to make evidence-based decisions for the allocation of resources and the promotion of vaccine development and other effective strategies to reduce the unacceptable burden of diarrhoea worldwide.

For a **list of all GBD 2015 data sources for each country** see http://ghdx.healthdata.org/gbd-2015/data-input-sources

For **online results** see https://vizhub.healthdata.org/gbdcompare/ and https://ghdx.healthdata.org/gbd-2016/

For the **code** see http://ghdx.healthdata.org/global-burdendisease-study-2016-gbd-2016-causes-death-3

## Data sharing
