# Corrections

**DOI:** 10.1016/S1473-3099(18)30664-9

**Published:** 2018-12

*Khalil IA, Troeger T, Blacker BF, et al. Morbidity and mortality due to shigella and enterotoxigenic* Escherichia coli diarrhoea*: the Global Burden of Disease Study 1990–2016.* Lancet Infect Dis *2018;* 18: *1229–40*—In this Article, the copyright line should have been “© 2018 The Author(s). Published by Elsevier Ltd. This is an Open Access article under the CC BY 4.0 license.” This correction has been made to the online version as of Oct 30, 2018.

