# Supplementary material for: Morbidity and mortality due to shigella and enterotoxigenic Escherichia coli diarrhoea: the Global Burden of Disease Study 1990–2016
Source: Lancet Infect Dis. 2018 Nov;18(11):1229–40. doi: 10.1016/S1473-3099(18)30475-4 (PMC6202441; doi:10.1016/S1473-3099(18)30475-4)
Supplement: Supplementary appendix [file mmc1.pdf]

THE LANCET
Infectious Diseases

Supplementary appendix

This appendix formed part of the original submission and has been peer reviewed. We post it as supplied by the authors.

Supplement to: Khalil IA, Troeger C, Blacker BF, et al. Morbidity and mortality due to shigella and enterotoxigenic Escherichia coli diarrhoea: the Global Burden of Disease Study 1990–2016. *Lancet Infect Dis* 2018; published online Sept 25. http://dx.doi.org/10.1016/S1473-3099(18)30475-4.

Supplementary Appendix: The burden of *Shigella* and enterotoxigenic *E. coli*: an analysis from the Global Burden of Disease Study 2016

This document provides supplementary material on the data and input values used in modelling the global burden of diarrhoea due to *Shigella* and Enterotoxigenic *E. coli*. Additional details on the broader modelling of diarrhoea mortality and morbidity are available elsewhere.

General Global Burden of Disease information, including on statistical modelling platforms and core assumptions regarding the study, can be found in these references.[1–4]

Diarrhoea-specific modelling information and detail on assumptions regarding disease can be found in these references.[5,6]

Contents

Comparison with other estimates 2

Tables and Figures 3

Supplementary Table 1. Data points used for aetiology proportion modelling 3
Supplementary Table 2. Odds ratios reflecting the association between moderate-to-severe diarrhoea and Shigella and ETEC 3
Supplementary Table 3. Data sources used in proportion modelling 10
Supplementary Table 4. Estimated country-level results for Shigella diarrhoea in 2016 31
Supplementary Table 5. Estimated country-level results for ETEC diarrhoea in 2016 47

Supplementary Figure 1. Data coverage maps 4
Supplementary Figure 2. Diagnostic accuracy for *Shigella* and ETEC 5
Supplementary Figure 3. Association between Shigella and ST-ETEC gene target concentration and diarrhoea case status 6
Supplementary Figure 4. Estimated mortality rate per 100,000 in 2016 among children under-5 7
Supplementary Figure 5. The population attributable fraction among diarrhoea deaths among all ages in 2016 is shown for each country. A) Shigella, B) Enterotoxigenic E. coli. 8
Supplementary Figure 6. Comparison of the attributable fractions for Shigella and ETEC across different iterations of the GBD 9

References 64

Comparison with other estimates

The *Shigella* burden estimates generated in this analysis align well with major diarrhoeal disease studies like GEMS and MCEE. However, the ETEC estimates presented do not align well with the ETEC findings in these studies, where Shigella and ETEC burdens tended to be close. Both ETEC and Shigella were among the top 5-6 agents associated with MSD in GEMS and also were leading contributors to dysentery, dehydration, or hospitalization in MAL-ED and align more closely with WHO MCEE burden and mortality estimates. In general, there are difficulties in accurately detecting ETEC. Most countries do not have the capability to detect ETEC using culture-based methods linked to toxin/CFA detection based on bioassay and Mab reaction of colonies, or to utilise DNA-based detections on ETEC colonies isolated from stools.

In this q-PCR based analysis, LT-only ETEC strains were not considered a pathogen, and LT-only ETEC strains were associated with diarrhoea in some studies. Though molecular diagnostics detect more ETEC, we account for this by adjusting monomolecular diagnostic test results using the sensitivity and specificity of bacterial culture to the q-PCR case definition. This is reflected in the increasing attribution in GBD 2016 compared to GBD 2013, which used a non-molecular case definition based on conventional techniques.

In addition, the GEMs study, which provided much of the data this analysis used to provide pathogen-specific estimates, may have underestimated ETEC incidence by testing only 3 colonies from stool. Many experts have suggested that screening 5 colonies per stool may have been a more appropraite choice. A number of studies have shown that sensitivity improves as the number of colonies tested increases.[7,8]

Another factor in our lower ETEC burden estimation may be the use of molecular diagnostics as a gold standard. This strategy is a recent advancement and still being developed and used primarily in research settings. The relationship between conventional laboratory techniques and quantitative PCR used in the present study is informed by few studies.

Supplementary Table 1. Data points used for aetiology proportion modelling

The number and percent of data points coming from diarrhoeal stool samples in a hospitalized inpatient sample population, the number and percent of data points that are from studies that only investigated *Shigella* or ETEC as the exclusive diarrhoeal aetiology, and the number and percent of data points that are among children under-5 is shown.

Pathogen	Data points	Inpatient population Number (% of data points)	Single pathogen testing Number (% of data points)	Under-5 Number (% of data points)
Shigella	824	322 (39.1%)	34 (4.1%)	498 (60.4%)
ETEC	344	150 (43.6%)	16 (4.7%)	261 (75.9%)

Supplementary Table 2. The association of Shigella and ETEC with moderate-to-severe diarrhoea

Results are based on a molecular case definition from the Global Enteric Multicenter Study, the sensitivity and specificity of the bacterial culture diagnostic compared to the qPCR case definition, and the relative frequency of detection in studies investigating the pathogens independently and in inpatient populations. This table differentiates odds ratios between those under and 1 year and older. Values in parentheses are the 95% uncertainty intervals.

Pathogen	Odds ratio under-1	Odds ratio 1 and older	Sensitivity	Specificity	Single test	Inpatient population
Shigella	3.47 (1.90-5.82)	6.33 (2.46-13.79)	0.42 (0.40-0.45)	0.99 (0.987-0.993)	1.89 (1.32-2.74)	1.98 (1.63-2.34)
ETEC	1.65 (1.24-2.18)	2.08 (1.58-2.71)	0.47 (0.44-0.50)	0.97 (0.965-0.975)	0.68 (0.47-1.05)	0.84 (0.71 - 0.98)

Supplementary Figure 1. Data coverage maps

A) The number of data points in the *Shigella* proportion model; B) The number of data points in the ETEC proportion model. The data shown detail the proportion of diarrhoea episodes where the pathogen is present.

A)

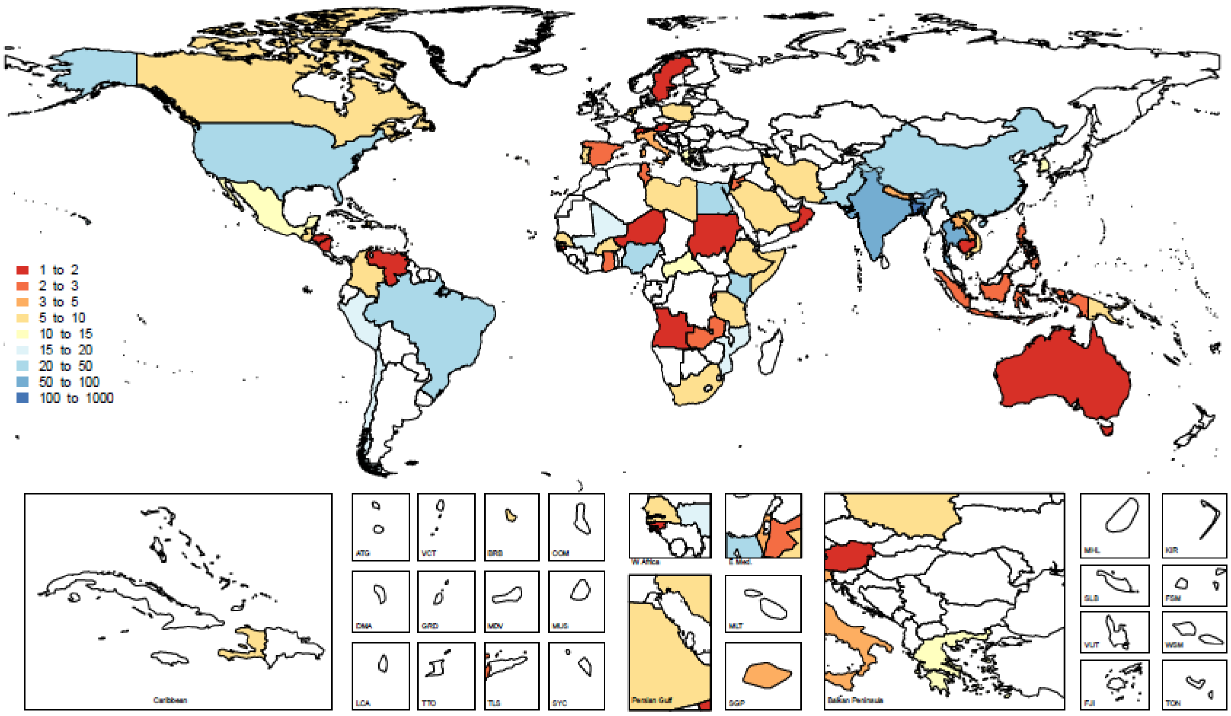

B)

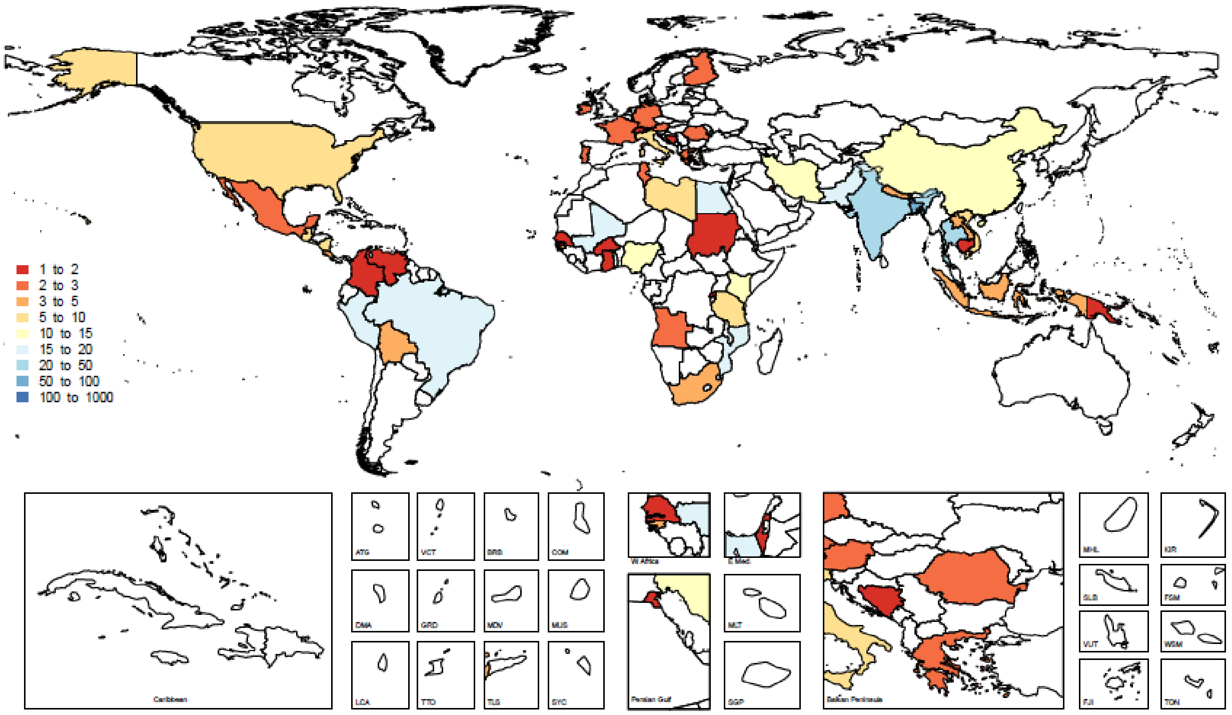

Supplementary Figure 2. Diagnostic accuracy for *Shigella* and ETEC

The diagnostic accuracy of the molecular diagnostic at varying values of the Cycle threshold (Ct) in discriminating between cases and controls in the Global Enteric Multicenter Study (GEMS). Higher Ct values indicate greater concentration of genetic target in the stool sample. The fitted curve is a loess curve and the vertical line indicates the point where the discriminatory accuracy between cases and controls is maximised. For ETEC, the vertical blue line is the smallest Ct where the discrimination is maximised.

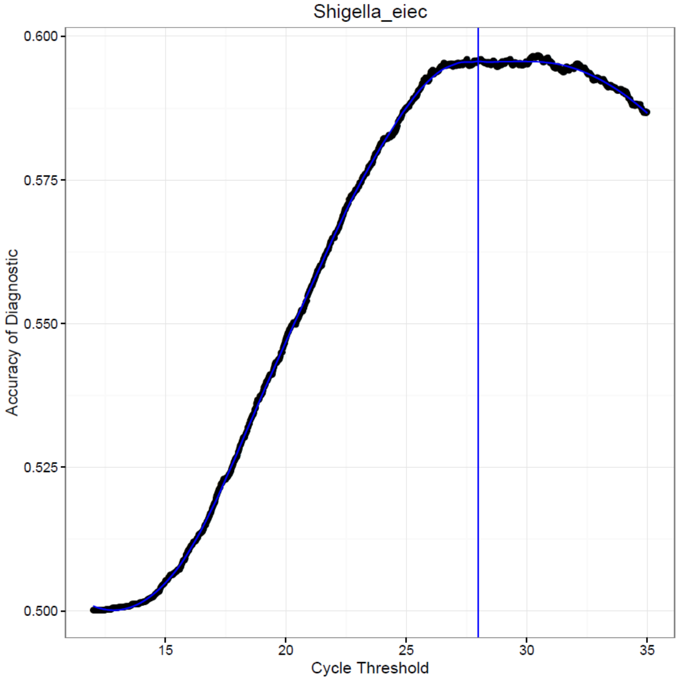

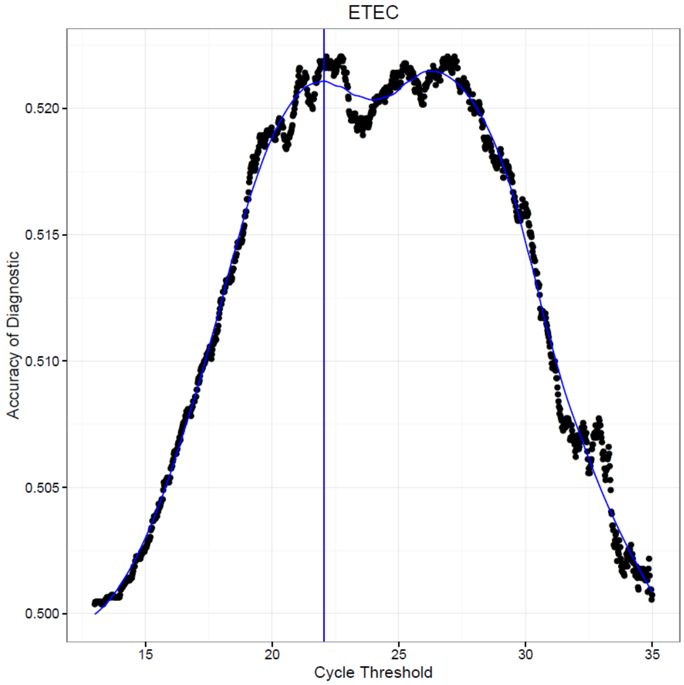

Supplementary Figure 3. Association between *Shigella* and ST-ETEC gene target concentration and diarrhoea case status

The Cycle threshold is a continuous test result from quantitative polymerase chain reaction. Lower Ct values indicate higher concentration of target in the sample so concentration decreases from left to right. The odds ratio represents the odds that a *Shigella* or ST-ETEC positive sample is from a diarrhoea case compared to a control sample. The trend is monotonic based on the Mann-Kendall test (p<0.001).

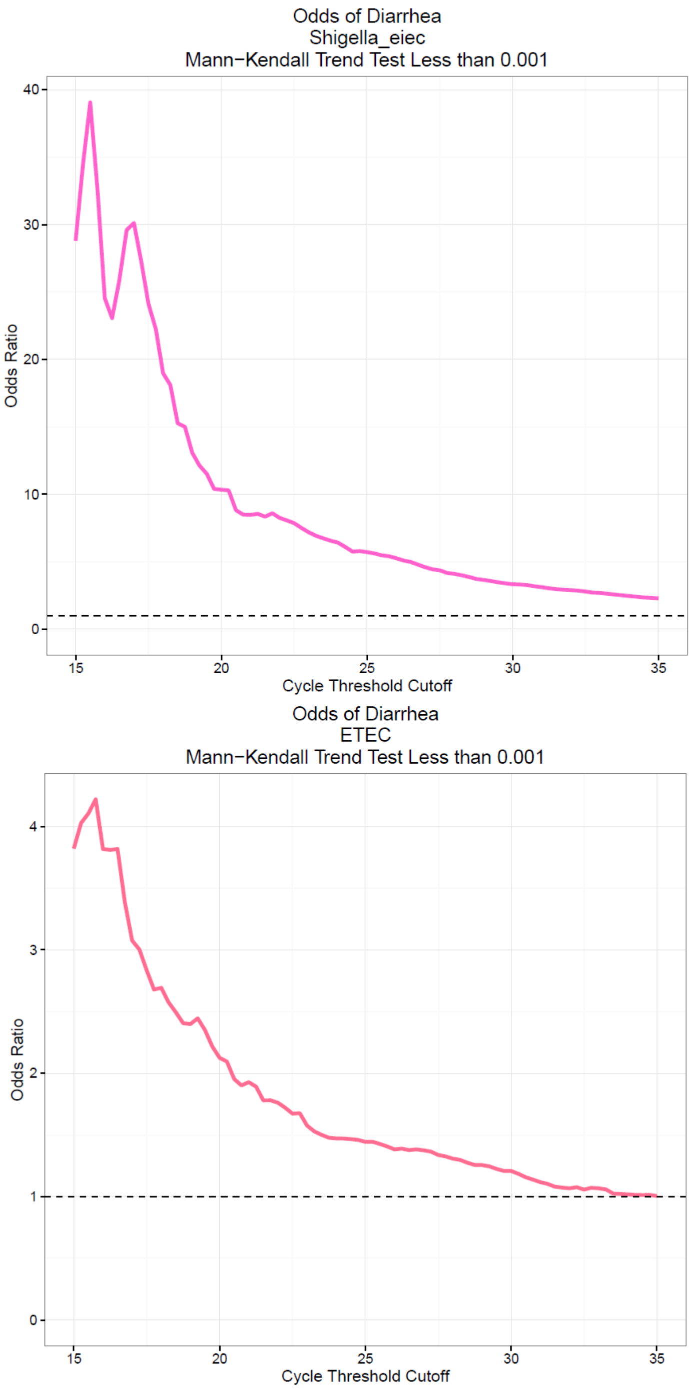

Supplementary Figure 4. Estimated mortality rate per 100,000 in 2016 among children under-5

This figure maps the estimated diarrhoea mortality rate attributable to A) *Shigella* in children under 5; B) ETEC in children under 5.

A)

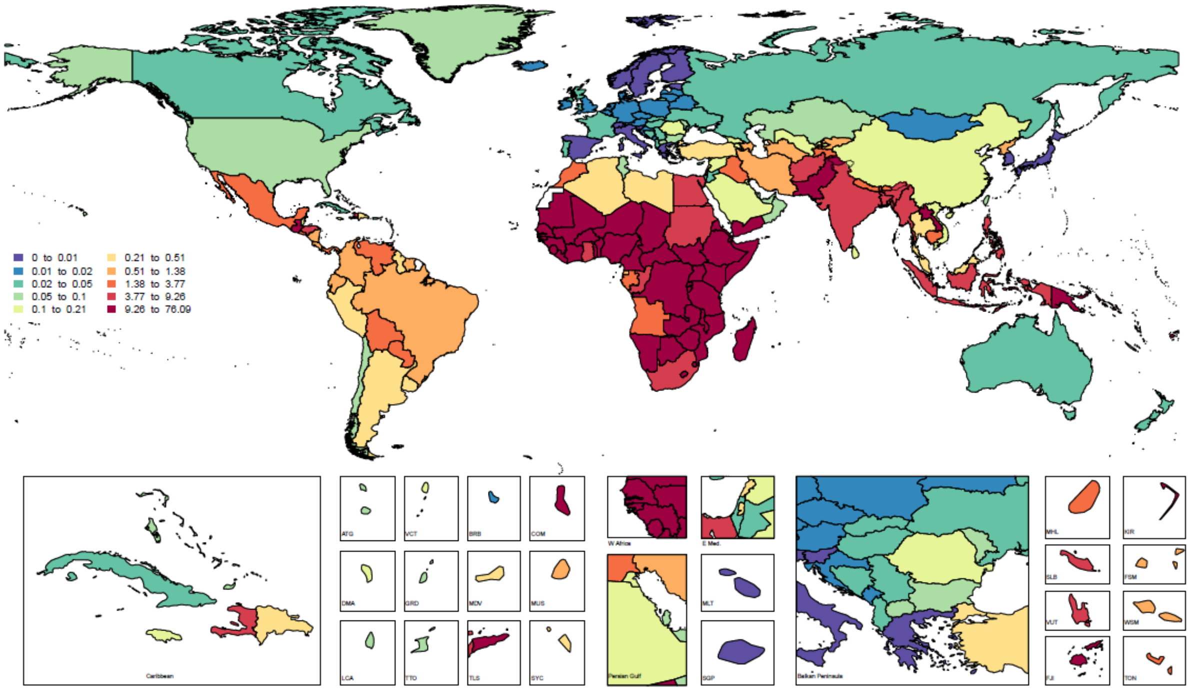

B)

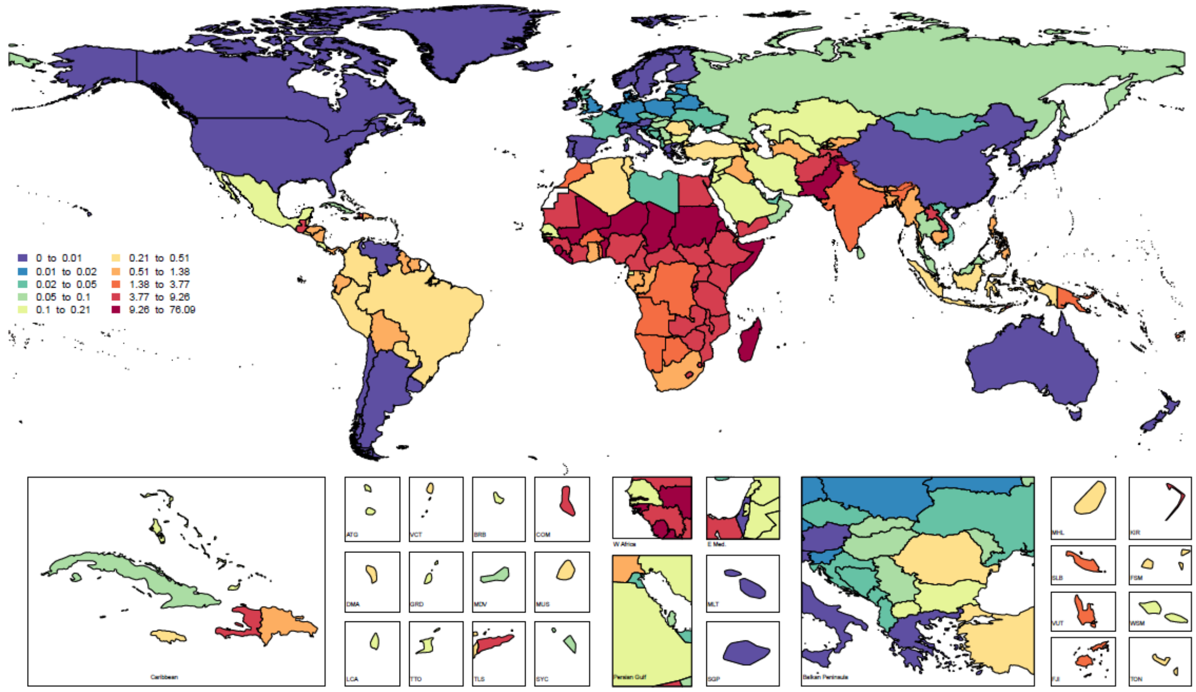

Supplementary Figure 5. The population attributable fraction among diarrhoea deaths among all ages in 2016 is shown for each country. A) Shigella, B) Enterotoxigenic E. coli.

A).

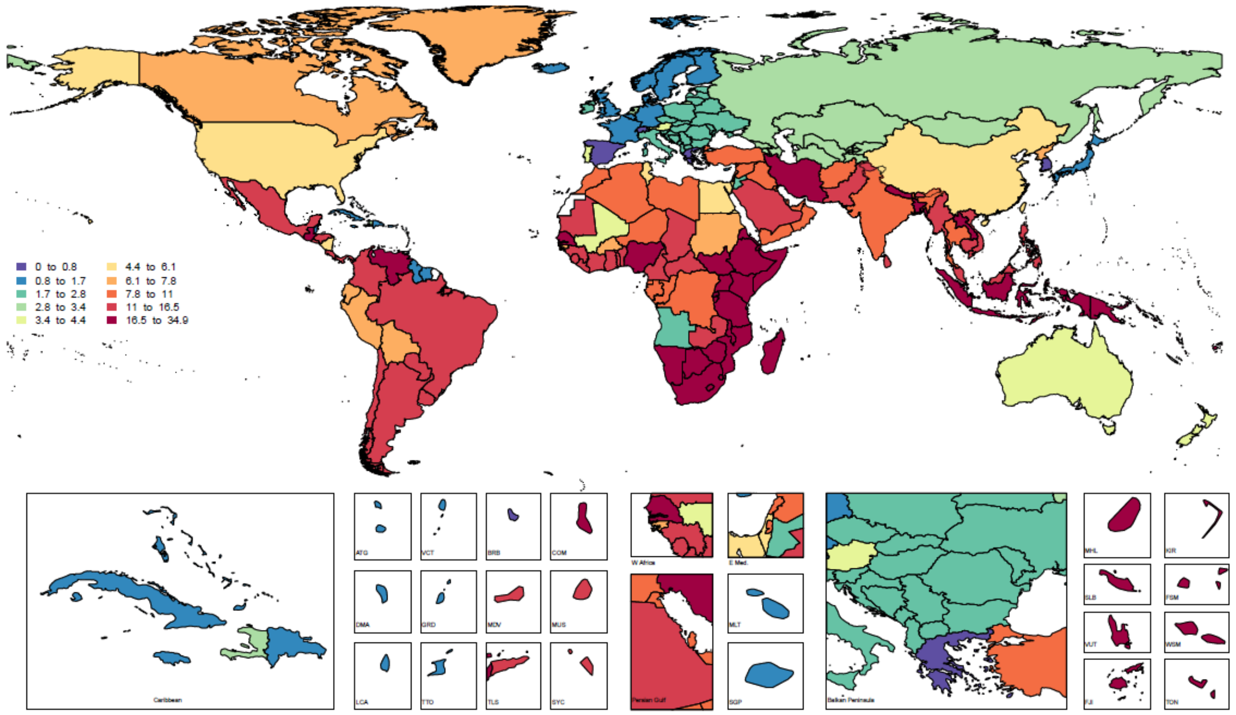

B)

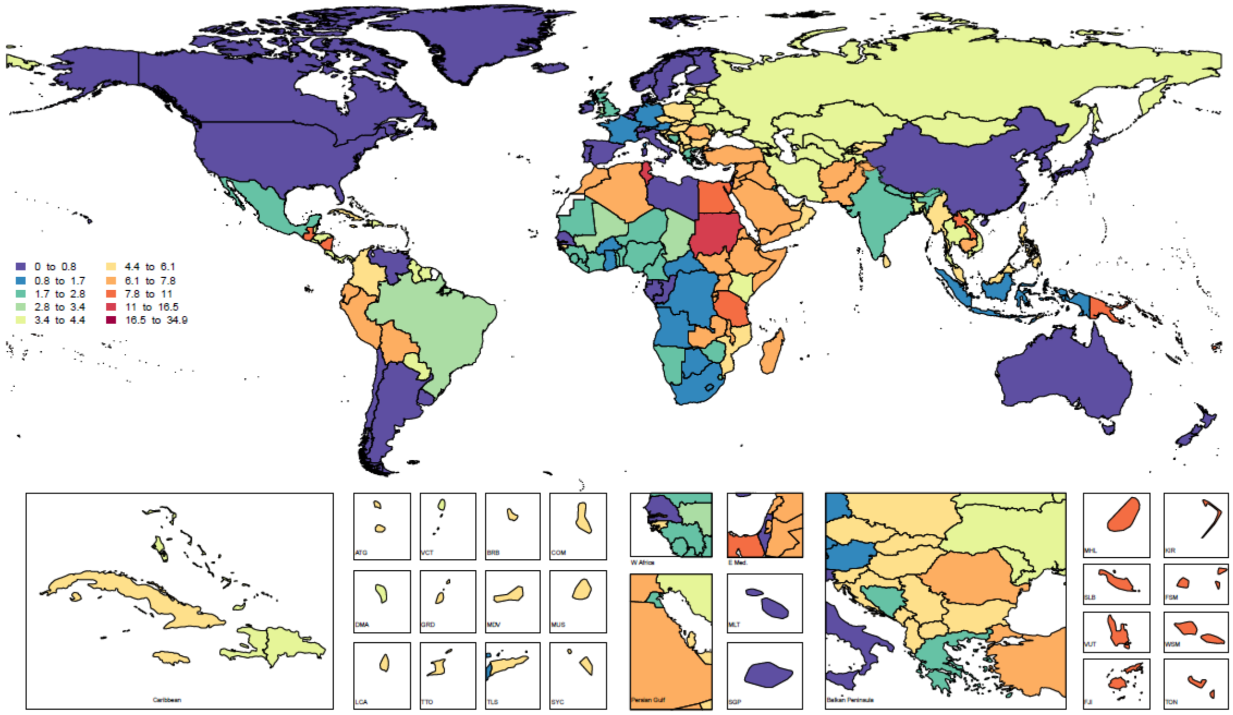

Supplementary Figure 6. Comparison of the attributable fractions for Shigella and ETEC across different iterations of the GBD

Starting in GBD 2015 and continuing for GBD 2016, the attributable fractions were based on a molecular diagnostic case definition. The large increase in the attribution for Shigella is related to the low sensitivity of bacterial culture compared to the molecular case definition.

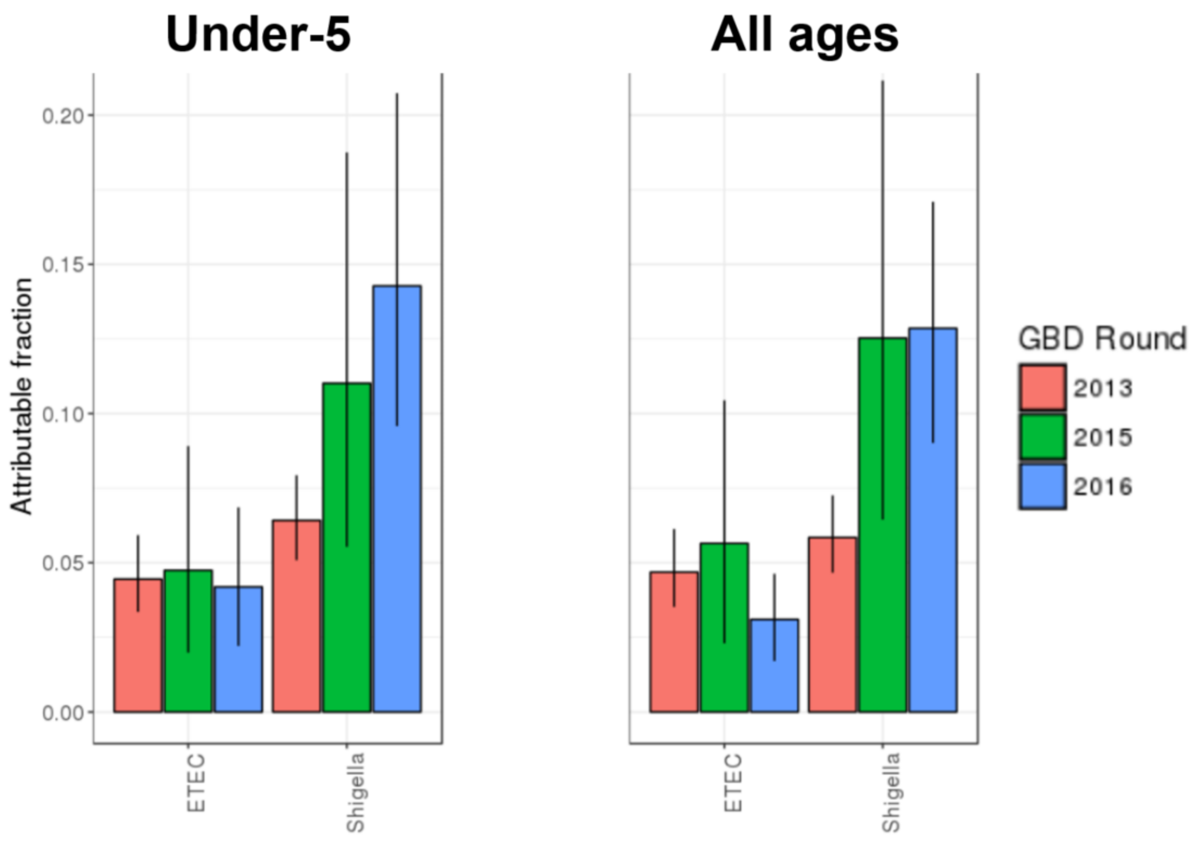

Supplementary Table 2. Data sources used in proportion modelling

A) Enterotoxigenic E. coli

Source
Aga Khan University, Center for Vaccine Development (Chile), Center for Vaccine Development, University of Maryland, Centers for Disease Control and Prevention (CDC), Department of Medical Microbiology and Immunology, Göteborg University, International Vaccine Institute, Perry Point Cooperative Studies Program Coordinating Center, U.S. Department of Veterans Affairs, School of Medicine, University of Virginia, University of Chile. Pakistan - Karachi Global Enteric Multicenter Study 2011-2013. Baltimore, MD, United States: Center for Vaccine Development, University of Maryland.
Aga Khan University, Fogarty International Center, National Institutes of Health (NIH), Foundation for the National Institutes of Health (FNIH). Pakistan - Naushahro Feroze Malnutrition and Enteric Disease Study 2009-2014.
Akinyemi KO, Oyefolu AO, Opere B, Otunba-Payne VA, Oworu AO. Escherichia coli in patients with acute gastroenteritis in Lagos, Nigeria. <i>East Afr Med J</i>. 1998; 75(9): 512-5.
Albert MJ, Faruque AS, Faruque SM, Sack RB, Mahalanabis D. Case-control study of enteropathogens associated with childhood diarrhea in Dhaka, Bangladesh. <i>J Clin Microbiol</i>. 1999; 37(11): 3458-64.
Albert MJ, Rotimi VO, Dhar R, Silpikurian S, Pacsa AS, Molla AM, Szucs G. Diarrhoeagenic Escherichia coli are not a significant cause of diarrhoea in hospitalised children in Kuwait. <i>BMC Microbiol</i>. 2009; 62.
Al-Gallas N, Bahri O, Bouratbeen A, Ben Haasen A, Ben Aissa R. Etiology of acute diarrhea in children and adults in Tunis, Tunisia, with emphasis on diarrheagenic Escherichia coli: prevalence, phenotyping, and molecular epidemiology. <i>Am J Trop Med Hyg</i>. 2007; 77(3): 571-82.
Ali MMM, Mohamed ZK, Klena JD, Ahmed SF, Moussa TAA, Ghenghesh KS. Molecular characterization of diarrheagenic Escherichia coli from Libya. <i>Am J Trop Med Hyg</i>. 2012; 86(5): 866-71.
Amisano G, Fornasero S, Migliaretti G, Caramello S, Tarasco V, Savino F. Diarrheagenic Escherichia coli in acute gastroenteritis in infants in North-West Italy. <i>New Microbiol</i>. 2011; 34(1): 45-51.
Ballard SB, Reaves EJ, Luna CG, Silva ME, Rocha C, Heitzinger K, Saito M, Apaza S, Espetia S, Blazes DL, Tilley DH, Guzman Aguilar RC, Gilman RH, Bausch DG. Epidemiology and Genetic Characterization of Noroviruses among Adults in an Endemic Setting, Peruvian Amazon Basin, 2004-2011. <i>PLoS One</i>. 2015; 10(7): e0131646.
Baqui AH, Sack RB, Black RE, Haider K, Hossain A, Alim AR, Yunus M, Chowdhury HR, Siddique AK. Enteropathogens associated with acute and persistent diarrhea in Bangladeshi children less than 5 years of age. <i>J Infect Dis</i>. 1992; 166(4): 792–6.
Bartlett AV, Torun B, Morales C, Cano F, Cruz JR. Oral gentamicin is not effective treatment for persistent diarrhea. <i>Acta Paediatr Suppl</i>. 1992; 381: 149-54.
Becker-Dreps S, Bucardo F, Vilchez S, Zambrana LE, Liu L, Weber DJ, Peña R, Barclay L, Vinjé J, Hudgens MG, Nordgren J, Svensson L, Morgan DR, Espinoza F, Paniagua M. Etiology of childhood diarrhea after rotavirus vaccine introduction: a prospective, population-based study in Nicaragua. <i>Pediatr Infect Dis J</i>. 2014; 33(11): 1156–63.
Bhan MK, Bhandari N, Sazawal S, Clemens J, Raj P, Levine MM, Kaper JB. Descriptive epidemiology of persistent diarrhoea among young children in rural northern India. <i>Bull World Health Organ</i>. 1989; 67(3): 281-8.
Bodhidatta L, Lan NTP, Hien BT, Lai NV, Srijan A, Serichantalergs O, Fukuda CD, Cam PD, Mason CJ. Rotavirus disease in young children from Hanoi, Vietnam. <i>Pediatr Infect Dis J</i>. 2007; 26(4): 325–8.
Bodhidatta L, McDaniel P, Sornsakrin S, Srijan A, Serichantalergs O, Mason CJ. Case-control study of diarrheal disease etiology in a remote rural area in Western Thailand. <i>Am J Trop Med Hyg</i>. 2010; 83(5): 1106-9.
Bonkoungou IJO, Haukka K, Österblad M, Hakanen AJ, Traoré AS, Barro N, Siitonen A. Bacterial and viral etiology of childhood diarrhea in Ouagadougou, Burkina Faso. <i>BMC Pediatr</i>. 2013; 13: 36.
Cama RI, Parashar UD, Taylor DN, Hickey T, Figueroa D, Ortega YR, Romero S, Perez J, Sterling CR, Gentsch JR, Gilman RH, Glass RI. Enteropathogens and other factors associated with severe disease in children with acute watery diarrhea in Lima, Peru. <i>J Infect Dis</i>. 1999; 179(5): 1139-44.

Caprioli A, Pezzella C, Morelli R, Giammanco A, Arista S, Crotti D, Facchini M, Guglielmetti P, Piersimoni C, Luzzi I. Enteropathogens associated with childhood diarrhea in Italy. The Italian Study Group on Gastrointestinal Infections. <i>Pediatr Infect Dis J</i>. 1996; 15(10): 876-83.

Carmona RCC, Timenetsky M do CST, da Silva FF, Granato CFH. Characterization of rotavirus strains from hospitalized and outpatient children with acute diarrhoea in São Paulo, Brazil. <i>J Med Virol</i>. 2004; 74(1): 166-72.

Center for Vaccine Development (Chile), Center for Vaccine Development, Ministry of Health (Mali), Center for Vaccine Development, University of Maryland, Centers for Disease Control and Prevention (CDC), Department of Medical Microbiology and Immunology, Göteborg University, International Vaccine Institute, Perry Point Cooperative Studies Program Coordinating Center, U.S. Department of Veterans Affairs, School of Medicine, University of Virginia, University of Chile. Mali - Bamako Global Enteric Multicenter Study 2011-2013.

Center for Vaccine Development (Chile), Center for Vaccine Development, University of Maryland, Centers for Disease Control and Prevention (CDC), Department of Medical Microbiology and Immunology, Göteborg University, International Centre for Diarrhoeal Disease Research, Bangladesh (ICDDR,B), International Vaccine Institute, Perry Point Cooperative Studies Program Coordinating Center, U.S. Department of Veterans Affairs, School of Medicine, University of Virginia, University of Chile. Bangladesh - Mizrapur Global Enteric Multicenter Study 2011-2013.

Center for Vaccine Development (Chile), Center for Vaccine Development, University of Maryland, Centers for Disease Control and Prevention (CDC), Department of Medical Microbiology and Immunology, Göteborg University, International Vaccine Institute, Kenya Medical Research Institute (KEMRI), Perry Point Cooperative Studies Program Coordinating Center, U.S. Department of Veterans Affairs, School of Medicine, University of Virginia, University of Chile. Kenya - Nyanza Global Enteric Multicenter Study 2011-2012.

Center for Vaccine Development (Chile), Center for Vaccine Development, University of Maryland, Centers for Disease Control and Prevention (CDC), Department of Medical Microbiology and Immunology, Göteborg University, International Vaccine Institute, Manhica Health Research Center (CISM), Perry Point Cooperative Studies Program Coordinating Center, U.S. Department of Veterans Affairs, School of Medicine, University of Virginia, University of Chile. Mozambique - Manhica Global Enteric Multicenter Study 2011-2013. Baltimore, MD, United States: Center for Vaccine Development, University of Maryland.

Center for Vaccine Development (Chile), Center for Vaccine Development, University of Maryland, Centers for Disease Control and Prevention (CDC), Department of Medical Microbiology and Immunology, Göteborg University, International Vaccine Institute, Medical Research Council (Gambia), Perry Point Cooperative Studies Program Coordinating Center, U.S. Department of Veterans Affairs, School of Medicine, University of Virginia, University of Chile. Gambia - Basse Global Enteric Multicenter Study 2011-2013.

Center for Vaccine Development (Chile), Center for Vaccine Development, University of Maryland, Centers for Disease Control and Prevention (CDC), Department of Medical Microbiology and Immunology, Göteborg University, International Vaccine Institute, National Institute of Cholera and Enteric Diseases (India), Perry Point Cooperative Studies Program Coordinating Center, U.S. Department of Veterans Affairs, School of Medicine, University of Virginia, University of Chile. India - Kolkata Global Enteric Multicenter Study 2011-2013.

Chandra BK, Singh G, Taneja N, Pahil S, Singhi S, Sharma M. Diarrhoeagenic Escherichia coli as a predominant cause of paediatric nosocomial diarrhoea in India. <i>J Med Microbiol</i>. 2012; 61(Pt 6): 830-6.

Chen Y, Chen X, Zheng S, Yu F, Kong H, Yang Q, Cui D, Chen N, Lou B, Li X, Tian L, Yang X, Xie G, Dong Y, Qin Z, Han D, Wang Y, Zhang W, Tang YW, Li L. Serotypes, genotypes and antimicrobial resistance patterns of human diarrhoeagenic Escherichia coli isolates circulating in southeastern China. <i>Clin Microbiol Infect</i>. 2014; 20(1): 52-8.

Chowdhury F, Rahman MA, Begum YA, Khan AI, Faruque ASG, Saha NC, Baby NI, Malek MA, Kumar AR, Svennerholm A-M, Pietroni M, Cravioto A, Qadri F. Impact of rapid urbanization on the rates of infection by Vibrio cholerae O1 and enterotoxigenic Escherichia coli in Dhaka, Bangladesh. <i>PLoS Negl Trop Dis</i>. 2011; 5(4): e999.

Christian Medical College, Vellore (India), Fogarty International Center, National Institutes of Health (NIH), Foundation for the National Institutes of Health (FNIH). India - Vellore Malnutrition and Enteric Disease Study 2009-2014.

Cruz JR, Cáceres P, Cano F, Flores J, Bartlett A, Torún B. Adenovirus types 40 and 41 and rotaviruses associated with diarrhea in children from Guatemala. <i>J Clin Microbiol</i>. 1990; 28(8): 1780-4.

Das SK, Ahmed S, Ferdous F, Farzana FD, Chisti MJ, Latham JR, Talukder KA, Rahman M, Begum YA, Qadri F, Faruque AS, Ahmed T. Etiological diversity of diarrhoeal disease in Bangladesh. <i>J Infect Dev Ctries</i>. 2013; 7(12): 900-9.

Dedeić-Ljubović A, Hukić M, Bekić D, Zvizdić A. Frequency and distribution of diarrhoeagenic Escherichia coli strains isolated from pediatric patients with diarrhoea in Bosnia and Herzegovina. <i>Bosn J Basic Med Sci</i>. 2009; 9(2): 148-55.

Do TT, Bui TTH, Mølbak K, Phung DC, Dalsgaard A. Epidemiology and aetiology of diarrhoeal diseases in adults engaged in wastewater-fed agriculture and aquaculture in Hanoi, Vietnam. <i>Trop Med Int Health</i>. 2007; 12(Suppl 2): 23-33.

Echeverria P, Hoge CW, Bodhidatta L, Tungtaem C, Herrmann J, Imlarp S, Tamura K. Etiology of diarrhea in a rural community in western Thailand: importance of enteric viruses and enterovirulent Escherichia coli. <i>J Infect Dis</i>. 1994; 169(4): 916-9.

Echeverria P, Taylor DN, Lexsomboon U, Bhaibulaya M, Blacklow NR, Tamura K, Sakazaki R. Case-control study of endemic diarrheal disease in Thai children. <i>J Infect Dis</i>. 1989; 159(3): 543-8.

El-Mohammady H, Mansour A, Shaheen HI, Henien NH, Motawea MS, Raafat I, Moustafa M, Adib-Messih IA, Sebeny PJ, Young SYN, Klena JD. Increase in the detection rate of viral and parasitic enteric pathogens among Egyptian children with acute diarrhea. <i>J Infect Dev Ctries</i>. 2012; 6(11): 774-81.

Essers B, Burnens AP, Lanfranchini FM, Somaruga SG, von Vigier RO, Schaad UB, Aebi C, Bianchetti MG. Acute community-acquired diarrhea requiring hospital admission in Swiss children. <i>Clin Infect Dis</i>. 2000; 31(1): 192–6.

Fang GD, Lima AA, Martins CV, Nataro JP, Guerrant RL. Etiology and epidemiology of persistent diarrhea in northeastern Brazil: a hospital-based, prospective, case-control study. <i>J Pediatr Gastroenterol Nutr</i>. 1995; 21(2): 137-44.

Faruque AS, Mahalanabis D, Islam A, Hoque SS, Hasnat A. Common diarrhea pathogens and the risk of dehydration in young children with acute watery diarrhea: a case-control study. <i>Am J Trop Med Hyg</i>. 1993; 49(1): 93-100.

Faruque ASG, Malek MA, Khan AI, Huq S, Salam MA, Sack DA. Diarrhoea in elderly people: aetiology, and clinical characteristics. <i>Scand J Infect Dis</i>. 2004; 36(3): 204-8.

Ferdous F, Ahmed S, Farzana FD, Das J, Malek MA, Das SK, Salam MA, Faruque ASG. Aetiologies of diarrhoea in adults from urban and rural treatment facilities in Bangladesh. <i>Epidemiol Infect</i>. 2015; 143(7): 1377–87.

Fogarty International Center, National Institutes of Health (NIH), Foundation for the National Institutes of Health (FNIH), Haydom Lutheran Hospital. Tanzania - Haydom Malnutrition and Enteric Disease Study 2009-2014.

Fogarty International Center, National Institutes of Health (NIH), Foundation for the National Institutes of Health (FNIH), Institute of Medicine, Tribhuvan University, University of Bergen, Walter Reed/AFRIMS Research Unit Nepal (WARUN). Nepal - Bhaktapur Malnutrition and Enteric Disease Study 2009-2014.

Fogarty International Center, National Institutes of Health (NIH), Foundation for the National Institutes of Health (FNIH), International Centre for Diarrhoeal Disease Research, Bangladesh (ICDDR,B). Bangladesh - Dhaka Malnutrition and Enteric Disease Study 2009-2014.

Fogarty International Center, National Institutes of Health (NIH), Foundation for the National Institutes of Health (FNIH), Johns Hopkins Bloomberg School of Public Health. Peru - Loreto Malnutrition and Enteric Disease Study 2009-2014.

Fogarty International Center, National Institutes of Health (NIH), Foundation for the National Institutes of Health (FNIH), National Institute of Science, Technology, and Biomedicine of Semi-Arid Brazil (INCT-IBISAB). Brazil - Fortaleza Malnutrition and Enteric Disease Study 2009-2014.

Fogarty International Center, National Institutes of Health (NIH), Foundation for the National Institutes of Health (FNIH), University of Venda. South Africa - Venda Malnutrition and Enteric Disease Study 2009-2014.

Gasparinho C, Mirante MC, Centeno-Lima S, Istrate C, Mayer AC, Tavira L, Nery SV, Brito M. Etiology of Diarrhea in Children Younger Than 5 Years Attending the Bengo General Hospital in Angola. <i>Pediatr Infect Dis J</i>. 2016; 35(2): e28-34.

Gomez-Duarte OG, Romero-Herazo YC, Paez-Canro CZ, Eslava-Schmalbach JH, Arzuza O. Enterotoxigenic Escherichia coli associated with childhood diarrhoea in Colombia, South America. <i>J Infect Dev Ctries</i>. 2013; 7(5): 372–81.

Gonzales L, Joffre E, Rivera R, Sj?ling •, Svennerholm AM, IÐiguez V. Prevalence, seasonality and severity of disease caused by pathogenic Escherichia coli in children with diarrhoea in Bolivia. <i>J Med Microbiol</i>. 2013; 62(Pt 11): 1697-706.

Gonzales L, Sanchez S, Zambrana S, Iñiguez V, Wiklund G, Svennerholm A-M, Sjöling A. Molecular characterization of enterotoxigenic Escherichia coli isolates recovered from children with diarrhea during a 4-year period (2007 to 2010) in Bolivia. <i>J Clin Microbiol</i>. 2013; 51(4): 1219-25.

Gosselin KB, Aboud S, McDonald CM, Moyo S, Khavari N, Manji K, Kisenge R, Fawzi W, Kellogg M, Tran HQ, Kibiki G, Gratz J, Liu J, Gewirtz A, Houpt E, Duggan C. Etiology of Diarrhea, Nutritional Outcomes and Novel Intestinal Biomarkers in Tanzanian Infants: A Preliminary Study. <i>J Pediatr Gastroenterol Nutr</i>. 2016.

Greenberg BL, Sack RB, Salazar-Lindo E, Budge E, Gutierrez M, Campos M, Visberg A, Leon-Barua R, Yi A, Maurutia D. Measles-associated diarrhea in hospitalized children in Lima, Peru: pathogenic agents and impact on growth. <i>J Infect Dis</i>. 1991; 163(3): 495-502.

Haghi F, Zeighami H, Hajiahmadi F, Khoshvaght H, Bayat M. Frequency and antimicrobial resistance of diarrhoeagenic Escherichia coli from young children in Iran. <i>J Med Microbiol</i>. 2014; 63(Pt 3): 427-32.

Haque R, Mondal D, Kirkpatrick BD, Akther S, Farr BM, Sack RB, Petri WA Jr. Epidemiologic and clinical characteristics of acute diarrhea with emphasis on Entamoeba histolytica infections in preschool children in an urban slum of Dhaka, Bangladesh. <i>Am J Trop Med Hyg</i>. 2003; 69(4): 398-405.

Hegde A, Ballal M, Shenoy S. Detection of diarrheagenic Escherichia coli by multiplex PCR. <i>Indian J Med Microbiol</i>. 2012; 30(3): 279-84.

Henry FJ, Udoy AS, Wanke CA, Aziz KM. Epidemiology of persistent diarrhea and etiologic agents in Mirzapur, Bangladesh. <i>Acta Paediatr Suppl</i>. 1992; 381: 27-31.

Hien BTT, Scheutz F, Cam PD, Serichantalergs O, Huong TT, Thu TM, Dalsgaard A. Diarrheagenic Escherichia coli and Shigella strains isolated from children in a hospital case-control study in Hanoi, Vietnam. <i>J Clin Microbiol</i>. 2008; 46(3): 996–1004.

Jafari F, Garcia-Gil LJ, Salmanzadeh-Ahrabi S, Shokrzadeh L, Aslani MM, Pourhoseingholi MA, Derakhshan F, Zali MR. Diagnosis and prevalence of enteropathogenic bacteria in children less than 5 years of age with acute diarrhea in Tehran children's hospitals. <i>J Infect</i>. 2009; 58(1): 21-7.

Jafari F, Shokrzadeh L, Hamidian M, Salmanzadeh-Ahrabi S, Zali MR. Acute diarrhea due to enteropathogenic bacteria in patients at hospitals in Tehran. <i>Jpn J Infect Dis</i>. 2008; 61(4): 269–73.

Jiraphongsa C, Bresee JS, Pongsuwanna Y, Kluabwang P, Poonawagul U, Arporntip P, Kanoksil M, Premsri N, Intusoma U. Epidemiology and burden of rotavirus diarrhea in Thailand: results of sentinel surveillance. <i>J Infect Dis</i>. 2005; 192(Suppl 1): S87-93.

Kabayiza J-C, Andersson ME, Nilsson S, Bergström T, Muhirwa G, Lindh M. Real-time PCR identification of agents causing diarrhea in Rwandan children less than 5 years of age. <i>Pediatr Infect Dis J</i>. 2014; 33(10): 1037–42.

Kakai R, Wamola IA, Bwayo JJ, Ndinya-Achola JO. Enteric pathogens in malnourished children with diarrhoea. <i>East Afr Med J</i>. 1995; 72(5): 288-9.

Kattula D, Francis MR, Kulinkina A, Sarkar R, Mohan VR, Babji S, Ward HD, Kang G, Balraj V, Naumova EN. Environmental predictors of diarrhoeal infection for rural and urban communities in south India in children and adults. <i>Epidemiol Infect</i>. 2015; 143(14): 3036-47.

Lanata CF, Black RE, Maúrtua D, Gil A, Gabilondo A, Yi A, Miranda E, Gilman RH, León-Barúa R, Sack RB. Etiologic agents in acute vs persistent diarrhea in children under three years of age in peri-urban Lima, Per. <i>Acta Paediatr Suppl</i>. 1992; 32-8.

Leung DT, Das SK, Malek MA, Qadri F, Faruque ASG, Ryan ET. Impact of Ramadan on clinical and microbiologic parameters of patients seen at a diarrheal hospital in urban Dhaka, Bangladesh, 1996-2012. <i>Am J Trop Med Hyg</i>. 2014; 90(2): 294–8.

Li J, Hu J, Wu H, Pan H, Zhang X, Xue Y, Wu F. [Etiological surveillance for diarrhea in Shanghai from August 2013 to July 2014]. <i>Chin J Epidemiol</i>. 2015; 36(10): 1099-103.

Lima AA, Moore SR, Barboza MS Jr, Soares AM, Schleupner MA, Newman RD, Sears CL, Nataro JP, Fedorko DP, Wuhib T, Schorling JB, Guerrant RL. Persistent diarrhea signals a critical period of increased diarrhea burdens and nutritional shortfalls: a prospective cohort study among children in northeastern Brazil. <i>J Infect Dis</i>. 2000; 181(5): 1643-51.

Lozer DM, Souza TB, Monfardini MV, Vicentini F, Kitagawa SS, Scaletsky IC, Spano LC. Genotypic and phenotypic analysis of diarrheagenic Escherichia coli strains isolated from Brazilian children living in low socioeconomic level communities. <i>BMC Infect Dis</i>. 2013; 418.

Mansour A, Shaheen HI, Amine M, Hassan K, Sanders JW, Riddle MS, Armstrong AW, Svennerholm AM, Sebeny PJ, Klena JD, Young SY, Frenck RW. Pathogenicity and phenotypic characterization of enterotoxigenic Escherichia coli isolates from a birth cohort of children in rural Egypt. <i>J Clin Microbiol</i>. 2014; 52(2): 587-91.

Meng CY, Smith BL, Bodhidatta L, Richard SA, Vansith K, Thy B, Srijan A, Serichantalergs O, Mason CJ. Etiology of diarrhea in young children and patterns of antibiotic resistance in Cambodia. <i>Pediatr Infect Dis J</i>. 2011; 30(4): 331-5.

Mishra OP, Dhawan T, Singla PN, Dixit VK, Arya NC, Nath G. Endoscopic and histopathological evaluation of preschool children with chronic diarrhoea. <i>J Trop Pediatr</i>. 2001; 47(2): 77-80.

Mølbak K, Wested N, Højlyng N, Scheutz F, Gottschau A, Aaby P, da Silva AP. The etiology of early childhood diarrhea: a community study from Guinea-Bissau. <i>J Infect Dis</i>. 1994; 169(3): 581-7.

Nair GB, Ramamurthy T, Bhattacharya MK, Krishnan T, Ganguly S, Saha DR, Rajendran K, Manna B, Ghosh M, Okamoto K, Takeda Y. Emerging trends in the etiology of enteric pathogens as evidenced from an active surveillance of hospitalized diarrhoeal patients in Kolkata, India. <i>Gut Pathog</i>. 2010; 2(1): 4.

Nakano T, Binka FN, Afari EA, Agbodaze D, Aryeetey ME, Mingle JA, Kamiya H, Sakurai M. Survey of enteropathogenic agents in children with and without diarrhoea in Ghana. <i>J Trop Med Hyg</i>. 1990; 93(6): 408-12.

Nasrin D, Wu Y, Blackwelder WC, Farag TH, Saha D, Sow SO, Alonso PL, Breiman RF, Sur D, Faruque ASG, Zaidi AKM, Biswas K, Van Eijk AM, Walker DG, Levine MM, Kotloff KL. Health care seeking for childhood diarrhea in developing countries: evidence from seven sites in Africa and Asia. <i>Am J Trop Med Hyg</i>. 2013; 89(1 Suppl): 3-12.

Nath G, Choudhury A, Shukla BN, Singh TB, Reddy DC. Significance of Cryptosporidium in acute diarrhoea in North-Eastern India. <i>J Med Microbiol</i>. 1999; 48(6): 523-6.

Nazarian S, Gargari SL, Rasooli I, Alerasol M, Bagheri S, Alipoor SD. Prevalent phenotypic and genotypic profile of enterotoxigenic Escherichia coli among Iranian children. <i>Jpn J Infect Dis</i>. 2014; 67(2): 78-85.

Ngan PK, Khanh NG, Tuong CV, Quy PP, Anh DN, Thuy HT. Persistent diarrhea in Vietnamese children: a preliminary report. <i>Acta Paediatr</i>. 1992; 81 Suppl 381: 124-6.

Nguyen TV, Le Van P, Le Huy C, Gia KN, Weintraub A. Detection and characterization of diarrheagenic Escherichia coli from young children in Hanoi, Vietnam. <i>J Clin Microbiol</i>. 2005; 43(2): 755–60.

Nhampossa T, Mandomando I, Acacio S, Quinto L, Vubil D, Ruiz J, Nhalungo D, Sacoor C, Nhabanga A, Nhacolo A, Aide P, Machevo S, Sigauque B, Nhama A, Kotloff K, Farag T, Nasrin D, Bassat Q, Macete E, Levine MM, Alonso P. Diarrheal Disease in Rural Mozambique: Burden, Risk Factors and Etiology of Diarrheal Disease among Children Aged 0-59 Months Seeking Care at Health Facilities. <i>PLoS One</i>. 2015; 10(5): e0119824.

Oberhelman RA, Gilman RH, Sheen P, Taylor DN, Black RE, Cabrera L, Lescano AG, Meza R, Madico G. A placebo-controlled trial of Lactobacillus GG to prevent diarrhea in undernourished Peruvian children. <i>J Pediatr</i>. 1999; 134(1): 15-20.

Ochoa TJ, Ecker L, Barletta F, Mispireta ML, Gil AI, Contreras C, Molina M, Amemiya I, Verastegui H, Hall ER, Cleary TG, Lanata CF. Age-related susceptibility to infection with diarrheagenic Escherichia coli among infants from Periurban areas in Lima, Peru. <i>Clin Infect Dis</i>. 2009; 49(11): 1694-702.

Odetoyin BW, Hofmann J, Aboderin AO, Okeke IN. Diarrhoeagenic Escherichia coli in mother-child Pairs in Ile-Ife, South Western Nigeria. <i>BMC Infect Dis</i>. 2016; 16: 28.

Okeke IN, Lamikanra A, Steinrück H, Kaper JB. Characterization of Escherichia coli strains from cases of childhood diarrhea in provincial southwestern Nigeria. <i>J Clin Microbiol</i>. 2000; 38(1): 7–12.

Onanuga A, Igbeneghu O, Lamikanra A. A study of the prevalence of diarrhoeagenic Escherichia coli in children from Gwagwalada, Federal Capital Territory, Nigeria. <i>Pan Afr Med J</i>. 2014; 17: 146.

O'Reilly CE, Jaron P, Ochieng B, Nyaguara A, Tate JE, Parsons MB, Bopp CA, Williams KA, Vinjé J, Blanton E, Wannemuehler KA, Vulule J, Laserson KF, Breiman RF, Feikin DR, Widdowson M-A, Mintz E. Risk factors for death among children less than 5 years old hospitalized with diarrhea in rural western Kenya, 2005-2007: a cohort study. <i>PLoS Med</i>. 2012; 9(7): e1001256.

Osawa K, Raharjo D, Wasito EB, Harijono S, Shigemura K, Osawa R, Sudarmo SM, Iijima Y, Shirakawa T. Frequency of diarrheagenic Escherichia coli among children in Surabaya, Indonesia. <i>Jpn J Infect Dis</i>. 2013; 66(5): 446-8.
Paniagua GL, Monroy E, García-González O, Alonso J, Negrete E, Vaca S. Two or more enteropathogens are associated with diarrhoea in Mexican children. <i>Ann Clin Microbiol Antimicrob</i>. 2007; 6: 17.
Patzi-Vargas S, Zaidi MB, Perez-Martinez I, León-Cen M, Michel-Ayala A, Chaussabel D, Estrada-Garcia T. Diarrheagenic Escherichia coli carrying supplementary virulence genes are an important cause of moderate to severe diarrhoeal disease in Mexico. <i>PLoS Negl Trop Dis</i>. 2015; 9(3): e0003510.
Pazzaglia G, Sack RB, Salazar E, Yi A, Chea E, Leon-Barua R, Guerrero CE, Palomino J. High frequency of coinfecting enteropathogens in Aeromonas-associated diarrhea of hospitalized Peruvian infants. <i>J Clin Microbiol</i>. 1991; 29(6): 1151-6.
Penny ME, Paredes P, Brown KH, Laughan B, Smith H. Lack of a role of the duodenal microflora in pathogenesis of persistent diarrhea and diarrhea-related malabsorption in Peruvian children. <i>Pediatr Infect Dis J</i>. 1990; 9(7): 479-87.
Platts-Mills JA, Gratz J, Mduma E, Svensen E, Amour C, Liu J, Maro A, Saidi Q, Swai N, Kumburu H, McCormick BJ, Kibiki G, Houpt ER. Association between stool enteropathogen quantity and disease in Tanzanian children using TaqMan array cards: a nested case-control study. <i>Am J Trop Med Hyg</i>. 2014; 90(1): 133-8.
Punyaratabandhu P, Vathanophas K, Varavithya W, Sangchai R, Athipanyakom S, Echeverria P, Wasi C. Childhood diarrhoea in a low-income urban community in Bangkok: incidence, clinical features, and child caretaker's behaviours. <i>J Diarrhoeal Dis Res</i>. 1991; 9(3): 244-9.
Qadri F, Saha A, Ahmed T, Al Tarique A, Begum YA, Svennerholm AM. Disease burden due to enterotoxigenic Escherichia coli in the first 2 years of life in an urban community in Bangladesh. <i>Infect Immun</i>. 2007; 75(8): 3961-8.
Qu M, Deng Y, Zhang X, Liu G, Huang Y, Lin C, Li J, Yan H, Li X, Jia L, Kan B, Huang F, Wang Q. Etiology of acute diarrhea due to enteropathogenic bacteria in Beijing, China. <i>J Infect</i>. 2012; 65(3): 214-22.
Ratchtrachenchai O-A, Subpasu S, Hayashi H, Ba-Thein W. Prevalence of childhood diarrhoea-associated Escherichia coli in Thailand. <i>J Med Microbiol</i>. 2004; 53(Pt 3): 237–43.
Rivera FP, Ochoa TJ, Maves RC, Bernal M, Medina AM, Meza R, Barletta F, Mercado E, Ecker L, Gil AI, Hall ER, Huicho L, Lanata CF. Genotypic and phenotypic characterization of enterotoxigenic Escherichia coli strains isolated from Peruvian children. <i>J Clin Microbiol</i>. 2010; 48(9): 3198-203.
Rodas C, Mamani R, Blanco J, Blanco JE, Wiklund G, Svennerholm A-M, Sjöling A, Iniguez V. Enterotoxins, colonization factors, serotypes and antimicrobial resistance of enterotoxigenic Escherichia coli (ETEC) strains isolated from hospitalized children with diarrhea in Bolivia. <i>Braz J Infect Dis</i>. 2011; 15(2): 132-7.
Saeed A, Abd H, Sandstrom G. Microbial aetiology of acute diarrhoea in children under five years of age in Khartoum, Sudan. <i>J Med Microbiol</i>. 2015; 64(Pt 4): 432–7.
Saha MR, Sen D, Datta P, Datta D, Pal SC. Role of rotavirus as the cause of acute paediatric diarrhoea in Calcutta. <i>Trans R Soc Trop Med Hyg</i>. 1984; 78(6): 818-20.
Saidi SM, Iijima Y, Sang WK, Mwangudza AK, Oundo JO, Taga K, Aihara M, Nagayama K, Yamamoto H, Waiyaki PG, Honda T. Epidemiological study on infectious diarrheal diseases in children in a coastal rural area of Kenya. <i>Microbiol Immunol</i>. 1997; 41(10): 773-8.
Sherchand JB, Tandukar S, Sherchan JB, Rayamajhi A, Gurung B, Shrestha L, Rijal B, Pokhrel BM. Hospital-based study in children with rotavirus gastroenteritis and other enteropathogens. <i>J Nepal Health Res Counc</i>. 2012; 10(21): 130-5.
Sire J-M, Garin B, Chartier L, Fall NK, Tall A, Seck A, Weill F-X, Breurec S, Vray M. Community-acquired infectious diarrhoea in children under 5 years of age in Dakar, Senegal. <i>Paediatr Int Child Health</i>. 2013; 33(3): 139–44.
Sobel J, Gomes TAT, Ramos RTS, Hoekstra M, Rodrigue D, Rassi V, Griffin PM. Pathogen-specific risk factors and protective factors for acute diarrheal illness in children aged 12-59 months in São Paulo, Brazil. <i>Clin Infect Dis</i>. 2004; 38(11): 1545-51.
Soli KW, Maure T, Kas MP, Bande G, Bebes S, Luang-Suarkia D, Siba PM, Morita A, Umezaki M, Greenhill AR, Horwood PF. Detection of enteric viral and bacterial pathogens associated with paediatric diarrhoea in Goroka, Papua New Guinea. <i>Int J Infect Dis</i>. 2014; 27: 54-58.
Spina A, Kerr KG, Cormican M, Barbut F, Eigentler A, Zerva L, Tassios P, Popescu GA, Rafila A, Eerola E, Batista J, Maass M, Aschbacher R, Olsen KEP, Allerberger F. Spectrum of enteropathogens detected by the

FilmArray GI Panel in a multicentre study of community-acquired gastroenteritis. <i>Clin Microbiol Infect</i>. 2015; 21(8): 719–28.
Stockmann C, Pavia AT, Graham B, Vaughn M, Crisp R, Poritz MA, Thatcher S, Korgenski EK, Barney T, Daly J, Rogatcheva M. Detection of 23 Gastrointestinal Pathogens Among Children Who Present With Diarrhea. <i>J Ped Infect Dis</i>. 2016; piw020.
Swierczewski BE, Odundo EA, Koech MC, Ndonye JN, Kirera RK, Odhiambo CP, Cheruiyot EK, Shaffer DN, Ombogo AN, Oaks EV. Enteric pathogen surveillance in a case-control study of acute diarrhoea in the town of Kisii, Kenya. <i>J Med Microbiol</i>. 2013; 62(Pt 11): 1774-6.
Tang H, Li Y, Zhang P, Guo J, Huang Z, Xu H, Hou Q, Wang C, Zeng M, Jin H, Hu J, Shi X, Zhang J, Kan B, Xiong Y, Zhao A, Ran L, Xu X. [Surveillance for diarrheagenic Escherichia coli in Shanghai, 2012-2013]. <i>Chin J Epidemiol</i>. 2015; 36(11): 1263-8.
Thompson CN, Phan MVT, Hoang NVM, Minh PV, Vinh NT, Thuy CT, Nga TTT, Rabaa MA, Duy PT, Dung TTN, Phat VV, Nga TVT, Tu LTP, Tuyen HT, Yoshihara K, Jenkins C, Duong VT, Phuc HL, Tuyet PTN, Ngoc NM, Vinh H, Chinh NT, Thuong TC, Tuan HM, Hien TT, Campbell JI, Chau NVV, Thwaites G, Baker S. A prospective multi-center observational study of children hospitalized with diarrhea in Ho Chi Minh City, Vietnam. <i>Am J Trop Med Hyg</i>. 2015; 92(5): 1045–52.
Tobias J, Kassem E, Rubinstein U, Bialik A, Vutukuru S-R, Navaro A, Rokney A, Valinsky L, Ephros M, Cohen D, Muhsen K. Involvement of main diarrheagenic Escherichia coli, with emphasis on enteroaggregative E. coli, in severe non-epidemic pediatric diarrhea in a high-income country. <i>BMC Infect Dis</i>. 2015; 79.
Torres OR, Gonzalez W, Lemus O, Pratdesaba RA, Matute JA, Wiklund G, Sack DA, Bourgeois AL, Svennerholm AM. Toxins and virulence factors of enterotoxigenic Escherichia coli associated with strains isolated from indigenous children and international visitors to a rural community in Guatemala. <i>Epidemiol Infect</i>. 2015; 143(8): 1662-71.
Urrestarazu MI, Liprandi F, Pérez de Suárez E, González R, Pérez-Schael I. [Etiologic, clinical and socio-democratic characteristics of acute diarrhea in Venezuela]. <i>Rev Panam Salud Publica</i>. 1999; 6(3): 149–56.
Varavithya W, Vathanophas K, Bodhidatta L, Punyaratabandhu P, Sangchai R, Athipanyakom S, Wasi C, Echeverria P. Importance of salmonellae and Campylobacter jejuni in the etiology of diarrheal disease among children less than 5 years of age in a community in Bangkok, Thailand. <i>J Clin Microbiol</i>. 1990; 28(11): 2507-10.
Vilchez S, Reyes D, Paniagua M, Bucardo F, Möllby R, Weintraub A. Prevalence of diarrhoeagenic Escherichia coli in children from León, Nicaragua. <i>J Med Microbiol</i>. 2009; 58(Pt 5): 630-7.
Vocale C, Rimoldi SG, Pagani C, Grande R, Pedna F, Arghittu M, Lunghi G, Maraschini A, Gismondo MR, Landini MP, Torresani E, Topin F, Sambri V. Comparative evaluation of the new xTAG GPP multiplex assay in the laboratory diagnosis of acute gastroenteritis. Clinical assessment and potential application from a multicentre Italian study. <i>Int J Infect Dis</i>. 2015; 34: 33-7.
Wierzba TF, Abdel-Messih IA, Abu-Elyazeed R, Putnam SD, Kamal KA, Rozmajzl P, Ahmed SF, Fatah A, Zabedy K, Shaheen HI, Sanders J, Frenck R. Clinic-based surveillance for bacterial- and rotavirus-associated diarrhea in Egyptian children. <i>Am J Trop Med Hyg</i>. 2006; 74(1): 148-53.
Yamashiro T, Nakasone N, Higa N, Iwanaga M, Insisiengmay S, Phounane T, Munnalath K, Sithivong N, Sisavath L, Phanthauamath B, Chomlasak K, Sisulath P, Vongsanith P. Etiological study of diarrheal patients in Vientiane, Lao People's Democratic Republic. <i>J Clin Microbiol</i>. 1998; 36(8): 2195–9.
Yu J, Jing H, Lai S, Xu W, Li M, Wu J, Liu W, Yuan Z, Chen Y, Zhao S, Wang X, Zhao Z, Ran L, Wu S, Klena JD, Feng L, Li F, Ye X, Qiu Y, Wang X, Yu H, Li Z, Yang W. Etiology of diarrhea among children under the age five in China: Results from a five-year surveillance. <i>J Infect</i>. 2015; 71(1): 19-27.

B. *Shigella*

Source
Abdu A, Aboderin AO, Elusiyan JB, Kolawole DO, Lamikanra A. Serogroup distribution of Shigella in Ile-Ife, southwest Nigeria. <i>Trop Gastroenterol</i>. 2013; 34(3): 164â€“9.
Adkins HJ, Escamilla J, Santiago LT, RaÃ±oa C, Echeverria P, Cross JH. Two-year survey of etiologic agents of diarrheal disease at San Lazaro Hospital, Manila, Republic of the Philippines. <i>J Clin Microbiol</i>. 1987; 25(7): 1143-7.
Aga Khan University, Center for Vaccine Development (Chile), Center for Vaccine Development, University of Maryland, Centers for Disease Control and Prevention (CDC), Department of Medical Microbiology and Immunology, GÃ¶teborg University, International Vaccine Institute, Perry Point Cooperative Studies Program Coordinating Center, U.S. Department of Veterans Affairs, School of Medicine, University of Virginia, University of Chile. Pakistan - Karachi Global Enteric Multicenter Study 2011-2013. Baltimore, MD, United States: Center for Vaccine Development, University of Maryland.
Aga Khan University, Fogarty International Center, National Institutes of Health (NIH), Foundation for the National Institutes of Health (FNIH). Pakistan - Naushahro Feroze Malnutrition and Enteric Disease Study 2009-2014.
Aithala G, Al Dhahry SH, Saha A, Elbualy MS. Epidemiological and clinical features of rotavirus gastroenteritis in Oman. <i>J Trop Pediatr</i>. 1996; 42(1): 54-7.
Akhter J, Burdette JM, Qadri SM, Myint SH. Aetiology of gastroenteritis at a major referral centre in Saudi Arabia. <i>J Int Med Res</i>. 1994; 22(1): 47-54.
Akinyemi KO, Oyefolu AO, Opere B, Otunba-Payne VA, Oworu AO. Escherichia coli in patients with acute gastroenteritis in Lagos, Nigeria. <i>East Afr Med J</i>. 1998; 75(9): 512-5.
Al Jarousha AMK, El Jarou MA, El Qouqa IA. Bacterial enteropathogens and risk factors associated with childhood diarrhea. <i>Indian J Pediatr</i>. 2011; 78(2): 165-70.
Albert MJ, Faruque AS, Faruque SM, Sack RB, Mahalanabis D. Case-control study of enteropathogens associated with childhood diarrhea in Dhaka, Bangladesh. <i>J Clin Microbiol</i>. 1999; 37(11): 3458-64.
Al-Gallas N, Bahri O, Bouratbeen A, Ben Haasen A, Ben Aissa R. Etiology of acute diarrhea in children and adults in Tunis, Tunisia, with emphasis on diarrheagenic Escherichia coli: prevalence, phenotyping, and molecular epidemiology. <i>Am J Trop Med Hyg</i>. 2007; 77(3): 571-82.
Ali MB, Ghenghesh KS, Aissa RB, Abuhelfaia A, Dufani M. Etiology of childhood diarrhea in Zliten, Libya. <i>Saudi Med J</i>. 2005; 26(11): 1759â€“65.
Amadi B, Kelly P, Mwiya M, Mulwazi E, Sianongo S, Changwe F, Thomson M, Hachungula J, Watuka A, Walker-Smith J, Chintu C. Intestinal and systemic infection, HIV, and mortality in Zambian children with persistent diarrhea and malnutrition. <i>J Pediatr Gastroenterol Nutr</i>. 2001; 32(5): 550-4.
Anders KL, Thompson CN, Thuy NTV, Nguyet NM, Tu LTP, Dung TTN, Phat VV, Van NTH, Hieu NT, Tham NTH, Ha PTT, Lien LB, Chau NVV, Baker S, Simmons CP. The epidemiology and aetiology of diarrhoeal disease in infancy in southern Vietnam: a birth cohort study. <i>Int J Infect Dis</i>. 2015; 3â€“10.
Anderson EJ, Katz BZ, Polin JA, Reddy S, Weinrobe MH, Noskin GA. Rotavirus in adults requiring hospitalization. <i>J Infect</i>. 2012; 64(1): 89-95.
Arima Y, G. Kaminsky R, Montes Ã • , Adolfo G, Casiano-ColÃ³n A, Guthrie BL, DiGiacomo RF, Jacobs J. Nuevos y viejos agentes asociados a diarrea en niÃ±os en Honduras. <i>Rev Med Hondur</i>. 2011; 79(2): 58â€“64.
Ballard SB, Reaves EJ, Luna CG, Silva ME, Rocha C, Heitzinger K, Saito M, Apaza S, Espetia S, Blazes DL, Tilley DH, Guzman Aguilar RC, Gilman RH, Bausch DG. Epidemiology and Genetic

Characterization of Noroviruses among Adults in an Endemic Setting, Peruvian Amazon Basin, 2004-2011. <i>PLoS One</i>. 2015; 10(7): e0131646.
Baqui AH, Sack RB, Black RE, Haider K, Hossain A, Alim AR, Yunus M, Chowdhury HR, Siddique AK. Enteropathogens associated with acute and persistent diarrhea in Bangladeshi children less than 5 years of age. <i>J Infect Dis</i>. 1992; 166(4): 792â€“6.
Baqui AH, Yunus MD, Zaman K, Mitra AK, Hossain KM. Surveillance of patients attending a rural diarrhoea treatment centre in Bangladesh. <i>Trop Geogr Med</i>. 1991; 43(1-2): 17-22.
Bartlett AV, Torun B, Morales C, Cano F, Cruz JR. Oral gentamicin is not effective treatment for persistent diarrhea. <i>Acta Paediatr Suppl</i>. 1992; 381: 149-54.
Battikhi MNG. Epidemiological study on Jordanian patients suffering from diarrhoea. <i>New Microbiol</i>. 2002; 25(4): 405-12.
Beatty ME, Ochieng JB, Chege W, Kumar L, Okoth G, Shapiro RL, Wells JG, Parsons MB, Bopp C, Chiller T, Vulule J, Mintz E, Slutsker L, Brooks JT. Sporadic paediatric diarrhoeal illness in urban and rural sites in Nyanza Province, Kenya. <i>East Afr Med J</i>. 2009; 86(8): 387-98.
Becker-Dreps S, Bucardo F, Vilchez S, Zambrana LE, Liu L, Weber DJ, PeÃ±a R, Barclay L, VinjÃ© J, Hudgens MG, Nordgren J, Svensson L, Morgan DR, Espinoza F, Paniagua M. Etiology of childhood diarrhea after rotavirus vaccine introduction: a prospective, population-based study in Nicaragua. <i>Pediatr Infect Dis J</i>. 2014; 33(11): 1156â€“63.
Bhan MK, Bhandari N, Sazawal S, Clemens J, Raj P, Levine MM, Kaper JB. Descriptive epidemiology of persistent diarrhoea among young children in rural northern India. <i>Bull World Health Organ</i>. 1989; 67(3): 281-8.
Bhattacharya D, Sugunan AP, Bhattacharjee H, Thamizhmani R, Sayi DS, Thanasekaran K, Manimunda SP, Ghosh AR, Bharadwaj AP, Singhania M, Roy S. Antimicrobial resistance in Shigella--rapid increase & widening of spectrum in Andaman Islands, India. <i>Indian J Med Res</i>. 2012; 365-70.
Black RE, Brown KH, Becker S, Alim AR, Huq I. Longitudinal studies of infectious diseases and physical growth of children in rural Bangladesh. II. Incidence of diarrhea and association with known pathogens. <i>Am J Epidemiol</i>. 1982; 115(3): 315-24.
Black RE, Lopez de RomaÃ±a G, Brown KH, Bravo N, Bazalar OG, Kanashiro HC. Incidence and etiology of infantile diarrhea and major routes of transmission in Huascar, Peru. <i>Am J Epidemiol</i>. 1989; 129(4): 785-99.
Black RE, Merson MH, Huq I, Alim AR, Yunus M. Incidence and severity of rotavirus and Escherichia coli diarrhoea in rural Bangladesh. Implications for vaccine development. <i>Lancet</i>. 1981; 1(8212): 141-3.
Black RE, Merson MH, Rahman AS, Yunus M, Alim AR, Huq I, Yolken RH, Curlin GT. A two-year study of bacterial, viral, and parasitic agents associated with diarrhea in rural Bangladesh. <i>J Infect Dis</i>. 1980; 142(5): 660-4.
Bodhidatta L, Lan NTP, Hien BT, Lai NV, Srijan A, Serichantalergs O, Fukuda CD, Cam PD, Mason CJ. Rotavirus disease in young children from Hanoi, Vietnam. <i>Pediatr Infect Dis J</i>. 2007; 26(4): 325â€“8.
Bodhidatta L, McDaniel P, Sornsakrin S, Srijan A, Serichantalergs O, Mason CJ. Case-control study of diarrheal disease etiology in a remote rural area in Western Thailand. <i>Am J Trop Med Hyg</i>. 2010; 83(5): 1106-9.
Bonkoungou IJO, Haukka K, Ã–sterblad M, Hakanen AJ, TraorÃ© AS, Barro N, Siitonen A. Bacterial and viral etiology of childhood diarrhea in Ouagadougou, Burkina Faso. <i>BMC Pediatr</i>. 2013; 13: 36.
Breurec S, Vanel N, Bata P, Chartier L, Farra A, Favennec L, Franck T, Giles-Vernick T, Gody JC, Luong Nguyen LB, Onambele M, Rafai C, Razakandrainibe R, Tondeur L, Tricou V, Sansonetti P, Vray M. Etiology and Epidemiology of Diarrhea in Hospitalized Children from Low Income Country:

A Matched Case-Control Study in Central African Republic. <i>PLoS Negl Trop Dis</i>. 2016; 10(1): e0004283.
Burke V, Gracey M, Robinson J, Peck D, Beaman J, Bundell C. The microbiology of childhood gastroenteritis: Aeromonas species and other infective agents. <i>J Infect Dis</i>. 1983; 148(1): 68-74.
Cabrita J, Pires I, Vlaes L, Coignau H, Levy J, Goossens H, Goncalves AP, De Mol P, Butzler JP. Campylobacter enteritis in Portugal: epidemiological features and biological markers. <i>Eur J Epidemiol</i>. 1992; 8(1): 22-6.
Cama RI, Parashar UD, Taylor DN, Hickey T, Figueroa D, Ortega YR, Romero S, Perez J, Sterling CR, Gentsch JR, Gilman RH, Glass RI. Enteropathogens and other factors associated with severe disease in children with acute watery diarrhea in Lima, Peru. <i>J Infect Dis</i>. 1999; 179(5): 1139-44.
Carmona RCC, Timenetsky M do CST, da Silva FF, Granato CFH. Characterization of rotavirus strains from hospitalized and outpatient children with acute diarrhoea in SÃ£o Paulo, Brazil. <i>J Med Virol</i>. 2004; 74(1): 166-72.
Casalino M, Yusuf MW, Nicoletti M, Bazzicalupo P, Coppo A, Colonna B, Cappelli C, Bianchini C, Falbo V, Ahmed HJ. A two-year study of enteric infections associated with diarrhoeal diseases in children in urban Somalia. <i>Trans R Soc Trop Med Hyg</i>. 1988; 82(4): 637-41.
Center for Vaccine Development (Chile), Center for Vaccine Development, Ministry of Health (Mali), Center for Vaccine Development, University of Maryland, Centers for Disease Control and Prevention (CDC), Department of Medical Microbiology and Immunology, GÃ¶teborg University, International Vaccine Institute, Perry Point Cooperative Studies Program Coordinating Center, U.S. Department of Veterans Affairs, School of Medicine, University of Virginia, University of Chile. Mali - Bamako Global Enteric Multicenter Study 2011-2013. Baltimore, MD, United States: Center for Vaccine Development, University of Maryland.
Center for Vaccine Development (Chile), Center for Vaccine Development, University of Maryland, Centers for Disease Control and Prevention (CDC), Department of Medical Microbiology and Immunology, GÃ¶teborg University, International Centre for Diarrhoeal Disease Research, Bangladesh (ICDDR,B), International Vaccine Institute, Perry Point Cooperative Studies Program Coordinating Center, U.S. Department of Veterans Affairs, School of Medicine, University of Virginia, University of Chile. Bangladesh - Mizrapur Global Enteric Multicenter Study 2011-2013. Baltimore, MD, United States: Center for Vaccine Development, University of Maryland.
Center for Vaccine Development (Chile), Center for Vaccine Development, University of Maryland, Centers for Disease Control and Prevention (CDC), Department of Medical Microbiology and Immunology, GÃ¶teborg University, International Vaccine Institute, Kenya Medical Research Institute (KEMRI), Perry Point Cooperative Studies Program Coordinating Center, U.S. Department of Veterans Affairs, School of Medicine, University of Virginia, University of Chile. Kenya - Nyanza Global Enteric Multicenter Study 2011-2012. Baltimore, MD, United States: Center for Vaccine Development, University of Maryland.
Center for Vaccine Development (Chile), Center for Vaccine Development, University of Maryland, Centers for Disease Control and Prevention (CDC), Department of Medical Microbiology and Immunology, GÃ¶teborg University, International Vaccine Institute, Manhica Health Research Center (CISM), Perry Point Cooperative Studies Program Coordinating Center, U.S. Department of Veterans Affairs, School of Medicine, University of Virginia, University of Chile. Mozambique - Manhica Global Enteric Multicenter Study 2011-2013. Baltimore, MD, United States: Center for Vaccine Development, University of Maryland.
Center for Vaccine Development (Chile), Center for Vaccine Development, University of Maryland, Centers for Disease Control and Prevention (CDC), Department of Medical Microbiology and Immunology, GÃ¶teborg University, International Vaccine Institute, Medical Research Council (Gambia), Perry Point Cooperative Studies Program Coordinating Center, U.S. Department of Veterans Affairs, School of Medicine, University of Virginia, University of Chile. Gambia - Basse Global

Enteric Multicenter Study 2011-2013. Baltimore, MD, United States: Center for Vaccine Development, University of Maryland.
Center for Vaccine Development (Chile), Center for Vaccine Development, University of Maryland, Centers for Disease Control and Prevention (CDC), Department of Medical Microbiology and Immunology, GÃ¶teborg University, International Vaccine Institute, National Institute of Cholera and Enteric Diseases (India), Perry Point Cooperative Studies Program Coordinating Center, U.S. Department of Veterans Affairs, School of Medicine, University of Virginia, University of Chile. India - Kolkata Global Enteric Multicenter Study 2011-2013. Baltimore, MD, United States: Center for Vaccine Development, University of Maryland.
Chang K, Kim C, Oh S, Lee H, Lee K. A clinical and epidemiological study on rotavirus gastroenteritis in children. <i>J Korean Pediatr Soc</i>. 1988; 961-76.
Chau ML, Hartantyo SHP, Yap M, Kang JSL, Aung KT, GutiÃ©rrez RA, Ng LC, Tam CC, Barkham T. Diarrheagenic pathogens in adults attending a hospital in Singapore. <i>BMC Infect Dis</i>. 2016; 16.
Chen Y, Chen X, Zheng S, Yu F, Kong H, Yang Q, Cui D, Chen N, Lou B, Li X, Tian L, Yang X, Xie G, Dong Y, Qin Z, Han D, Wang Y, Zhang W, Tang YW, Li L. Serotypes, genotypes and antimicrobial resistance patterns of human diarrhoeagenic Escherichia coli isolates circulating in southeastern China. <i>Clin Microbiol Infect</i>. 2014; 20(1): 52-8.
Cheun H-I, Cho S-H, Lee J-H, Lim Y-Y, Jeon J-H, Yu J-R, Kim T-S, Lee W-J, Cho S-H, Lee D-Y, Park M-S, Jeong H-S, Chen D-S, Ji Y-M, Kwon M-H. Infection status of hospitalized diarrheal patients with gastrointestinal protozoa, bacteria, and viruses in the Republic of Korea. <i>Korean J Parasitol</i>. 2010; 48(2): 113-20.
Christian Medical College, Vellore (India), Fogarty International Center, National Institutes of Health (NIH), Foundation for the National Institutes of Health (FNIH). India - Vellore Malnutrition and Enteric Disease Study 2009-2014.
Cover KE, Ruiz SA, Chapman AS. Reported gastrointestinal infections in the U.S. Air Force, 2000-2012. <i>MSMR</i>. 2014; 21(6): 7-Feb.
Cravioto A, Reyes RE, Ortega R, FernÃ¡ndez G, HernÃ¡ndez R, LÃ³pez D. Prospective study of diarrhoeal disease in a cohort of rural Mexican children: incidence and isolated pathogens during the first two years of life. <i>Epidemiol Infect</i>. 1988; 101(1): 123-34.
Cruz JR, CÃ¡ceres P, Cano F, Flores J, Bartlett A, TorÃºn B. Adenovirus types 40 and 41 and rotaviruses associated with diarrhea in children from Guatemala. <i>J Clin Microbiol</i>. 1990; 28(8): 1780-4.
Dagan R, Bar-David Y, Sarov B, Katz M, Kassis I, Greenberg D, Glass RI, Margolis CZ, Sarov I. Rotavirus diarrhea in Jewish and Bedouin children in the Negev region of Israel: epidemiology, clinical aspects and possible role of malnutrition in severity of illness. <i>Pediatr Infect Dis J</i>. 1990; 9(5): 314-21.
Das SK, Ahmed S, Ferdous F, Farzana FD, Chisti MJ, Latham JR, Talukder KA, Rahman M, Begum YA, Qadri F, Faruque AS, Ahmed T. Etiological diversity of diarrhoeal disease in Bangladesh. <i>J Infect Dev Ctries</i>. 2013; 7(12): 900-9.
Das SK, Ahmed S, Ferdous F, Farzana FD, Chisti MJ, Leung DT, Malek MA, Talukder KA, Bardhan PK, Salam MA, Faruque AS, Raqib R. Changing emergence of Shigella sero-groups in Bangladesh: observation from four different diarrheal disease hospitals. <i>PLoS One</i>. 2013; 8(4): e62029.
Denno DM, Shaikh N, Stapp JR, Qin X, Hutter CM, Hoffman V, Mooney JC, Wood KM, Stevens HJ, Jones R, Tarr PI, Klein EJ. Diarrhea etiology in a pediatric emergency department: a case control study. <i>Clin Infect Dis</i>. 2012; 55(7): 897â€“904.
Do TT, Bui TTH, MÃ¸lbak K, Phung DC, Dalsgaard A. Epidemiology and aetiology of diarrhoeal diseases in adults engaged in wastewater-fed agriculture and aquaculture in Hanoi, Vietnam. <i>Trop Med Int Health</i>. 2007; 12(Suppl 2): 23-33.

Echeverria P, Blacklow NR, Cukor GG, Vibulbandhitkit S, Changchawalit S, Boonthai P. Rotavirus as a cause of severe gastroenteritis in adults. <i>J Clin Microbiol</i>. 1983; 18(3): 663-7.
Echeverria P, Hoge CW, Bodhidatta L, Tungtaem C, Herrmann J, Imlarp S, Tamura K. Etiology of diarrhea in a rural community in western Thailand: importance of enteric viruses and enterovirulent Escherichia coli. <i>J Infect Dis</i>. 1994; 169(4): 916-9.
Echeverria P, Seriwatana J, Taylor DN, Yanggratoke S, Tirapat C. A comparative study of enterotoxigenic Escherichia coli, Shigella, Aeromonas, and Vibrio as etiologies of diarrhea in northeastern Thailand. <i>Am J Trop Med Hyg</i>. 1985; 34(3): 547-54.
Echeverria P, Taylor DN, Lexsomboon U, Bhaibulaya M, Blacklow NR, Tamura K, Sakazaki R. Case-control study of endemic diarrheal disease in Thai children. <i>J Infect Dis</i>. 1989; 159(3): 543-8.
El-Mohammady H, Mansour A, Shaheen HI, Henien NH, Motawea MS, Raafat I, Moustafa M, Adib-Messih IA, Sebeny PJ, Young SYN, Klena JD. Increase in the detection rate of viral and parasitic enteric pathogens among Egyptian children with acute diarrhea. <i>J Infect Dev Ctries</i>. 2012; 6(11): 774-81.
el-Sheikh SM, el-Assouli SM. Prevalence of viral, bacterial and parasitic enteropathogens among young children with acute diarrhoea in Jeddah, Saudi Arabia. <i>J Health Popul Nutr</i>. 2001; 19(1): 25â€“30.
Essers B, Burnens AP, Lanfranchini FM, Somaruga SG, von Vigier RO, Schaad UB, Aebi C, Bianchetti MG. Acute community-acquired diarrhea requiring hospital admission in Swiss children. <i>Clin Infect Dis</i>. 2000; 31(1): 192â€“6.
Fang GD, Lima AA, Martins CV, Nataro JP, Guerrant RL. Etiology and epidemiology of persistent diarrhea in northeastern Brazil: a hospital-based, prospective, case-control study. <i>J Pediatr Gastroenterol Nutr</i>. 1995; 21(2): 137-44.
Faruque ASG, Malek MA, Khan AI, Huq S, Salam MA, Sack DA. Diarrhoea in elderly people: aetiology, and clinical characteristics. <i>Scand J Infect Dis</i>. 2004; 36(3): 204-8.
Ferdous F, Ahmed S, Farzana FD, Das J, Malek MA, Das SK, Salam MA, Faruque ASG. Aetiologies of diarrhoea in adults from urban and rural treatment facilities in Bangladesh. <i>Epidemiol Infect</i>. 2015; 143(7): 1377â€“87.
Ferreccio C, Prado V, Ojeda A, Cayyazo M, Abrego P, Guers L, Levine MM. Epidemiologic patterns of acute diarrhea and endemic Shigella infections in children in a poor periurban setting in Santiago, Chile. <i>Am J Epidemiol</i>. 1991; 134(6): 614-27.
Fiedoruk K, Daniluk T, Rozkiewicz D, Zaremba ML, Oldak E, Sciepuk M, Leszczynska K. Conventional and molecular methods in the diagnosis of community-acquired diarrhoea in children under 5 years of age from the north-eastern region of Poland. <i>Int J Infect Dis</i>. 2015; 145â€“51.
Figueroa-Quintanilla D, Salazar-Lindo E, Sack RB, LeÃ³n-BarÃºa R, Sarabia-Arce S, Campos-SÃ¡nchez M, Eyzaguirre-Maccan E. A controlled trial of bismuth subsalicylate in infants with acute watery diarrheal disease. <i>N Engl J Med</i>. 1993; 328(23): 1653-8.
Finamore E, Vitiello M, Kampanaraki A, Rao M, Galdiero M, Galdiero E, Bevilacqua P, Gallo MA, Galdiero M. G2 as an emerging rotavirus strain in pediatric gastroenteritis in southern Italy. <i>Infection</i>. 2011; 39(2): 113-9.
Fogarty International Center, National Institutes of Health (NIH), Foundation for the National Institutes of Health (FNIH), Haydom Lutheran Hospital. Tanzania - Haydom Malnutrition and Enteric Disease Study 2009-2014.
Fogarty International Center, National Institutes of Health (NIH), Foundation for the National Institutes of Health (FNIH), Institute of Medicine, Tribhuvan University, University of Bergen, Walter Reed/AFRIMS Research Unit Nepal (WARUN). Nepal - Bhaktapur Malnutrition and Enteric Disease Study 2009-2014.
Fogarty International Center, National Institutes of Health (NIH), Foundation for the National Institutes of Health (FNIH), International Centre for Diarrhoeal Disease Research, Bangladesh (ICDDR,B). Bangladesh - Dhaka Malnutrition and Enteric Disease Study 2009-2014.

Fogarty International Center, National Institutes of Health (NIH), Foundation for the National Institutes of Health (FNIH), Johns Hopkins Bloomberg School of Public Health. Peru - Loreto Malnutrition and Enteric Disease Study 2009-2014.
Fogarty International Center, National Institutes of Health (NIH), Foundation for the National Institutes of Health (FNIH), National Institute of Science, Technology, and Biomedicine of Semi-Arid Brazil (INCT-IBISAB). Brazil - Fortaleza Malnutrition and Enteric Disease Study 2009-2014.
Fogarty International Center, National Institutes of Health (NIH), Foundation for the National Institutes of Health (FNIH), University of Venda. South Africa - Venda Malnutrition and Enteric Disease Study 2009-2014.
Friesema IH, De Boer RF, Duizer E, Kortbeek LM, Notermans DW, Smeulders A, Bogerman J, Pronk MJ, Uil JJ, Brinkman K, Koopmans MP, Kooistra-Smid AM, Van Duynhoven YT. Aetiology of acute gastroenteritis in adults requiring hospitalization in The Netherlands. <i>Epidemiol Infect</i>. 2012; 140(10): 1780-6.
Gasparinho C, Mirante MC, Centeno-Lima S, Istrate C, Mayer AC, Tavira L, Nery SV, Brito M. Etiology of Diarrhea in Children Younger Than 5 Years Attending the Bengo General Hospital in Angola. <i>Pediatr Infect Dis J</i>. 2016; 35(2): e28-34.
Gassama A, Sow PS, Fall F, Camara P, GuÃ¨ye-N'diaye A, Seng R, Samb B, M'Boup S, AÃ¯dara-Kane A. Ordinary and opportunistic enteropathogens associated with diarrhea in Senegalese adults in relation to human immunodeficiency virus serostatus. <i>Int J Infect Dis</i>. 2001; 5(4): 192-8.
Georges MC, Roure C, Tauxe RV, Meunier DM, Merlin M, Testa J, Baya C, Limbassa J, Georges AJ. DiarrhealÂ morbidity and mortalityÂ inÂ childrenÂ in theÂ Central African Republic. <i>Am J Trop Med Hyg</i>. 1987; 36(3): 598-602.
Georges MC, Wachsmuth IK, Meunier DM, Nebout N, Didier F, Siopathis MR, Georges AJ. Parasitic, bacterial, and viral enteric pathogens associated with diarrhea in the Central African Republic. <i>J Clin Microbiol</i>. 1984; 19(5): 571-5.
Gosselin KB, Aboud S, McDonald CM, Moyo S, Khavari N, Manji K, Kisenge R, Fawzi W, Kellogg M, Tran HQ, Kibiki G, Gratz J, Liu J, Gewirtz A, Houpt E, Duggan C. Etiology of Diarrhea, Nutritional Outcomes and Novel Intestinal Biomarkers in Tanzanian Infants: A Preliminary Study. <i>J Pediatr Gastroenterol Nutr</i>. 2016.
Greenberg BL, Sack RB, Salazar-Lindo E, Budge E, Gutierrez M, Campos M, Visberg A, Leon-Barua R, Yi A, Maurutia D. Measles-associated diarrhea in hospitalized children in Lima, Peru: pathogenic agents and impact on growth. <i>J Infect Dis</i>. 1991; 163(3): 495-502.
Greenhill AR, Guwada C, Siba V, Michael A, Yoannes M, Wawarie Y, Ford R, Siba PM, Horwood PF. Antibiotic resistant Shigella is a major cause of diarrhoea in the Highlands of Papua New Guinea. <i>J Infect Dev Ctries</i>. 2014; 8(11): 1391â€“7.
Guerra-GodÃnez JC, Larrosa-Haro A, Coello-RamÃrez P, Tostado HRA, Rivera-ChÃ¡vez E, Castillo de LeÃ³n YA, BojÃ³rquez-Ramos M del C, Aguilar-Benavides S. Changing trends in prevalence, morbidity, and lethality in persistent diarrhea of infancy during the last decade in Mexico. <i>Arch Med Res</i>. 2003; 34(3): 209-13.
Guerrant RL, Kirchhoff LV, Shields DS, Nations MK, Leslie J, de Sousa MA, Araujo JG, Correia LL, Sauer KT, McClelland KE. Prospective study of diarrheal illnesses in northeastern Brazil: patterns of disease, nutritional impact, etiologies, and risk factors. <i>J Infect Dis</i>. 1983; 148(6): 986-97.
Gupta DN, Sircar BK, Sengupta PG, Ghosh S, Banu MK, Mondal SK, Saha DR, De SP, Sikdar SN, Manna B, Dutta S, Saha NC. Epidemiological and clinical profiles of acute invasive diarrhoea with special reference to mucoid episodes: a rural community-based longitudinal study. <i>Trans R Soc Trop Med Hyg</i>. 1996; 90(5): 544-7.
GusmÃ£o RH, Mascarenhas JD, Gabbay YB, Lins-Lainson Z, Ramos FL, Monteiro TA, Valente SA, Fagundes-Neto U, Linhares AC. Rotavirus subgroups, G serotypes, and electrophoretypes in cases of nosocomial infantile diarrhoea in BelÃ©m, Brazil. <i>J Trop Pediatr</i>. 1999; 45(2): 81-6.

Hall AJ, Rosenthal M, Gregoricus N, Greene SA, Ferguson J, Henao OL, VinjÃ© J, Lopman BA, Parashar UD, Widdowson M-A. Incidence of acute gastroenteritis and role of norovirus, Georgia, USA, 2004-2005. <i>Emerg Infect Dis</i>. 2011; 17(8): 1381-8.
Haque R, Mondal D, Karim A, Molla IH, Rahim A, Faruque ASG, Ahmad N, Kirkpatrick BD, Houpt E, Snider C, Petri WA. Prospective Case-Control Study of the Association between Common Enteric Protozoal Parasites and Diarrhea in Bangladesh. <i>Clin Infect Dis</i>. 2009; 48(9): 1191â€“7.
Haque R, Mondal D, Kirkpatrick BD, Akther S, Farr BM, Sack RB, Petri WA Jr. Epidemiologic and clinical characteristics of acute diarrhea with emphasis on Entamoeba histolytica infections in preschool children in an urban slum of Dhaka, Bangladesh. <i>Am J Trop Med Hyg</i>. 2003; 69(4): 398-405.
Hegazi MA, Patel TA, El-Deek BS. Prevalence and characters of Entamoeba histolytica infection in Saudi infants and children admitted with diarrhea at 2 main hospitals at South Jeddah: a re-emerging serious infection with unusual presentation. <i>Braz J Infect Dis</i>. 2013; 17(1): 32-40.
Henry FJ, Udoy AS, Wanke CA, Aziz KM. Epidemiology of persistent diarrhea and etiologic agents in Mirzapur, Bangladesh. <i>Acta Paediatr Suppl</i>. 1992; 381: 27-31.
Hien BTT, Scheutz F, Cam PD, Serichantalergs O, Huong TT, Thu TM, Dalsgaard A. Diarrheagenic Escherichia coli and Shigella strains isolated from children in a hospital case-control study in Hanoi, Vietnam. <i>J Clin Microbiol</i>. 2008; 46(3): 996â€“1004.
Hossain MA, Albert MJ, Hasan KZ. Epidemiology of shigellosis in Teknaf, a coastal area of Bangladesh: a 10-year survey. <i>Epidemiol Infect</i>. 1990; 105(1): 41-9.
Househam KC, Mann MD, Bowie MD. Enteropathogens associated with acute infantile diarrhoea in Cape Town. <i>S Afr Med J</i>. 1988; 73(2): 83-7.
Howard P, Alexander ND, Atkinson A, Clegg AO, Gerega G, Javati A, Kajoi M, Lupiwa S, Lupiwa T, Mens M, Saleu G, Sanders RC, West B, Alpers MP. Bacterial, viral and parasitic aetiology of paediatric diarrhoea in the highlands of Papua New Guinea. <i>J Trop Pediatr</i>. 2000; 46(1): 10-4.
Hu Q, Lyu D, Shi X, Jiang Y, Lin Y, Li Y, Qiu Y, He L, Zhang R, Li Q. A modified molecular beacons-based multiplex real-time PCR assay for simultaneous detection of eight foodborne pathogens in a single reaction and its application. <i>Foodborne Pathog Dis</i>. 2014; 11(3): 207-14.
Huhulescu S, Kiss R, Brettlecker M, Cerny RJ, Hess C, Wewalka G, Allerberger F. Etiology of acute gastroenteritis in three sentinel general practices, Austria 2007. <i>Infection</i>. 2009; 37(2): 103-8.
Huilan S, Zhen LG, Mathan MM, Mathew MM, Olarte J, Espejo R, Khin Maung U, Ghafoor MA, Khan MA, Sami Z. Etiology of acute diarrhoea among children in developing countries: a multicentre study in five countries. <i>Bull World Health Organ</i>. 1991; 69(5): 549-55.
Islam SS, Shahid NS. Morbidity and mortality in a diarrhoeal diseases hospital in Bangladesh. <i>Trans R Soc Trop Med Hyg</i>. 1986; 80(5): 748-52.
Jafari F, Garcia-Gil LJ, Salmanzadeh-Ahrabi S, Shokrzadeh L, Aslani MM, Pourhoseingholi MA, Derakhshan F, Zali MR. Diagnosis and prevalence of enteropathogenic bacteria in children less than 5 years of age with acute diarrhea in Tehran children's hospitals. <i>J Infect</i>. 2009; 58(1): 21-7.
Jafari F, Shokrzadeh L, Hamidian M, Salmanzadeh-Ahrabi S, Zali MR. Acute diarrhea due to enteropathogenic bacteria in patients at hospitals in Tehran. <i>Jpn J Infect Dis</i>. 2008; 61(4): 269â€“73.
Jain D, Sinha S, Prasad KN, Pandey CM. Campylobacter species and drug resistance in a north Indian rural community. <i>Trans R Soc Trop Med Hyg</i>. 2005; 99(3): 207â€“14.
Jia L, Lin C, Gao Z, Qu M, Yang J, Sun J, Chen H, Wang Q. Prevalence and factors associated with different pathogens of acute diarrhea in adults in Beijing, China. <i>J Infect Dev Ctries</i>. 2016; 10(11): 1200â€“7.
Jiraphongsa C, Bresee JS, Pongsuwanna Y, Kluabwang P, Poonawagul U, Arporntip P, Kanoksil M, Premsri N, Intusoma U. Epidemiology and burden of rotavirus diarrhea in Thailand: results of sentinel surveillance. <i>J Infect Dis</i>. 2005; 192(Suppl 1): S87-93.

Johargy A, Ghazi H, Mumenah A. Frequency of viral, bacterial and parasitic enteropathogens among young children with acute diarrhoea in Saudi Arabia. <i>J Pak Med Assoc</i>. 2010; 60(6): 456â€“9.
Kabayiza J-C, Andersson ME, Nilsson S, BergstrÃ¶m T, Muhirwa G, Lindh M. Real-time PCR identification of agents causing diarrhea in Rwandan children less than 5 years of age. <i>Pediatr Infect Dis J</i>. 2014; 33(10): 1037â€“42.
Kattula D, Francis MR, Kulinkina A, Sarkar R, Mohan VR, Babji S, Ward HD, Kang G, Balraj V, Naumova EN. Environmental predictors of diarrhoeal infection for rural and urban communities in south India in children and adults. <i>Epidemiol Infect</i>. 2015; 143(14): 3036-47.
Khan AM, Hossain MS, Khan AI, Chisti MJ, Chowdhury F, Faruque ASG, Salam MA. Bacterial enteropathogens of neonates admitted to an urban diarrhoeal hospital in Bangladesh. <i>J Trop Pediatr</i>. 2009; 55(2): 122-4.
Klein EJ, Boster DR, Stapp JR, Wells JG, Qin X, Clausen CR, Swerdlow DL, Braden CR, Tarr PI. Diarrhea etiology in a Children's Hospital Emergency Department: a prospective cohort study. <i>Clin Infect Dis</i>. 2006; 43(7): 807-13.
Kotloff KL, Nataro JP, Blackwelder WC, Nasrin D, Farag TH, Panchalingam S, Wu Y, Sow SO, Sur D, Breiman RF, Faruque AS, Zaidi AK, Saha D, Alonso PL, Tamboura B, Sanogo D, Onwuchekwa U, Manna B, Ramamurthy T, Kanungo S, Ochieng JB, Omore R, Oundo JO, Hossain A, Das SK, Ahmed S, Qureshi S, Quadri F, Adegbola RA, Antonio M, Hossain MJ, Akinsola A, Mandomando I, Nhampossa T, AcÃ¡cio S, Biswas K, Oâ€™Reilly CE, Mintz ED, Berkeley LY, Muhsen K, Sommerfelt H, Robins-Browne RM, Levine MM. Burden and aetiology of diarrhoeal disease in infants and young children in developing countries (the Global Enteric Multicenter Study, GEMS): a prospective, case-control study. <i>Lancet</i>. 2013; 382(9888): 209â€“22.
Kownhar H, Shankar EM, Rajan R, Vengatesan A, Rao UA. Prevalence of Campylobacter jejuni and enteric bacterial pathogens among hospitalized HIV infected versus non-HIV infected patients with diarrhoea in southern India. <i>Scand J Infect Dis</i>. 2007; 39(10): 862â€“6.
Krumkamp R, Sarpong N, Schwarz NG, Adlkofer J, Adelkofer J, Loag W, Eibach D, Hagen RM, Adu-Sarkodie Y, Tannich E, May J. Gastrointestinal infections and diarrheal disease in Ghanaian infants and children: an outpatient case-control study. <i>PLoS Negl Trop Dis</i>. 2015; 9(3): e0003568.
Kulkarni S, Patsute S, Sane S, Chandane M, Vidhate P, Risbud A. Enteric pathogens in HIV infected and HIV uninfected individuals with diarrhea in Pune. <i>Trans R Soc Trop Med Hyg</i>. 2013; 107(10): 648â€“52.
Kullin B, Meggersee R, Dâ€™Alton J, Galvao B, Rajabally N, Whitelaw A, Bamford C, Reid SJ, Abratt VR. Prevalence of gastrointestinal pathogenic bacteria in patients with diarrhoea attending Groote Schuur Hospital, Cape Town, South Africa. <i>S Afr Med J</i>. 2015; 105(2): 121â€“5.
Lanata CF, Black RE, MaÃºrtua D, Gil A, Gabilondo A, Yi A, Miranda E, Gilman RH, LeÃ³n-BarÃºa R, Sack RB. Etiologic agents in acute vs persistent diarrhea in children under three years of age in peri-urban Lima, Per. <i>Acta Paediatr Suppl</i>. 1992; 32-8.
Langendorf C, Le Hello S, Moumouni A, Gouali M, Mamaty A-A, Grais RF, Weill F-X, Page A-L. Enteric bacterial pathogens in children with diarrhea in Niger: diversity and antimicrobial resistance. <i>PLoS One</i>. 2015; 10(3): e0120275.
Leung DT, Das SK, Malek MA, Qadri F, Faruque ASG, Ryan ET. Impact of Ramadan on clinical and microbiologic parameters of patients seen at a diarrheal hospital in urban Dhaka, Bangladesh, 1996-2012. <i>Am J Trop Med Hyg</i>. 2014; 90(2): 294â€“8.
Levidiotou S, Gartzonika C, Papaventsis D, Christaki C, Priavali E, Zotos N, Kapsali E, Vrioni G. Viral agents of acute gastroenteritis in hospitalized children in Greece. <i>Clin Microbiol Infect</i>. 2009; 15(6): 596-8.
Li J, Hu J, Wu H, Pan H, Zhang X, Xue Y, Wu F. [Etiological surveillance for diarrhea in Shanghai from August 2013 to July 2014]. <i>Chin J Epidemiol</i>. 2015; 36(10): 1099-103.

Li Y, Xie X, Xu X, Wang X, Chang H, Wang C, Wang A, He Y, Yu H, Wang X, Zeng M. Nontyphoidal salmonella infection in children with acute gastroenteritis: prevalence, serotypes, and antimicrobial resistance in Shanghai, China. <i>Foodborne Pathog Dis</i>. 2014; 11(3): 200-6.

Lim YS, Tay L. A one-year study of enteric Campylobacter infections in Singapore. <i>J Trop Med Hyg</i>. 1992; 95(2): 119-23.

Lima AA, Moore SR, Barboza MS Jr, Soares AM, Schleupner MA, Newman RD, Sears CL, Nataro JP, Fedorko DP, Wuhib T, Schorling JB, Guerrant RL. Persistent diarrhea signals a critical period of increased diarrhea burdens and nutritional shortfalls: a prospective cohort study among children in northeastern Brazil. <i>J Infect Dis</i>. 2000; 181(5): 1643-51.

Linhares AC, MonÃ§Ã£o HC, Gabbay YB, de AraÃºjo VL, Serruya AC, Loureiro EC. Acute diarrhoea associated with rotavirus among children living in BelÃ©m, Brazil. <i>Trans R Soc Trop Med Hyg</i>. 1983; 77(3): 384-90.

Loening WE, Coovadia YM, Van den Ende J. Aetiological factors of infantile diarrhoea: a community-based study. <i>Ann Trop Paediatr</i>. 1989; 9(4): 248-55.

Lozer DM, Souza TB, Monfardini MV, Vicentini F, Kitagawa SS, Scaletsky IC, Spano LC. Genotypic and phenotypic analysis of diarrheagenic Escherichia coli strains isolated from Brazilian children living in low socioeconomic level communities. <i>BMC Infect Dis</i>. 2013; 418.

MÃ¸lbak K, Wested N, HÃ¸jlyng N, Scheutz F, Gottschau A, Aaby P, da Silva AP. The etiology of early childhood diarrhea: a community study from Guinea-Bissau. <i>J Infect Dis</i>. 1994; 169(3): 581-7.

Manrique-Abril FG, Tigne y Diane B, Bello SE, Ospina JM. [Diarrhoea-causing agents in children aged less than five in Tunja, Colombia]. <i>Rev Salud Publica (Bogota)</i>. 2006; 8(1): 88â€“97.

Mansour A, Shaheen HI, Amine M, Hassan K, Sanders JW, Riddle MS, Armstrong AW, Svennerholm AM, Sebeny PJ, Klena JD, Young SY, Frenck RW. Pathogenicity and phenotypic characterization of enterotoxigenic Escherichia coli isolates from a birth cohort of children in rural Egypt. <i>J Clin Microbiol</i>. 2014; 52(2): 587-91.

Maraki S, Georgiladakis A, Tselentis Y, Samonis G. A 5-year study of the bacterial pathogens associated with acute diarrhoea on the island of Crete, Greece, and their resistance to antibiotics. <i>Eur J Epidemiol</i>. 2003; 18(1): 85â€“90.

Mathan VI, Rajan DP. The prevalence of bacterial intestinal pathogens in a healthy rural population in southern India. <i>J Med Microbiol</i>. 1986; 22(2): 93-6.

Mathur R, Reddy V, Naidu AN, Ravikumar, Krishnamachari KA. Nutritional status and diarrhoeal morbidity: a longitudinal study in rural Indian preschool children . <i>Hum Nutr Clin Nutr</i>. 1985; 39(6): 447-54.

Meng CY, Smith BL, Bodhidatta L, Richard SA, Vansith K, Thy B, Srijan A, Serichantalergs O, Mason CJ. Etiology of diarrhea in young children and patterns of antibiotic resistance in Cambodia. <i>Pediatr Infect Dis J</i>. 2011; 30(4): 331-5.

Mishra OP, Dhawan T, Singla PN, Dixit VK, Arya NC, Nath G. Endoscopic and histopathological evaluation of preschool children with chronic diarrhoea. <i>J Trop Pediatr</i>. 2001; 47(2): 77-80.

MoezArdalan K, Zali MR, Dallal MMS, Hemami MR, Salmanzadeh-Ahrabi S. Prevalence and pattern of antimicrobial resistance of Shigella species among patients with acute diarrhoea in Karaj, Tehran, Iran. <i>J Health Popul Nutr</i>. 2003; 21(2): 96-102.

Muhsen K, Shulman L, Rubinstein U, Kasem E, Kremer A, Goren S, Zilberstein I, Chodick G, Ephros M, Cohen D. Incidence, characteristics, and economic burden of rotavirus gastroenteritis associated with hospitalization of israeli children <5 years of age, 2007-2008. <i>J Infect Dis</i>. 2009; 200(Suppl 1): S254-263.

Mulatu G, Beyene G, Zeynudin A. Prevalence of Shigella, Salmonella and Campylobacter species and their susceptibility patters among under five children with diarrhea in Hawassa town, south Ethiopia. <i>Ethiop J Health Sci</i>. 2014; 24(2): 101â€“8.

Mutanda LN, Kangethe SK, Juma R, Lichenga EO, Gathecha C. Aetiology of diarrhoea in malnourished children at Kenyatta National Hospital. <i>East Afr Med J</i>. 1985; 62(12): 835-41.
Mutanda LN. Epidemiology of acute gastroenteritis in early childhood in Kenya. III. Distribution of the aetiological agents. <i>East Afr Med J</i>. 1980; 57(5): 317-26.
Mutanda LN. Epidemiology of acute gastroenteritis in early childhood in Kenya: aetiological agents. <i>Trop Geogr Med</i>. 1980; 32(2): 138-44.
Nair GB, Ramamurthy T, Bhattacharya MK, Krishnan T, Ganguly S, Saha DR, Rajendran K, Manna B, Ghosh M, Okamoto K, Takeda Y. Emerging trends in the etiology of enteric pathogens as evidenced from an active surveillance of hospitalized diarrhoeal patients in Kolkata, India. <i>Gut Pathog</i>. 2010; 2(1): 4.
Nakano T, Kamiya H, Matsubayashi N, Watanabe M, Sakurai M, Honda T. Diagnosis of bacterial enteric infections in children in Zambia. <i>Acta Paediatr Jpn</i>. 1998; 40(3): 259â€“63.
Nath G, Choudhury A, Shukla BN, Singh TB, Reddy DC. Significance of Cryptosporidium in acute diarrhoea in North-Eastern India. <i>J Med Microbiol</i>. 1999; 48(6): 523-6.
Nhampossa T, Mandomando I, Acacio S, Quinto L, Vubil D, Ruiz J, Nhalungo D, Sacoor C, Nhabanga A, Nhacolo A, Aide P, Machevo S, Sigauque B, Nhama A, Kotloff K, Farag T, Nasrin D, Bassat Q, Macete E, Levine MM, Alonso P. Diarrheal Disease in Rural Mozambique: Burden, Risk Factors and Etiology of Diarrheal Disease among Children Aged 0-59 Months Seeking Care at Health Facilities. <i>PLoS One</i>. 2015; 10(5): e0119824.
Norwegian Institute of Public Health. Norway Cause of Death Registry 1982.
Nunes MRCM, MagalhÃ£es PP, Penna FJ, Nunes JMM, Mendes EN. Diarrhea associated with Shigella in children and susceptibility to antimicrobials. <i>J Pediatr (Rio J)</i>. 2012; 88(2): 125-8.
Oberhelman RA, Gilman RH, Sheen P, Taylor DN, Black RE, Cabrera L, Lescano AG, Meza R, Madico G. A placebo-controlled trial of Lactobacillus GG to prevent diarrhea in undernourished Peruvian children. <i>J Pediatr</i>. 1999; 134(1): 15-20.
Oberle MW, Merson MH, Islam MS, Rahman AS, Huber DH, Curlin G. Diarrhoeal disease in Bangladesh: epidemiology, mortality averted and costs at a rural treatment centre. <i>Int J Epidemiol</i>. 1980; 9(4): 341-8.
Obi CL, Coker AO, Epoke J, Ndip RN. Enteric bacterial pathogens in stools of residents of urban and rural regions in Nigeria: a comparison of patients with and without diarrhoea and controls without diarrhoea. <i>J Diarrhoeal Dis Res</i>. 1997; 15(4): 241â€“7.
Ochoa TJ, Ecker L, Barletta F, Mispireta ML, Gil AI, Contreras C, Molina M, Amemiya I, Verastegui H, Hall ER, Cleary TG, Lanata CF. Age-related susceptibility to infection with diarrheagenic Escherichia coli among infants from Periurban areas in Lima, Peru. <i>Clin Infect Dis</i>. 2009; 49(11): 1694-702.
O'Reilly CE, Jaron P, Ochieng B, Nyaguara A, Tate JE, Parsons MB, Bopp CA, Williams KA, VinjÃ© J, Blanton E, Wannemuehler KA, Vulule J, Laserson KF, Breiman RF, Feikin DR, Widdowson M-A, Mintz E. Risk factors for death among children less than 5 years old hospitalized with diarrhea in rural western Kenya, 2005-2007: a cohort study. <i>PLoS Med</i>. 2012; 9(7): e1001256.
Paniagua GL, Monroy E, GarcÃa-GonzÃ¡lez O, Alonso J, Negrete E, Vaca S. Two or more enteropathogens are associated with diarrhoea in Mexican children. <i>Ann Clin Microbiol Antimicrob</i>. 2007; 6: 17.
Parashar UD, Holman RC, Clarke MJ, Bresee JS, Glass RI. Hospitalizations associated with rotavirus diarrhea in the United States, 1993 through 1995: surveillance based on the new ICD-9-CM rotavirus-specific diagnostic code. <i>J Infect Dis</i>. 1998; 177(1): 13-7.
Patzi-Vargas S, Zaidi MB, Perez-Martinez I, LeÃ³n-Cen M, Michel-Ayala A, Chaussabel D, Estrada-Garcia T. Diarrheagenic Escherichia coli carrying supplementary virulence genes are an important cause of moderate to severe diarrhoeal disease in Mexico. <i>PLoS Negl Trop Dis</i>. 2015; 9(3): e0003510.

Pazzaglia G, Bourgeois AL, Araby I, Mikhail I, Podgore JK, Mourad A, Riad S, Gaffar T, Ramadan AM. Campylobacter-associated diarrhoea in Egyptian infants: epidemiology and clinical manifestations of disease and high frequency of concomitant infections. <i>J Diarrhoeal Dis Res</i>. 1993; 11(1): 6-13.

Pazzaglia G, Sack RB, Salazar E, Yi A, Chea E, Leon-Barua R, Guerrero CE, Palomino J. High frequency of coinfecting enteropathogens in Aeromonas-associated diarrhea of hospitalized Peruvian infants. <i>J Clin Microbiol</i>. 1991; 29(6): 1151-6.

Penny ME, Paredes P, Brown KH, Laughan B, Smith H. Lack of a role of the duodenal microflora in pathogenesis of persistent diarrhea and diarrhea-related malabsorption in Peruvian children. <i>Pediatr Infect Dis J</i>. 1990; 9(7): 479-87.

Platts-Mills JA, Gratz J, Mduma E, Svensen E, Amour C, Liu J, Maro A, Saidi Q, Swai N, Kumburu H, McCormick BJ, Kibiki G, Houpt ER. Association between stool enteropathogen quantity and disease in Tanzanian children using TaqMan array cards: a nested case-control study. <i>Am J Trop Med Hyg</i>. 2014; 90(1): 133-8.

Poocharoen L, Bruin CW, Sirisanthana V, Vannareumol P, Leechanachai P, Sukhavat K. The relative importance of various enteropathogens as a cause of diarrhoea in hospitalized children in Chiang Mai, Thailand. <i>J Diarrhoeal Dis Res</i>. 1986; 4(1): 10-5.

Punyaratabandhu P, Vathanophas K, Varavithya W, Sangchai R, Athipanyakom S, Echeverria P, Wasi C. Childhood diarrhoea in a low-income urban community in Bangkok: incidence, clinical features, and child caretaker's behaviours. <i>J Diarrhoeal Dis Res</i>. 1991; 9(3): 244-9.

Qadri F, Saha A, Ahmed T, Al Tarique A, Begum YA, Svennerholm AM. Disease burden due to enterotoxigenic Escherichia coli in the first 2 years of life in an urban community in Bangladesh. <i>Infect Immun</i>. 2007; 75(8): 3961-8.

Qu M, Deng Y, Zhang X, Liu G, Huang Y, Lin C, Li J, Yan H, Li X, Jia L, Kan B, Huang F, Wang Q. Etiology of acute diarrhea due to enteropathogenic bacteria in Beijing, China. <i>J Infect</i>. 2012; 65(3): 214-22.

Rahouma A, Klena JD, Krema Z, Abobker AA, Treesh K, Franka E, Abusnena O, Shaheen HI, El Mohammady H, Abudher A, Ghenghesh KS. Enteric pathogens associated with childhood diarrhea in Tripoli-Libya. <i>Am J Trop Med Hyg</i>. 2011; 84(6): 886â€“91.

Reither K, Ignatius R, Weitzel T, Seidu-Korkor A, Anyidoho L, Saad E, Djie-Maletz A, Ziniel P, Amoo-Sakyi F, Danikuu F, Danour S, Otchwemah RN, Schreier E, Bienzle U, Stark K, Mockenhaupt FP. Acute childhood diarrhoea in northern Ghana: epidemiological, clinical and microbiological characteristics. <i>BMC Infect Dis</i>. 2007; 7: 104.

SÃ¡nchez-Capilla AD, SorlÃ³zano-Puerto A, RodrÃguez-Granger J, MartÃnez-Brocal A, Navarro-MarÃ JM, GutiÃ©rrez-FernÃ¡ndez J. Infectious etiology of diarrheas studied in a third-level hospital during a five-year period. <i>Rev Esp Enferm Dig</i>. 2015; 107(2): 89â€“97.

Saeed A, Abd H, Sandstrom G. Microbial aetiology of acute diarrhoea in children under five years of age in Khartoum, Sudan. <i>J Med Microbiol</i>. 2015; 64(Pt 4): 432â€“7.

Saha MR, Sen D, Datta P, Datta D, Pal SC. Role of rotavirus as the cause of acute paediatric diarrhoea in Calcutta. <i>Trans R Soc Trop Med Hyg</i>. 1984; 78(6): 818-20.

Saidi SM, Iijima Y, Sang WK, Mwangudza AK, Oundo JO, Taga K, Aihara M, Nagayama K, Yamamoto H, Waiyaki PG, Honda T. Epidemiological study on infectious diarrheal diseases in children in a coastal rural area of Kenya. <i>Microbiol Immunol</i>. 1997; 41(10): 773-8.

Samal SK, Khuntia HK, Nanda PK, Satapathy CS, Nayak SR, Sarangi AK, Sahoo N, Pattnaik SK, Chhotray GP, Pal BB. Incidence of bacterial enteropathogens among hospitalized diarrhea patients from Orissa, India. <i>Jpn J Infect Dis</i>. 2008; 61(5): 350-5.

Sambe-Ba B, Espie E, Faye ME, Timbin? LG, Sembene M, Gassama-Sow A. Community-acquired diarrhea among children and adults in urban settings in Senegal: clinical, epidemiological and microbiological aspects. <i>BMC Infect Dis</i>. 2013; 580.

Samie A, Guerrant RL, Barrett L, Bessong PO, Igumbor EO, Obi CL. Prevalence of intestinal parasitic and bacterial pathogens in diarrhoeal and non-diarroeal human stools from Vhembe district, South Africa. <i>J Health Popul Nutr</i>. 2009; 27(6): 739-45.
Samonis G, Maraki S, Christidou A, Georgiladakis A, Tselentis Y. Bacterial pathogens associated with diarrhoea on the island of Crete. <i>Eur J Epidemiol</i>. 1997; 13(7): 831-6.
Sarkar SR, Hossain MA, Paul SK, Ray NC, Monwar S. Rotavirus is Predominent Enteropathogen in Acute Childhood Diarrhea in Mymensingh. <i>Mymensingh Med J</i>. 2015; 24(4): 665-70.
Schorling JB, Wanke CA, Schorling SK, McAuliffe JF, De Souza MA, Guerrant RL. A prospective study of persistent diarrhea among children in an urban Brazilian slum. Patterns of occurrence and etiologic agents. <i>Am J Epidemiol</i>. 1990; 132(1): 144-56.
Shahid NS, Greenough WB 3rd, Samadi AR, Huq MI, Rahman N. Hand washing with soap reduces diarrhoea and spread of bacterial pathogens in a Bangladesh village. <i>J Diarrhoeal Dis Res</i>. 1996; 14(2): 85-9.
Sherchand JB, Tandukar S, Sherchan JB, Rayamajhi A, Gurung B, Shrestha L, Rijal B, Pokhrel BM. Hospital-based study in children with rotavirus gastroenteritis and other enteropathogens. <i>J Nepal Health Res Counc</i>. 2012; 10(21): 130-5.
Shukry S, Zaki AM, DuPont HL, Shoukry I, el Tagi M, Hamed Z. Detection of enteropathogens in fatal and potentially fatal diarrhea in Cairo, Egypt. <i>J Clin Microbiol</i>. 1986; 24(6): 959-62.
Simpore J, Ouermi D, Ilboudo D, Kabre A, Zeba B, Pietra V, Pignatelli S, Nikiema JB, Kabre GB, Caligaris S, Schumacher F, Castelli F. Aetiology of acute gastro-enteritis in children at Saint Camille Medical Centre, Ouagadougou, Burkina Faso. <i>Pak J Biol Sci</i>. 2009; 12(3): 258-63.
Sire J-M, Garin B, Chartier L, Fall NK, Tall A, Seck A, Weill F-X, Breurec S, Vray M. Community-acquired infectious diarrhoea in children under 5 years of age in Dakar, Senegal. <i>Paediatr Int Child Health</i>. 2013; 33(3): 139â€"44.
Sobel J, Gomes TAT, Ramos RTS, Hoekstra M, Rodrigue D, Rassi V, Griffin PM. Pathogen-specific risk factors and protective factors for acute diarrheal illness in children aged 12-59 months in SÃ£o Paulo, Brazil. <i>Clin Infect Dis</i>. 2004; 38(11): 1545-51.
Soli KW, Maure T, Kas MP, Bande G, Bebes S, Luang-Suarkia D, Siba PM, Morita A, Umezaki M, Greenhill AR, Horwood PF. Detection of enteric viral and bacterial pathogens associated with paediatric diarrhoea in Goroka, Papua New Guinea. <i>Int J Infect Dis</i>. 2014; 27: 54-58.
Sousa MÅ', Mendes EN, Collares GB, P?ret-Filho LA, Penna FJ, MagalhÃœes PP. Shigella in Brazilian children with acute diarrhoea: prevalence, antimicrobial resistance and virulence genes. <i>Mem Inst Oswaldo Cruz</i>. 2013; 108(1): 30-5.
Steenland MW, Joseph GA, Lucien MA, Freeman N, Hast M, Nygren BL, Leshem E, Juin S, Parsons MB, Talkington DF, Mintz ED, Vertefeuille J, Balajee SA, Boncy J, Katz MA. Laboratory-confirmed cholera and rotavirus among patients with acute diarrhea in four hospitals in Haiti, 2012-2013. <i>Am J Trop Med Hyg</i>. 2013; 89(4): 641-6.
Stockmann C, Pavia AT, Graham B, Vaughn M, Crisp R, Poritz MA, Thatcher S, Korgenski EK, Barney T, Daly J, Rogatcheva M. Detection of 23 Gastrointestinal Pathogens Among Children Who Present With Diarrhea. <i>J Pediatr Infect Dis Soc</i>. 2016; piw020.
Stoll BJ, Glass RI, Huq MI, Khan MU, Holt JE, Banu H. Surveillance of patients attending a diarrhoeal disease hospital in Bangladesh. <i>Br Med J (Clin Res Ed)</i>. 1982; 285(6349): 1185-8.
Sutra S, Kosuwon P, Chirawatkul A, Thepsuthammarat K. Burden of acute, persistent and chronic diarrhea, Thailand, 2010. <i>J Med Assoc Thai</i>. 2012; S97-107.
Svanteson B, ThorÃ©n A, Castor B, Barkenius G, Bergdahl U, Tufvesson B, Hansson HB, MÃ¶llby R, Juhlin I. Acute diarrhoea in adults: aetiology, clinical appearance and therapeutic aspects. <i>Scand J Infect Dis</i>. 1988; 20(3): 303-14.
Swierczewski BE, Odundo EA, Koech MC, Ndonye JN, Kirera RK, Odhiambo CP, Cheruiyot EK, Shaffer DN, Ombogo AN, Oaks EV. Enteric pathogen surveillance in a case-control study of acute diarrhoea in the town of Kisii, Kenya. <i>J Med Microbiol</i>. 2013; 62(Pt 11): 1774-6.

Tam CC, O'Brien SJ, Tompkins DS, Bolton FJ, Berry L, Dodds J, Choudhury D, Halstead F, Iturriza-GÃ‘mara M, Mather K, Rait G, Ridge A, Rodrigues LC, Wain J, Wood B, Gray JJ. Changes in causes of acute gastroenteritis in the United Kingdom over 15 years: microbiologic findings from 2 prospective, population-based studies of infectious intestinal disease. <i>Clin Infect Dis</i>. 2012; 54(9): 1275-86.

Tanaka G, Faruque ASG, Luby SP, Malek MA, Glass RI, Parashar UD. Deaths from rotavirus disease in Bangladeshi children: estimates from hospital-based surveillance. <i>Pediatr Infect Dis J</i>. 2007; 26(11): 1014-8.

Taniuchi M, Sobuz SU, Begum S, Platts-Mills JA, Liu J, Yang Z, Wang XQ, Petri WA, Haque R, Houpt ER. Etiology of diarrhea in Bangladeshi infants in the first year of life analyzed using molecular methods. <i>J Infect Dis</i>. 2013; 208(11): 1794-802.

Thompson CN, Phan MVT, Hoang NVM, Minh PV, Vinh NT, Thuy CT, Nga TTT, Rabaa MA, Duy PT, Dung TTN, Phat VV, Nga TVT, Tu LTP, Tuyen HT, Yoshihara K, Jenkins C, Duong VT, Phuc HL, Tuyet PTN, Ngoc NM, Vinh H, Chinh NT, Thuong TC, Tuan HM, Hien TT, Campbell JI, Chau NVV, Thwaites G, Baker S. A prospective multi-center observational study of children hospitalized with diarrhea in Ho Chi Minh City, Vietnam. <i>Am J Trop Med Hyg</i>. 2015; 92(5): 1045â€“52.

Tobias J, Kassem E, Rubinstein U, Bialik A, Vutukuru S-R, Navaro A, Rokney A, Valinsky L, Ephros M, Cohen D, Muhsen K. Involvement of main diarrheagenic Escherichia coli, with emphasis on enteroaggregative E. coli, in severe non-epidemic pediatric diarrhea in a high-income country. <i>BMC Infect Dis</i>. 2015; 79.

Torres OR, Gonzalez W, Lemus O, Pratdesaba RA, Matute JA, Wiklund G, Sack DA, Bourgeois AL, Svennerholm AM. Toxins and virulence factors of enterotoxigenic Escherichia coli associated with strains isolated from indigenous children and international visitors to a rural community in Guatemala. <i>Epidemiol Infect</i>. 2015; 143(8): 1662-71.

Urbina D, Arzuza O, Young G, Parra E, Castro R, Puello M. Rotavirus type A and other enteric pathogens in stool samples from children with acute diarrhea on the Colombian northern coast. <i>Int Microbiol</i>. 2003; 6(1): 27â€“32.

Urrestarazu MI, Liprandi F, PÃ©rez de SuÃ¡rez E, GonzÃ¡lez R, PÃ©rez-Schael I. [Etiologic, clinical and socio-democratic characteristics of acute diarrhea in Venezuela]. <i>Rev Panam Salud Publica</i>. 1999; 6(3): 149â€“56.

Varavithya W, Vathanophas K, Bodhidatta L, Punyaratabandhu P, Sangchai R, Athipanyakom S, Wasi C, Echeverria P. Importance of salmonellae and Campylobacter jejuni in the etiology of diarrheal disease among children less than 5 years of age in a community in Bangkok, Thailand. <i>J Clin Microbiol</i>. 1990; 28(11): 2507-10.

Vocale C, Rimoldi SG, Pagani C, Grande R, Pedna F, Arghittu M, Lunghi G, Maraschini A, Gismondo MR, Landini MP, Torresani E, Topin F, Sambri V. Comparative evaluation of the new xTAG GPP multiplex assay in the laboratory diagnosis of acute gastroenteritis. Clinical assessment and potential application from a multicentre Italian study. <i>Int J Infect Dis</i>. 2015; 34: 33-7.

Von Seidlein L, Kim DR, Ali M, Lee H, Wang X, Thiem VD, Canh DG, Chaicumpa W, Agtini MD, Hossain A, Bhutta ZA, Mason C, Sethabutr O, Talukder K, Nair GB, Deen JL, Kotloff K, Clemens J. A Multicentre Study of Shigella Diarrhoea in Six Asian Countries: Disease Burden, Clinical Manifestations, and Microbiology. <i>PLoS Med</i>. 2006; 3(9): e353.

Vrbova L, Johnson K, Whitfield Y, Middleton D. A descriptive study of reportable gastrointestinal illnesses in Ontario, Canada, from 2007 to 2009. <i>BMC Public Health</i>. 2012; 973.

Watson B, Ellis M, Mandal B, Dunbar E, Whale K, Brennand J. A comparison of the clinico-pathological features with stool pathogens in patients hospitalised with the symptom of diarrhoea. <i>J Infect Dis</i>. 1986; 18(6): 553-9.

Wierzba TF, Abdel-Messih IA, Abu-Elyazeed R, Putnam SD, Kamal KA, Rozmajzl P, Ahmed SF, Fatah A, Zabedy K, Shaheen HI, Sanders J, Frenck R. Clinic-based surveillance for bacterial- and rotavirus-associated diarrhea in Egyptian children. <i>Am J Trop Med Hyg</i>. 2006; 74(1): 148-53.

Workman SN, Sobers SJ, Mathison GE, Lavoie MC. Human Campylobacter-associated enteritis on the Caribbean island of Barbados. <i>Am J Trop Med Hyg</i>. 2006; 74(4): 623â€“7.
Wu J. Diarrheal diseases in rural Bangladesh: spatial-temporal patterns, risk factors and pathogen detection. <i>Diss Abstr Int</i>. 2011; 73(1).
Xue Y, Pan H, Hu J, Wu H, Li J, Xiao W, Zhang X, Yuan Z, Wu F. Epidemiology of norovirus infections among diarrhea outpatients in a diarrhea surveillance system in Shanghai, China: a cross-sectional study. <i>BMC Infect Dis</i>. 2015; 15: 183.
Yamashiro T, Nakasone N, Higa N, Iwanaga M, Insisiengmay S, Phounane T, Munnalath K, Sithivong N, Sisavath L, Phanthauamath B, Chomlasak K, Sisulath P, Vongsanith P. Etiological study of diarrheal patients in Vientiane, Lao Peopleâ€™s Democratic Republic. <i>J Clin Microbiol</i>. 1998; 36(8): 2195â€“9.
Yu J, Jing H, Lai S, Xu W, Li M, Wu J, Liu W, Yuan Z, Chen Y, Zhao S, Wang X, Zhao Z, Ran L, Wu S, Klena JD, Feng L, Li F, Ye X, Qiu Y, Wang X, Yu H, Li Z, Yang W. Etiology of diarrhea among children under the age five in China: Results from a five-year surveillance. <i>J Infect</i>. 2015; 71(1): 19-27.
Zaki AM, DuPont HL, el Alamy MA, Arafat RR, Amin K, Awad MM, Bassiouni L, Imam IZ, el Malih GS, el Marsafie A. The detection of enteropathogens in acute diarrhea in a family cohort population in rural Egypt. <i>Am J Trop Med Hyg</i>. 1986; 35(5): 1013-22.
Zhang H, Pan F, Zhao X, Wang G, Tu Y, Fu S, Wang J, Pan J, Song J, Wang W, Jin Z, Xu H, Ren Y, Li Y, Zhong N. Distribution and antimicrobial resistance of enteric pathogens in Chinese paediatric diarrhoea: a multicentre retrospective study, 2008-2013. <i>Epidemiol Infect</i>. 2015; 143(12): 2512-9.
Zhang Y, Zhao Y, Ding K, Wang X, Chen X, Liu Y, Chen Y. Analysis of bacterial pathogens causing acute diarrhea on the basis of sentinel surveillance in Shanghai, China, 2006-2011. <i>Jpn J Infect Dis</i>. 2014; 67(4): 264-8.
Zhu X-H, Tian L, Cheng Z-J, Liu W-Y, Li S, Yu W-T, Zhang W-Q, Xiang X, Sun Z-Y. Viral and Bacterial Etiology of Acute Diarrhea among Children under 5 Years of Age in Wuhan, China. <i>Chin Med J (Engl)</i>. 2016; 129(16): 1939â€“44.
Zielinski A, Czarkowski MP, SadkowskaTodys M. Infectious diseases in Poland in 2012. <i>Przegl Epidemiol</i>. 2014; 68(2): 177-85.

Supplementary Table 3. Estimated country-level results for Shigella diarrhoea in 2016

Values in parentheses are 95% uncertainty intervals.

	Under-5				All ages			
Location	Deaths	Deaths per 100,000	Incidence per 1000	Cases	Deaths	Deaths per 100,000	Incidence per 1000	Cases
Global	63,713 (41,191 to 93,611)	10.1 (6.5 to 14.8)	116.2 (64.3 to 198.6)	74,771,591 (41,395,286 to 127,742,524)	212,438 (136,979 to 326,913)	2.9 (1.9 to 4.4)	36.4 (23.9 to 49.8)	269,191,131 (176,677,465 to 368,995,635)
High-income	34 (20 to 52)	0.1 (0.0 to 0.1)	13.1 (6.1 to 25.3)	759,474 (353,998 to 1,471,183)	1,128 (687 to 1,638)	0.1 (0.1 to 0.2)	2.8 (1.7 to 4.1)	2,921,208 (1,812,321 to 4,298,658)
High-income North America	13 (7 to 20)	0.1 (0.0 to 0.1)	7.0 (2.4 to 13.9)	149,441 (52,237 to 299,100)	666 (444 to 902)	0.2 (0.1 to 0.3)	3.6 (2.2 to 5.1)	1,284,736 (804,562 to 1,820,497)
Canada	0 (0 to 1)	0.0 (0.0 to 0.0)	4.3 (1.4 to 9.3)	8,438 (2,735 to 18,047)	95 (61 to 131)	0.3 (0.2 to 0.4)	2.3 (1.4 to 3.3)	83,544 (50,354 to 120,913)
Greenland	0 (0 to 0)	0.1 (0.0 to 0.1)	5.6 (1.8 to 12.6)	19 (6 to 43)	0 (0 to 0)	0.1 (0.0 to 0.2)	2.9 (1.7 to 4.3)	143 (84 to 212)
United States	12 (7 to 20)	0.1 (0.0 to 0.1)	7.2 (2.6 to 14.4)	140,978 (50,092 to 281,133)	572 (379 to 776)	0.2 (0.1 to 0.2)	3.7 (2.3 to 5.3)	1,201,050 (750,764 to 1,695,793)
Australasia	0 (0 to 1)	0.0 (0.0 to 0.0)	1.7 (0.5 to 3.9)	3,168 (870 to 7,035)	14 (8 to 20)	0.0 (0.0 to 0.1)	1.3 (0.7 to 2.0)	36,416 (18,800 to 57,873)
Australia	0 (0 to 1)	0.0 (0.0 to 0.0)	1.5 (0.4 to 3.3)	2,243 (615 to 5,042)	11 (7 to 16)	0.0 (0.0 to 0.1)	1.1 (0.6 to 1.8)	26,756 (13,672 to 42,853)
New Zealand	0 (0 to 0)	0.0 (0.0 to 0.1)	3.2 (0.9 to 7.1)	923 (258 to 2,085)	3 (2 to 4)	0.1 (0.0 to 0.1)	2.1 (1.1 to 3.4)	9,658 (4,981 to 15,454)
High-income Asia Pacific	0 (0 to 1)	0.0 (0.0 to 0.0)	0.2 (0.1 to 1.1)	1,778 (717 to 8,373)	35 (6 to 74)	0.0 (0.0 to 0.0)	0.1 (0.0 to 0.2)	11,608 (5,854 to 34,659)
Brunei	0 (0 to 0)	0.0 (0.0 to 0.0)	0.2 (0.1 to 1.1)	8 (3 to 39)	0 (0 to 0)	0.0 (0.0 to 0.0)	0.1 (0.0 to 0.3)	33 (13 to 115)
Japan	0 (0 to 1)	0.0 (0.0 to 0.0)	0.3 (0.1 to 1.3)	1,414 (500 to 6,767)	29 (5 to 57)	0.0 (0.0 to 0.0)	0.1 (0.0 to 0.2)	9,352 (4,588 to 29,077)
Singapore	0 (0 to 0)	0.0 (0.0 to 0.0)	0.4 (0.1 to 1.9)	68 (14 to 331)	0 (0 to 1)	0.0 (0.0 to 0.0)	0.1 (0.0 to 0.3)	408 (120 to 1,340)
South Korea	0 (0 to 0)	0.0 (0.0 to 0.0)	0.1 (0.1 to 0.5)	288 (137 to 1,117)	6 (1 to 17)	0.0 (0.0 to 0.0)	0.0 (0.0 to 0.1)	1,815 (1,049 to 4,668)
Western Europe	3 (1 to 5)	0.0 (0.0 to 0.0)	4.4 (0.7 to 14.2)	97,088 (14,470 to 315,796)	253 (74 to 468)	0.1 (0.0 to 0.1)	0.7 (0.2 to 1.7)	305,157 (85,895 to 732,647)

Location	Under-5				All ages			
	Deaths	Deaths per 100,000	Incidence per 1000	Cases	Deaths	Deaths per 100,000	Incidence per 1000	Cases
Andorra	0 (0 to 0)	0.0 (0.0 to 0.0)	4.7 (0.4 to 17.9)	14 (1 to 54)	0 (0 to 0)	0.0 (0.0 to 0.1)	0.6 (0.1 to 1.8)	49 (9 to 138)
Austria	0 (0 to 0)	0.0 (0.0 to 0.0)	14.3 (1.2 to 38.8)	5,638 (460 to 15,293)	2 (1 to 4)	0.0 (0.0 to 0.0)	2.4 (0.9 to 4.6)	21,181 (7,480 to 39,524)
Belgium	0 (0 to 0)	0.0 (0.0 to 0.0)	5.9 (0.5 to 22.1)	3,655 (331 to 13,747)	11 (2 to 23)	0.1 (0.0 to 0.2)	0.9 (0.2 to 2.4)	9,885 (1,755 to 27,733)
Cyprus	0 (0 to 0)	0.0 (0.0 to 0.1)	5.7 (0.5 to 22.0)	296 (28 to 1,135)	0 (0 to 1)	0.1 (0.0 to 0.1)	0.9 (0.1 to 2.5)	799 (136 to 2,290)
Denmark	0 (0 to 0)	0.0 (0.0 to 0.0)	5.2 (0.5 to 19.7)	1,499 (143 to 5,625)	5 (1 to 11)	0.1 (0.0 to 0.2)	0.8 (0.1 to 2.3)	4,694 (850 to 13,092)
Finland	0 (0 to 0)	0.0 (0.0 to 0.0)	7.3 (0.7 to 27.4)	2,140 (195 to 8,000)	1 (0 to 2)	0.0 (0.0 to 0.0)	1.1 (0.2 to 3.1)	6,045 (1,057 to 17,217)
France	1 (0 to 2)	0.0 (0.0 to 0.0)	3.9 (0.4 to 15.3)	15,347 (1,423 to 59,869)	52 (9 to 106)	0.1 (0.0 to 0.2)	0.7 (0.1 to 1.9)	43,565 (7,737 to 122,615)
Germany	0 (0 to 1)	0.0 (0.0 to 0.0)	5.6 (0.5 to 21.2)	19,565 (1,809 to 73,810)	81 (14 to 165)	0.1 (0.0 to 0.2)	0.8 (0.2 to 2.2)	63,509 (12,352 to 178,529)
Greece	0 (0 to 0)	0.0 (0.0 to 0.0)	0.6 (0.4 to 0.8)	278 (169 to 400)	0 (0 to 0)	0.0 (0.0 to 0.0)	0.1 (0.1 to 0.1)	1,176 (814 to 1,446)
Iceland	0 (0 to 0)	0.0 (0.0 to 0.0)	5.1 (0.5 to 19.1)	108 (10 to 409)	0 (0 to 0)	0.0 (0.0 to 0.0)	0.8 (0.1 to 2.5)	281 (46 to 816)
Ireland	0 (0 to 0)	0.0 (0.0 to 0.0)	4.4 (0.4 to 16.9)	1,635 (147 to 6,302)	1 (0 to 1)	0.0 (0.0 to 0.0)	0.9 (0.1 to 2.5)	4,007 (619 to 11,677)
Israel	0 (0 to 1)	0.0 (0.0 to 0.1)	9.7 (3.3 to 21.8)	8,174 (2,786 to 18,336)	14 (8 to 20)	0.2 (0.1 to 0.2)	2.7 (1.3 to 4.5)	21,768 (10,983 to 36,633)
Italy	0 (0 to 0)	0.0 (0.0 to 0.0)	2.0 (0.2 to 7.7)	5,495 (494 to 20,840)	10 (4 to 18)	0.0 (0.0 to 0.0)	0.3 (0.1 to 0.8)	18,759 (3,459 to 50,142)
Luxembourg	0 (0 to 0)	0.0 (0.0 to 0.0)	5.2 (0.5 to 19.3)	151 (14 to 562)	0 (0 to 1)	0.1 (0.0 to 0.1)	0.8 (0.1 to 2.1)	434 (76 to 1,233)
Malta	0 (0 to 0)	0.0 (0.0 to 0.0)	1.9 (0.2 to 7.4)	40 (4 to 156)	0 (0 to 0)	0.0 (0.0 to 0.0)	0.3 (0.1 to 0.8)	118 (21 to 338)
Netherlands	0 (0 to 0)	0.0 (0.0 to 0.0)	12.5 (0.7 to 37.4)	10,866 (634 to 32,495)	19 (9 to 30)	0.1 (0.1 to 0.2)	2.4 (0.7 to 4.8)	40,986 (11,211 to 83,075)
Norway	0 (0 to 0)	0.0 (0.0 to 0.0)	4.3 (0.4 to 16.4)	1,221 (115 to 4,644)	5 (1 to 11)	0.1 (0.0 to 0.2)	0.6 (0.1 to 1.8)	3,341 (596 to 9,448)
Portugal	0 (0 to 0)	0.0 (0.0 to 0.1)	7.4 (1.7 to 17.1)	3,441 (799 to 7,905)	7 (4 to 10)	0.1 (0.0 to 0.1)	1.4 (0.6 to 2.3)	14,355 (6,568 to 24,029)
Spain	0 (0 to 0)	0.0 (0.0 to 0.0)	0.4 (0.3 to 0.6)	1,004 (612 to 1,452)	1 (1 to 3)	0.0 (0.0 to 0.0)	0.1 (0.1 to 0.1)	4,267 (2,936 to 5,273)

Location	Under-5				All ages			
	Deaths	Deaths per 100,000	Incidence per 1000	Cases	Deaths	Deaths per 100,000	Incidence per 1000	Cases
Sweden	0 (0 to 0)	0.0 (0.0 to 0.0)	2.8 (0.4 to 11.8)	1,571 (238 to 6,683)	8 (2 to 15)	0.1 (0.0 to 0.1)	0.5 (0.1 to 1.4)	4,516 (1,297 to 14,055)
Switzerland	0 (0 to 0)	0.0 (0.0 to 0.0)	1.0 (0.5 to 4.7)	428 (192 to 1,959)	1 (0 to 2)	0.0 (0.0 to 0.0)	0.2 (0.1 to 0.6)	1,592 (890 to 4,733)
England	0 (0 to 1)	0.0 (0.0 to 0.0)	3.7 (0.4 to 12.8)	11,788 (1,186 to 40,801)	28 (12 to 48)	0.1 (0.0 to 0.1)	0.6 (0.1 to 1.6)	31,325 (6,311 to 85,872)
Northern Ireland	0 (0 to 0)	0.0 (0.0 to 0.1)	4.4 (0.4 to 16.6)	523 (48 to 1,974)	1 (0 to 1)	0.0 (0.0 to 0.1)	0.7 (0.1 to 2.0)	1,284 (212 to 3,709)
Scotland	0 (0 to 0)	0.0 (0.0 to 0.0)	4.3 (0.4 to 16.6)	1,173 (108 to 4,515)	3 (0 to 5)	0.0 (0.0 to 0.1)	0.6 (0.1 to 1.7)	3,305 (586 to 9,378)
Wales	0 (0 to 0)	0.0 (0.0 to 0.1)	6.9 (0.6 to 26.1)	1,112 (101 to 4,201)	2 (0 to 4)	0.1 (0.0 to 0.1)	1.0 (0.2 to 2.9)	3,164 (558 to 9,081)
Southern Latin America	18 (11 to 26)	0.4 (0.2 to 0.5)	102.0 (51.7 to 172.5)	510,676 (258,860 to 863,584)	160 (111 to 212)	0.2 (0.2 to 0.3)	18.9 (12.2 to 26.8)	1,236,711 (793,600 to 1,748,503)
Argentina	16 (10 to 24)	0.4 (0.3 to 0.7)	115.6 (58.6 to 195.5)	409,923 (207,851 to 693,190)	94 (65 to 125)	0.2 (0.1 to 0.3)	21.8 (13.9 to 31.1)	952,416 (606,966 to 1,356,309)
Chile	1 (1 to 2)	0.1 (0.1 to 0.2)	75.2 (38.8 to 128.1)	91,814 (47,339 to 156,448)	47 (30 to 66)	0.3 (0.2 to 0.4)	14.4 (9.4 to 19.9)	262,400 (171,803 to 362,256)
Uruguay	1 (0 to 2)	0.4 (0.2 to 0.7)	37.3 (19.3 to 66.9)	8,913 (4,611 to 15,966)	19 (13 to 26)	0.6 (0.4 to 0.8)	6.3 (4.0 to 9.2)	21,766 (13,940 to 31,545)
Central Europe, Eastern Europe, and Central Asia	58 (28 to 101)	0.2 (0.1 to 0.4)	17.0 (2.7 to 39.9)	515,950 (81,413 to 1,214,374)	99 (57 to 154)	0.0 (0.0 to 0.0)	3.2 (0.7 to 6.6)	1,316,738 (312,834 to 2,758,538)
Eastern Europe	5 (3 to 9)	0.0 (0.0 to 0.1)	18.0 (3.1 to 42.5)	246,225 (42,744 to 582,156)	16 (9 to 24)	0.0 (0.0 to 0.0)	3.0 (0.7 to 6.3)	637,552 (158,281 to 1,326,809)
Belarus	0 (0 to 0)	0.0 (0.0 to 0.0)	17.7 (2.7 to 44.0)	10,559 (1,619 to 26,265)	0 (0 to 1)	0.0 (0.0 to 0.0)	2.7 (0.7 to 5.7)	26,140 (6,382 to 54,904)
Estonia	0 (0 to 0)	0.0 (0.0 to 0.0)	37.0 (6.2 to 81.8)	2,843 (473 to 6,284)	0 (0 to 0)	0.0 (0.0 to 0.0)	6.4 (1.7 to 13.0)	8,449 (2,260 to 17,243)
Latvia	0 (0 to 0)	0.0 (0.0 to 0.0)	28.3 (4.5 to 68.1)	3,661 (579 to 8,799)	0 (0 to 0)	0.0 (0.0 to 0.0)	4.6 (1.1 to 9.6)	9,189 (2,197 to 19,308)
Lithuania	0 (0 to 0)	0.0 (0.0 to 0.0)	34.0 (5.3 to 81.0)	6,002 (936 to 14,307)	0 (0 to 0)	0.0 (0.0 to 0.0)	5.3 (1.3 to 11.0)	15,397 (3,773 to 32,235)
Moldova	0 (0 to 0)	0.1 (0.0 to 0.1)	15.1 (2.5 to 37.7)	3,368 (548 to 8,403)	0 (0 to 0)	0.0 (0.0 to 0.0)	2.1 (0.5 to 4.3)	8,349 (2,022 to 17,650)

Location	Under-5 Deaths	Under-5 Deaths per 100,000	Under-5 Incidence per 1000	Under-5 Cases	All ages Deaths	All ages Deaths per 100,000	All ages Incidence per 1000	All ages Cases
Russia	5 (2 to 8)	0.0 (0.0 to 0.1)	16.6 (3.0 to 39.2)	164,166 (29,173 to 387,112)	13 (7 to 20)	0.0 (0.0 to 0.0)	2.9 (0.7 to 6.0)	419,403 (104,858 to 872,275)
Ukraine	1 (0 to 1)	0.0 (0.0 to 0.1)	21.4 (3.4 to 52.0)	55,637 (8,728 to 135,205)	2 (1 to 3)	0.0 (0.0 to 0.0)	3.3 (0.8 to 7.0)	150,597 (35,722 to 322,124)
Central Europe	2 (1 to 3)	0.0 (0.0 to 0.1)	24.7 (3.3 to 59.9)	141,247 (19,115 to 341,975)	21 (12 to 32)	0.0 (0.0 to 0.0)	3.2 (0.7 to 6.7)	371,186 (85,778 to 780,121)
Albania	0 (0 to 0)	0.0 (0.0 to 0.0)	19.4 (2.5 to 49.8)	3,367 (427 to 8,639)	0 (0 to 0)	0.0 (0.0 to 0.0)	2.6 (0.6 to 5.8)	7,515 (1,620 to 16,701)
Bosnia and Herzegovina	0 (0 to 0)	0.0 (0.0 to 0.1)	16.8 (2.1 to 42.1)	2,899 (368 to 7,272)	0 (0 to 0)	0.0 (0.0 to 0.0)	2.1 (0.5 to 4.6)	8,061 (1,849 to 17,409)
Bulgaria	0 (0 to 0)	0.1 (0.0 to 0.1)	21.5 (2.7 to 54.4)	7,321 (920 to 18,478)	1 (0 to 1)	0.0 (0.0 to 0.0)	2.6 (0.6 to 5.7)	18,919 (4,256 to 41,416)
Croatia	0 (0 to 0)	0.0 (0.0 to 0.0)	28.5 (3.8 to 64.9)	5,663 (762 to 12,911)	1 (0 to 1)	0.0 (0.0 to 0.0)	3.3 (0.7 to 7.0)	13,942 (3,116 to 29,359)
Czech Republic	0 (0 to 0)	0.0 (0.0 to 0.0)	25.2 (3.3 to 61.0)	14,267 (1,879 to 34,554)	5 (3 to 8)	0.1 (0.0 to 0.1)	3.7 (0.9 to 8.0)	39,832 (9,197 to 84,777)
Hungary	0 (0 to 0)	0.0 (0.0 to 0.1)	21.1 (2.7 to 53.3)	10,733 (1,397 to 27,146)	6 (3 to 8)	0.1 (0.0 to 0.1)	3.3 (0.7 to 7.0)	32,607 (7,263 to 69,837)
Macedonia	0 (0 to 0)	0.1 (0.0 to 0.2)	18.0 (2.4 to 44.3)	2,051 (274 to 5,046)	0 (0 to 0)	0.0 (0.0 to 0.0)	2.5 (0.6 to 5.3)	5,115 (1,179 to 10,983)
Montenegro	0 (0 to 0)	0.0 (0.0 to 0.0)	12.3 (1.6 to 30.9)	455 (58 to 1,142)	0 (0 to 0)	0.0 (0.0 to 0.0)	1.9 (0.4 to 4.0)	1,171 (268 to 2,514)
Poland	0 (0 to 0)	0.0 (0.0 to 0.0)	25.5 (3.2 to 65.0)	50,746 (6,387 to 129,155)	5 (3 to 7)	0.0 (0.0 to 0.0)	3.4 (0.8 to 7.2)	130,347 (29,555 to 278,394)
Romania	1 (0 to 2)	0.1 (0.0 to 0.2)	28.6 (3.7 to 71.4)	22,819 (2,945 to 56,923)	2 (1 to 3)	0.0 (0.0 to 0.0)	3.3 (0.8 to 7.0)	62,747 (14,630 to 135,342)
Serbia	0 (0 to 0)	0.0 (0.0 to 0.1)	22.8 (3.1 to 52.0)	9,348 (1,290 to 21,330)	1 (0 to 2)	0.0 (0.0 to 0.0)	2.7 (0.6 to 5.6)	23,548 (5,480 to 49,426)
Slovakia	0 (0 to 0)	0.0 (0.0 to 0.1)	28.1 (3.6 to 70.3)	8,368 (1,085 to 20,931)	1 (0 to 1)	0.0 (0.0 to 0.0)	3.6 (0.8 to 7.8)	19,846 (4,363 to 42,834)
Slovenia	0 (0 to 0)	0.0 (0.0 to 0.0)	30.2 (3.9 to 73.9)	3,212 (412 to 7,858)	0 (0 to 0)	0.0 (0.0 to 0.0)	3.6 (0.8 to 7.8)	7,465 (1,619 to 16,115)
Central Asia	51 (24 to 87)	0.5 (0.3 to 0.9)	11.7 (1.9 to 28.5)	128,492 (21,248 to 314,213)	62 (32 to 104)	0.1 (0.0 to 0.1)	3.4 (0.9 to 7.2)	307,312 (76,571 to 645,813)

Location	Under-5 Deaths	Under-5 Deaths per 100,000	Under-5 Incidence per 1000	Under-5 Cases	All ages Deaths	All ages Deaths per 100,000	All ages Incidence per 1000	All ages Cases
Armenia	0 (0 to 0)	0.1 (0.0 to 0.2)	16.5 (2.6 to 40.5)	3,707 (586 to 9,098)	0 (0 to 1)	0.0 (0.0 to 0.0)	3.5 (0.9 to 7.5)	10,774 (2,723 to 22,964)
Azerbaijan	5 (2 to 10)	0.5 (0.2 to 1.0)	18.3 (3.0 to 45.3)	16,976 (2,790 to 41,977)	6 (3 to 11)	0.1 (0.0 to 0.1)	4.3 (1.1 to 9.1)	42,453 (10,326 to 89,648)
Georgia	0 (0 to 1)	0.1 (0.0 to 0.2)	16.9 (2.7 to 42.2)	4,724 (749 to 11,798)	1 (0 to 1)	0.0 (0.0 to 0.0)	2.9 (0.7 to 6.2)	11,625 (2,825 to 24,603)
Kazakhstan	2 (1 to 3)	0.1 (0.0 to 0.2)	11.4 (1.8 to 27.9)	22,358 (3,531 to 54,415)	2 (1 to 4)	0.0 (0.0 to 0.0)	3.2 (0.8 to 6.6)	56,384 (13,826 to 117,721)
Kyrgyzstan	6 (3 to 10)	0.8 (0.4 to 1.3)	11.3 (1.9 to 27.3)	9,188 (1,536 to 22,126)	6 (3 to 11)	0.1 (0.1 to 0.2)	3.6 (0.9 to 7.4)	21,571 (5,402 to 44,994)
Mongolia	0 (0 to 0)	0.0 (0.0 to 0.0)	16.4 (2.7 to 38.8)	6,102 (1,007 to 14,428)	0 (0 to 0)	0.0 (0.0 to 0.0)	4.1 (1.0 to 8.6)	12,401 (2,931 to 26,246)
Tajikistan	30 (12 to 59)	2.7 (1.1 to 5.2)	20.2 (3.3 to 49.1)	22,191 (3,577 to 53,971)	37 (17 to 67)	0.4 (0.2 to 0.8)	5.5 (1.3 to 11.8)	47,070 (11,361 to 101,210)
Turkmenistan	3 (1 to 6)	0.6 (0.2 to 1.1)	21.8 (3.5 to 53.4)	11,669 (1,893 to 28,606)	4 (2 to 7)	0.1 (0.0 to 0.1)	5.5 (1.4 to 11.5)	29,910 (7,625 to 62,561)
Uzbekistan	4 (2 to 8)	0.1 (0.0 to 0.2)	6.6 (1.1 to 16.5)	31,662 (5,075 to 79,329)	5 (3 to 10)	0.0 (0.0 to 0.0)	2.4 (0.6 to 5.0)	75,067 (18,623 to 158,495)
Latin America and Caribbean	858 (534 to 1,297)	1.7 (1.1 to 2.6)	174.7 (96.5 to 290.0)	8,260,053 (4,564,295 to 13,715,403)	2,406 (1,702 to 3,230)	0.4 (0.3 to 0.6)	41.5 (26.5 to 58.5)	23,810,923 (15,211,811 to 33,589,462)
Central Latin America	543 (339 to 818)	2.4 (1.5 to 3.6)	149.7 (79.0 to 272.8)	3,345,795 (1,766,692 to 6,097,394)	1,448 (1,033 to 1,948)	0.6 (0.4 to 0.8)	39.0 (24.1 to 57.0)	9,911,117 (6,129,519 to 14,485,425)
Colombia	27 (14 to 48)	0.8 (0.4 to 1.3)	125.1 (51.4 to 266.3)	404,970 (166,453 to 861,700)	79 (52 to 110)	0.2 (0.1 to 0.2)	32.5 (19.3 to 48.4)	1,564,541 (928,741 to 2,331,193)
Costa Rica	2 (1 to 3)	0.5 (0.2 to 1.0)	172.7 (73.8 to 360.3)	56,012 (23,954 to 116,870)	11 (7 to 16)	0.2 (0.1 to 0.3)	48.6 (28.3 to 76.5)	234,909 (137,069 to 370,119)
El Salvador	9 (4 to 18)	1.7 (0.7 to 3.4)	216.6 (92.2 to 450.1)	101,576 (43,231 to 211,055)	35 (18 to 68)	0.6 (0.3 to 1.1)	54.8 (32.2 to 87.6)	334,442 (196,484 to 534,606)
Guatemala	183 (110 to 287)	9.3 (5.6 to 14.6)	306.7 (173.1 to 498.8)	587,216 (331,516 to 955,095)	445 (296 to 621)	2.7 (1.8 to 3.8)	81.0 (51.2 to 117.5)	1,334,799 (843,055 to 1,934,679)

Location	Under-5 Deaths	Under-5 Deaths per 100,000	Under-5 Incidence per 1000	Under-5 Cases	All ages Deaths	All ages Deaths per 100,000	All ages Incidence per 1000	All ages Cases
Honduras	40 (15 to 77)	4.2 (1.6 to 8.2)	217.0 (85.4 to 425.9)	203,361 (79,981 to 399,076)	119 (61 to 212)	1.4 (0.7 to 2.5)	54.8 (29.9 to 91.1)	455,729 (248,803 to 757,476)
Mexico	175 (113 to 259)	1.5 (1.0 to 2.2)	77.8 (39.4 to 139.5)	907,658 (459,966 to 1,626,987)	511 (364 to 668)	0.4 (0.3 to 0.5)	17.5 (11.1 to 25.3)	2,251,706 (1,432,650 to 3,255,247)
Nicaragua	7 (3 to 13)	1.2 (0.5 to 2.2)	57.9 (19.1 to 122.6)	35,276 (11,617 to 74,745)	14 (7 to 24)	0.2 (0.1 to 0.4)	16.4 (7.8 to 27.9)	101,096 (48,058 to 172,028)
Panama	12 (6 to 22)	3.5 (1.7 to 6.3)	201.9 (88.0 to 408.9)	73,443 (32,010 to 148,715)	24 (15 to 36)	0.6 (0.4 to 0.9)	56.5 (32.6 to 91.2)	225,107 (130,080 to 363,520)
Venezuela	88 (48 to 147)	3.0 (1.7 to 5.1)	344.2 (172.3 to 633.6)	974,233 (487,602 to 1,793,302)	210 (143 to 296)	0.7 (0.5 to 0.9)	108.0 (67.0 to 159.4)	3,391,191 (2,103,961 to 5,003,717)
Andean Latin America	49 (29 to 77)	0.7 (0.4 to 1.2)	69.6 (33.7 to 121.4)	456,738 (221,153 to 796,428)	123 (77 to 206)	0.2 (0.1 to 0.3)	23.5 (13.8 to 34.9)	1,403,049 (827,826 to 2,085,319)
Bolivia	21 (10 to 38)	1.5 (0.7 to 2.7)	82.9 (37.8 to 154.3)	110,725 (50,404 to 205,979)	47 (27 to 79)	0.4 (0.2 to 0.7)	28.4 (16.4 to 43.5)	313,387 (180,466 to 480,002)
Ecuador	11 (6 to 18)	0.6 (0.3 to 1.0)	75.5 (35.1 to 134.3)	127,584 (59,285 to 227,066)	23 (15 to 33)	0.1 (0.1 to 0.2)	23.2 (13.6 to 34.7)	381,475 (224,121 to 570,943)
Peru	18 (9 to 29)	0.5 (0.3 to 0.8)	61.8 (30.1 to 106.1)	218,388 (106,374 to 374,921)	53 (31 to 103)	0.2 (0.1 to 0.3)	21.9 (12.9 to 32.4)	708,101 (417,384 to 1,044,654)
Caribbean	85 (34 to 175)	2.1 (0.8 to 4.4)	14.7 (1.7 to 44.3)	60,519 (7,022 to 182,851)	152 (72 to 266)	0.3 (0.2 to 0.6)	3.7 (1.0 to 8.8)	169,689 (46,754 to 401,393)
Antigua and Barbuda	0 (0 to 0)	0.1 (0.0 to 0.2)	8.4 (1.3 to 37.3)	39 (6 to 172)	0 (0 to 0)	0.0 (0.0 to 0.0)	1.8 (0.5 to 5.7)	162 (44 to 518)
The Bahamas	0 (0 to 0)	0.1 (0.0 to 0.2)	7.2 (1.1 to 32.1)	230 (34 to 1,024)	0 (0 to 0)	0.0 (0.0 to 0.0)	1.9 (0.5 to 6.0)	741 (195 to 2,372)
Barbados	0 (0 to 0)	0.0 (0.0 to 0.1)	1.7 (1.0 to 5.4)	27 (15 to 84)	0 (0 to 0)	0.0 (0.0 to 0.0)	0.7 (0.4 to 1.1)	189 (126 to 301)
Belize	0 (0 to 0)	0.2 (0.0 to 0.4)	7.2 (1.2 to 31.4)	297 (48 to 1,298)	0 (0 to 0)	0.0 (0.0 to 0.1)	2.1 (0.5 to 7.1)	778 (187 to 2,610)
Bermuda	0 (0 to 0)	0.0 (0.0 to 0.0)	8.0 (1.1 to 35.5)	35 (5 to 157)	0 (0 to 0)	0.0 (0.0 to 0.0)	1.9 (0.5 to 6.3)	137 (36 to 442)

Location	Under-5 Deaths	Under-5 Deaths per 100,000	Under-5 Incidence per 1000	Under-5 Cases	All ages Deaths	All ages Deaths per 100,000	All ages Incidence per 1000	All ages Cases
Cuba	0 (0 to 0)	0.0 (0.0 to 0.1)	4.5 (0.7 to 20.1)	2,788 (424 to 12,417)	4 (1 to 7)	0.0 (0.0 to 0.1)	1.3 (0.4 to 3.9)	14,342 (4,359 to 44,776)
Dominica	0 (0 to 0)	0.1 (0.0 to 0.3)	6.8 (1.0 to 30.7)	37 (6 to 166)	0 (0 to 0)	0.0 (0.0 to 0.0)	1.9 (0.5 to 5.9)	139 (37 to 438)
Dominican Republic	4 (1 to 8)	0.4 (0.1 to 0.9)	8.1 (1.2 to 35.4)	8,196 (1,245 to 36,039)	7 (2 to 15)	0.1 (0.0 to 0.1)	2.1 (0.5 to 6.8)	22,444 (5,593 to 72,575)
Grenada	0 (0 to 0)	0.1 (0.0 to 0.2)	6.9 (1.0 to 31.3)	83 (12 to 373)	0 (0 to 0)	0.0 (0.0 to 0.0)	2.8 (0.7 to 9.1)	304 (77 to 1,000)
Guyana	0 (0 to 1)	0.4 (0.1 to 1.1)	5.1 (0.8 to 21.9)	362 (59 to 1,560)	1 (0 to 2)	0.1 (0.0 to 0.2)	1.6 (0.4 to 5.1)	1,206 (322 to 3,912)
Haiti	80 (33 to 166)	5.3 (2.1 to 10.9)	29.5 (2.6 to 77.7)	44,584 (3,936 to 117,514)	136 (65 to 242)	1.2 (0.6 to 2.2)	10.2 (2.6 to 21.3)	113,582 (28,829 to 237,063)
Jamaica	0 (0 to 1)	0.1 (0.0 to 0.3)	4.4 (0.7 to 18.9)	1,173 (182 to 5,060)	1 (0 to 3)	0.0 (0.0 to 0.1)	1.2 (0.3 to 3.8)	3,460 (928 to 10,913)
Puerto Rico	0 (0 to 0)	0.0 (0.0 to 0.1)	5.6 (0.8 to 25.6)	1,351 (197 to 6,159)	1 (0 to 2)	0.0 (0.0 to 0.1)	1.6 (0.5 to 4.9)	5,873 (1,717 to 17,985)
Saint Lucia	0 (0 to 0)	0.1 (0.0 to 0.2)	10.1 (1.5 to 43.6)	101 (15 to 437)	0 (0 to 0)	0.0 (0.0 to 0.0)	2.9 (0.8 to 9.2)	526 (142 to 1,680)
Saint Vincent and the Grenadines	0 (0 to 0)	0.2 (0.0 to 0.4)	6.0 (0.9 to 26.6)	57 (9 to 253)	0 (0 to 0)	0.0 (0.0 to 0.1)	1.8 (0.5 to 5.9)	202 (53 to 659)
Suriname	0 (0 to 1)	0.6 (0.1 to 1.4)	6.3 (1.0 to 27.8)	332 (52 to 1,464)	1 (0 to 1)	0.1 (0.0 to 0.2)	1.9 (0.5 to 6.1)	1,060 (279 to 3,377)
Trinidad and Tobago	0 (0 to 0)	0.1 (0.0 to 0.3)	3.7 (0.6 to 16.4)	326 (50 to 1,458)	0 (0 to 1)	0.0 (0.0 to 0.0)	1.1 (0.3 to 3.5)	1,507 (425 to 4,722)
Virgin Islands, U.S.	0 (0 to 0)	0.0 (0.0 to 0.0)	7.4 (1.1 to 33.6)	40 (6 to 184)	0 (0 to 0)	0.0 (0.0 to 0.0)	1.8 (0.5 to 5.7)	191 (57 to 599)
Tropical Latin America	182 (117 to 265)	1.1 (0.7 to 1.6)	311.6 (174.3 to 489.1)	4,443,578 (2,485,666 to 6,973,325)	683 (484 to 885)	0.3 (0.2 to 0.4)	57.4 (37.1 to 79.1)	12,324,291 (7,970,931 to 16,976,528)
Brazil	173 (109 to 251)	1.1 (0.7 to 1.6)	303.8 (169.7 to 476.1)	4,236,833 (2,366,474 to 6,640,171)	659 (467 to 851)	0.3 (0.2 to 0.4)	56.3 (36.5 to 77.5)	11,720,225 (7,598,884 to 16,142,762)
Paraguay	9 (5 to 15)	1.4 (0.8 to 2.3)	660.8 (365.8 to 1,043.4)	206,679 (114,429 to 326,355)	23 (14 to 39)	0.4 (0.2 to 0.6)	94.8 (60.2 to 135.2)	603,701 (383,509 to 860,527)
Southeast Asia, East Asia, and Oceania	2,702 (1,686 to 4,108)	2.2 (1.4 to 3.3)	85.1 (45.5 to 152.3)	10,739,748 (5,748,432 to 19,223,075)	14,471 (8,436 to 22,888)	0.7 (0.4 to 1.1)	22.4 (14.6 to 30.8)	46,668,439 (30,538,782 to 64,191,055)

Location	Under-5				All ages			
	Deaths	Deaths per 100,000	Incidence per 1000	Cases	Deaths	Deaths per 100,000	Incidence per 1000	Cases
East Asia	133 (79 to 211)	0.2 (0.1 to 0.3)	22.6 (11.4 to 40.4)	1,493,428 (755,242 to 2,669,927)	391 (238 to 677)	0.0 (0.0 to 0.0)	5.2 (3.2 to 7.6)	7,376,462 (4,478,357 to 10,810,483)
China	93 (56 to 141)	0.2 (0.1 to 0.2)	20.3 (10.2 to 35.9)	1,251,582 (628,791 to 2,214,886)	336 (199 to 595)	0.0 (0.0 to 0.0)	4.8 (2.9 to 7.1)	6,584,941 (3,996,142 to 9,673,921)
North Korea	40 (18 to 79)	1.4 (0.6 to 2.7)	60.3 (29.7 to 107.1)	198,168 (97,597 to 351,788)	48 (24 to 87)	0.2 (0.1 to 0.3)	20.5 (11.9 to 31.2)	548,225 (318,579 to 836,970)
Taiwan	1 (0 to 1)	0.1 (0.0 to 0.1)	42.4 (19.8 to 78.7)	44,272 (20,692 to 82,152)	6 (3 to 11)	0.0 (0.0 to 0.0)	10.2 (5.9 to 15.4)	242,831 (140,311 to 366,238)
Southeast Asia	2,427 (1,503 to 3,719)	4.3 (2.6 to 6.5)	149.3 (78.9 to 269.6)	8,775,161 (4,639,168 to 15,844,104)	13,337 (7,670 to 21,358)	2.0 (1.2 to 3.3)	56.5 (36.5 to 77.7)	37,127,957 (23,990,739 to 51,027,995)
Cambodia	46 (19 to 87)	2.4 (1.0 to 4.6)	167.0 (69.7 to 318.7)	306,014 (127,640 to 584,041)	186 (96 to 335)	1.2 (0.6 to 2.1)	59.8 (33.6 to 94.0)	950,137 (534,543 to 1,493,848)
Indonesia	1,487 (956 to 2,226)	6.5 (4.2 to 9.8)	199.0 (106.3 to 341.9)	4,782,520 (2,554,317 to 8,219,562)	9,830 (5,354 to 16,127)	3.8 (2.1 to 6.3)	77.9 (51.2 to 105.0)	20,202,474 (13,285,368 to 27,216,084)
Laos	176 (63 to 383)	15.6 (5.6 to 33.9)	235.1 (121.4 to 424.4)	253,804 (131,004 to 458,053)	254 (118 to 465)	3.5 (1.6 to 6.5)	87.8 (56.3 to 127.2)	628,558 (403,224 to 910,520)
Malaysia	8 (4 to 15)	0.3 (0.2 to 0.6)	129.7 (50.2 to 285.4)	337,607 (130,542 to 742,896)	75 (41 to 129)	0.2 (0.1 to 0.4)	53.1 (30.5 to 83.1)	1,635,865 (939,397 to 2,557,084)
Maldives	0 (0 to 0)	0.3 (0.1 to 0.5)	82.8 (34.6 to 177.0)	3,051 (1,274 to 6,522)	0 (0 to 1)	0.1 (0.1 to 0.2)	38.1 (22.4 to 58.9)	14,036 (8,255 to 21,733)
Mauritius	1 (0 to 1)	1.0 (0.5 to 1.7)	111.9 (46.9 to 239.0)	7,417 (3,110 to 15,847)	4 (3 to 6)	0.3 (0.2 to 0.5)	47.1 (27.9 to 71.6)	60,023 (35,523 to 91,181)
Myanmar	190 (74 to 389)	4.1 (1.6 to 8.3)	106.0 (44.8 to 217.8)	663,294 (280,239 to 1,362,454)	1,106 (590 to 1,949)	2.0 (1.1 to 3.6)	54.0 (33.0 to 80.4)	3,028,737 (1,849,727 to 4,511,893)
Philippines	463 (213 to 892)	4.0 (1.8 to 7.7)	122.7 (53.4 to 248.3)	1,419,350 (617,723 to 2,873,125)	1,113 (669 to 1,694)	1.1 (0.7 to 1.7)	43.9 (25.7 to 67.6)	4,496,129 (2,636,686 to 6,930,218)

	Under-5				All ages			
Location	Deaths	Deaths per 100,000	Incidence per 1000	Cases	Deaths	Deaths per 100,000	Incidence per 1000	Cases
Sri Lanka	3 (1 to 7)	0.2 (0.1 to 0.4)	120.0 (49.8 to 255.5)	167,166 (69,363 to 355,898)	82 (37 to 174)	0.4 (0.2 to 0.8)	43.8 (26.0 to 65.1)	902,109 (535,177 to 1,340,178)
Seychelles	0 (0 to 0)	0.2 (0.1 to 0.4)	122.1 (49.9 to 262.3)	1,026 (419 to 2,204)	0 (0 to 0)	0.3 (0.2 to 0.5)	47.7 (28.6 to 71.7)	4,629 (2,780 to 6,960)
Thailand	8 (4 to 14)	0.2 (0.1 to 0.4)	77.1 (39.0 to 135.9)	192,880 (97,513 to 339,860)	418 (215 to 809)	0.6 (0.3 to 1.2)	29.0 (18.8 to 39.6)	1,940,279 (1,258,369 to 2,649,033)
Timor-Leste	33 (13 to 70)	20.1 (7.6 to 42.1)	234.8 (100.1 to 482.1)	38,600 (16,451 to 79,267)	44 (18 to 86)	3.8 (1.6 to 7.4)	88.3 (50.4 to 141.1)	102,198 (58,305 to 163,337)
Vietnam	11 (5 to 20)	0.1 (0.1 to 0.3)	83.2 (41.3 to 150.8)	595,698 (295,272 to 1,079,273)	224 (93 to 465)	0.2 (0.1 to 0.5)	33.1 (20.5 to 47.1)	3,105,535 (1,926,225 to 4,420,217)
Oceania	141 (67 to 265)	10.0 (4.8 to 18.7)	345.3 (199.9 to 564.1)	476,835 (275,998 to 778,874)	743 (419 to 1,259)	6.6 (3.7 to 11.2)	190.9 (128.1 to 252.9)	2,133,859 (1,431,886 to 2,826,367)
American Samoa	0 (0 to 0)	0.7 (0.4 to 1.2)	274.1 (155.3 to 453.0)	2,049 (1,161 to 3,386)	0 (0 to 1)	0.5 (0.3 to 0.9)	173.8 (113.3 to 234.2)	14,024 (9,144 to 18,898)
Federated States of Micronesia	0 (0 to 0)	1.2 (0.5 to 2.3)	229.4 (130.6 to 378.6)	2,268 (1,291 to 3,742)	1 (1 to 2)	1.2 (0.6 to 2.3)	153.7 (102.1 to 205.4)	15,863 (10,535 to 21,202)
Fiji	5 (2 to 10)	9.3 (4.1 to 19.5)	275.2 (160.9 to 450.2)	14,989 (8,763 to 24,516)	23 (13 to 37)	2.6 (1.5 to 4.3)	141.8 (94.5 to 185.3)	122,729 (81,772 to 160,396)
Guam	0 (0 to 0)	0.3 (0.1 to 0.5)	286.5 (160.6 to 478.5)	5,006 (2,806 to 8,362)	1 (0 to 1)	0.3 (0.2 to 0.5)	175.7 (115.6 to 230.0)	30,780 (20,256 to 40,284)
Kiribati	2 (1 to 5)	17.8 (7.3 to 37.2)	348.2 (199.2 to 582.5)	4,733 (2,708 to 7,918)	10 (5 to 18)	9.0 (4.7 to 16.0)	185.7 (124.2 to 245.0)	21,079 (14,100 to 27,804)
Marshall Islands	0 (0 to 0)	1.6 (0.6 to 3.4)	263.1 (151.9 to 431.6)	2,585 (1,493 to 4,241)	1 (0 to 1)	0.9 (0.5 to 1.6)	160.5 (106.0 to 213.9)	11,920 (7,870 to 15,881)
Northern Mariana Islands	0 (0 to 0)	0.1 (0.0 to 0.2)	387.9 (213.5 to 673.9)	6,010 (3,308 to 10,442)	0 (0 to 0)	0.1 (0.1 to 0.2)	184.3 (121.3 to 246.0)	23,424 (15,425 to 31,267)

	Under-5				All ages			
Location	Deaths	Deaths per 100,000	Incidence per 1000	Cases	Deaths	Deaths per 100,000	Incidence per 1000	Cases
Papua New Guinea	124 (53 to 245)	11.5 (4.9 to 22.8)	367.5 (211.9 to 602.3)	382,791 (220,780 to 627,416)	669 (374 to 1,141)	8.5 (4.8 to 14.5)	211.4 (141.5 to 281.4)	1,652,299 (1,105,555 to 2,199,216)
Samoa	0 (0 to 0)	0.5 (0.2 to 1.2)	293.5 (166.1 to 490.3)	8,107 (4,588 to 13,543)	2 (1 to 4)	0.9 (0.5 to 1.8)	183.3 (120.4 to 245.1)	36,420 (23,926 to 48,701)
Solomon Islands	7 (3 to 12)	8.2 (3.9 to 15.0)	358.9 (206.1 to 586.1)	29,425 (16,897 to 48,045)	24 (13 to 43)	4.0 (2.2 to 7.1)	204.3 (135.0 to 272.0)	121,577 (80,351 to 161,893)
Tonga	0 (0 to 0)	1.5 (0.6 to 3.1)	271.6 (158.6 to 442.4)	3,719 (2,171 to 6,056)	1 (1 to 2)	1.2 (0.6 to 2.1)	180.2 (119.2 to 239.4)	19,447 (12,866 to 25,843)
Vanuatu	3 (1 to 5)	6.7 (3.0 to 13.5)	375.3 (219.2 to 602.6)	15,154 (8,850 to 24,335)	11 (6 to 22)	4.1 (2.2 to 7.9)	219.4 (145.2 to 291.0)	60,644 (40,139 to 80,438)
North Africa and Middle East	1,823 (952 to 3,075)	2.9 (1.5 to 4.9)	107.3 (48.3 to 208.1)	6,814,981 (3,068,712 to 13,219,732)	2,744 (1,566 to 4,259)	0.5 (0.3 to 0.7)	40.4 (24.3 to 60.9)	23,221,621 (13,967,835 to 35,010,094)
North Africa and Middle East	1,823 (952 to 3,075)	2.9 (1.5 to 4.9)	107.3 (48.3 to 208.1)	6,814,981 (3,068,712 to 13,219,732)	2,744 (1,566 to 4,259)	0.5 (0.3 to 0.7)	40.4 (24.3 to 60.9)	23,221,621 (13,967,835 to 35,010,094)
Afghanistan	351 (141 to 730)	7.1 (2.8 to 14.7)	147.5 (49.2 to 323.7)	699,216 (233,196 to 1,535,169)	417 (196 to 797)	1.2 (0.6 to 2.4)	53.9 (27.4 to 94.4)	1,789,465 (910,140 to 3,131,364)
Algeria	18 (7 to 39)	0.4 (0.1 to 0.8)	64.2 (22.1 to 135.4)	326,574 (112,411 to 689,106)	63 (32 to 114)	0.2 (0.1 to 0.3)	26.9 (14.3 to 44.4)	1,099,265 (584,684 to 1,811,961)
Bahrain	0 (0 to 0)	0.1 (0.0 to 0.1)	76.5 (25.2 to 181.2)	7,375 (2,431 to 17,474)	1 (0 to 2)	0.1 (0.0 to 0.1)	23.8 (12.5 to 39.9)	32,934 (17,291 to 55,331)
Egypt	439 (210 to 826)	4.0 (1.9 to 7.6)	55.9 (23.2 to 101.6)	668,051 (277,980 to 1,214,565)	563 (289 to 997)	0.6 (0.3 to 1.1)	23.4 (13.9 to 35.0)	2,168,632 (1,287,096 to 3,241,846)
Iran	54 (17 to 124)	0.7 (0.2 to 1.5)	243.5 (130.8 to 409.9)	1,727,027 (927,848 to 2,907,170)	262 (150 to 488)	0.3 (0.2 to 0.6)	93.4 (60.5 to 126.4)	7,507,681 (4,862,146 to 10,160,779)
Iraq	127 (41 to 267)	1.6 (0.5 to 3.5)	98.3 (32.2 to 211.4)	727,937 (238,245 to 1,565,006)	207 (101 to 362)	0.5 (0.3 to 0.9)	39.0 (19.4 to 69.5)	1,525,735 (759,029 to 2,717,889)
Jordan	0 (0 to 1)	0.0 (0.0 to 0.1)	9.5 (0.9 to 30.9)	9,083 (911 to 29,616)	1 (1 to 2)	0.0 (0.0 to 0.0)	5.1 (1.5 to 10.8)	39,535 (11,215 to 83,078)

Location	Under-5				All ages			
	Deaths	Deaths per 100,000	Incidence per 1000	Cases	Deaths	Deaths per 100,000	Incidence per 1000	Cases
Kuwait	0 (0 to 1)	0.1 (0.0 to 0.2)	35.3 (11.7 to 81.6)	23,532 (7,768 to 54,320)	1 (0 to 1)	0.0 (0.0 to 0.0)	25.2 (13.4 to 42.2)	108,804 (58,062 to 182,514)
Lebanon	1 (0 to 2)	0.3 (0.1 to 0.6)	98.0 (33.4 to 212.5)	29,551 (10,083 to 64,066)	6 (3 to 14)	0.1 (0.0 to 0.2)	29.0 (15.9 to 46.4)	168,271 (92,311 to 269,595)
Libya	2 (1 to 4)	0.4 (0.2 to 0.8)	100.6 (42.8 to 203.9)	49,496 (21,067 to 100,318)	7 (4 to 12)	0.1 (0.1 to 0.2)	46.1 (27.4 to 70.7)	285,340 (169,446 to 437,933)
Morocco	74 (31 to 147)	3.2 (1.4 to 6.4)	90.8 (30.4 to 201.3)	203,715 (68,313 to 451,836)	123 (64 to 214)	0.4 (0.2 to 0.6)	26.3 (14.2 to 43.6)	883,680 (477,592 to 1,464,320)
Palestine	3 (1 to 6)	0.2 (0.1 to 0.5)	90.6 (29.5 to 200.6)	96,922 (31,600 to 214,525)	5 (3 to 9)	0.1 (0.1 to 0.2)	39.0 (19.3 to 70.0)	200,819 (99,286 to 360,580)
Oman	0 (0 to 0)	0.1 (0.0 to 0.1)	70.6 (23.8 to 157.4)	30,398 (10,243 to 67,773)	2 (1 to 3)	0.0 (0.0 to 0.1)	26.8 (13.9 to 43.6)	125,312 (65,184 to 203,984)
Qatar	0 (0 to 0)	0.1 (0.0 to 0.2)	45.9 (15.4 to 105.6)	5,812 (1,946 to 13,377)	0 (0 to 0)	0.0 (0.0 to 0.0)	12.6 (6.6 to 21.3)	28,837 (15,018 to 48,650)
Saudi Arabia	4 (2 to 6)	0.2 (0.1 to 0.3)	79.1 (43.5 to 133.6)	201,810 (110,824 to 340,742)	33 (17 to 56)	0.1 (0.1 to 0.2)	35.2 (23.2 to 47.7)	1,112,989 (731,360 to 1,506,341)
Sudan	234 (63 to 562)	5.6 (1.5 to 13.4)	132.9 (35.1 to 290.3)	490,564 (129,527 to 1,071,843)	406 (158 to 811)	1.0 (0.4 to 2.1)	37.6 (17.8 to 65.4)	1,460,248 (690,922 to 2,539,588)
Syria	3 (1 to 6)	0.2 (0.1 to 0.3)	64.5 (21.7 to 149.5)	125,368 (42,073 to 290,378)	7 (3 to 12)	0.0 (0.0 to 0.1)	27.4 (14.2 to 48.0)	503,787 (261,663 to 881,859)
Tunisia	1 (0 to 2)	0.1 (0.0 to 0.2)	28.8 (6.8 to 65.8)	26,701 (6,336 to 61,076)	10 (4 to 24)	0.1 (0.0 to 0.2)	12.6 (6.2 to 20.9)	142,072 (70,582 to 236,011)
Turkey	17 (7 to 35)	0.3 (0.1 to 0.6)	81.3 (27.1 to 186.8)	506,539 (168,943 to 1,164,383)	58 (31 to 107)	0.1 (0.0 to 0.1)	28.0 (15.0 to 46.4)	2,226,276 (1,193,836 to 3,685,159)
United Arab Emirates	0 (0 to 1)	0.1 (0.0 to 0.1)	75.6 (24.7 to 176.3)	70,369 (23,027 to 164,157)	4 (2 to 8)	0.0 (0.0 to 0.1)	23.4 (12.0 to 39.7)	226,837 (116,891 to 385,902)

Location	Under-5				All ages			
	Deaths	Deaths per 100,000	Incidence per 1000	Cases	Deaths	Deaths per 100,000	Incidence per 1000	Cases
Yemen	494 (162 to 1,059)	10.7 (3.5 to 22.9)	181.8 (61.6 to 392.1)	821,205 (278,413 to 1,771,091)	569 (214 to 1,153)	2.0 (0.8 to 4.1)	58.9 (29.5 to 105.4)	1,650,979 (827,908 to 2,953,977)
South Asia	10,443 (6,658 to 15,566)	6.8 (4.3 to 10.1)	89.5 (51.1 to 143.0)	14,308,810 (8,166,722 to 22,853,542)	78,392 (47,670 to 134,099)	4.6 (2.8 to 7.9)	43.2 (28.9 to 57.2)	73,683,839 (49,272,837 to 97,539,370)
South Asia	10,443 (6,658 to 15,566)	6.8 (4.3 to 10.1)	89.5 (51.1 to 143.0)	14,308,810 (8,166,722 to 22,853,542)	78,392 (47,670 to 134,099)	4.6 (2.8 to 7.9)	43.2 (28.9 to 57.2)	73,683,839 (49,272,837 to 97,539,370)
Bangladesh	559 (312 to 917)	3.9 (2.2 to 6.4)	142.0 (84.2 to 224.1)	2,118,364 (1,256,386 to 3,343,394)	5,721 (3,098 to 11,181)	3.5 (1.9 to 6.9)	73.4 (49.9 to 94.8)	11,924,941 (8,109,770 to 15,405,909)
Bhutan	2 (1 to 4)	2.8 (1.2 to 5.6)	391.1 (209.5 to 632.3)	29,579 (15,842 to 47,817)	11 (2 to 27)	1.4 (0.3 to 3.4)	172.6 (111.2 to 235.3)	137,316 (88,425 to 187,143)
India	4,643 (2,988 to 6,999)	4.1 (2.7 to 6.2)	47.5 (26.2 to 81.1)	5,515,026 (3,041,012 to 9,420,939)	61,705 (36,417 to 104,880)	4.7 (2.8 to 8.0)	32.1 (21.2 to 43.4)	42,311,398 (27,994,070 to 57,291,328)
Nepal	144 (62 to 274)	3.6 (1.6 to 6.9)	201.0 (109.1 to 340.6)	846,208 (459,155 to 1,433,823)	1,501 (763 to 2,661)	5.0 (2.5 to 8.8)	91.6 (60.5 to 128.0)	2,776,217 (1,831,703 to 3,877,565)
Pakistan	5,094 (2,973 to 8,143)	22.1 (12.9 to 35.3)	241.3 (135.8 to 377.3)	5,897,218 (3,319,900 to 9,221,246)	9,454 (5,561 to 16,135)	5.0 (2.9 to 8.4)	84.9 (55.5 to 113.6)	16,324,834 (10,679,347 to 21,841,855)
Sub-Saharan Africa	47,795 (30,144 to 71,661)	30.5 (19.3 to 45.8)	213.5 (117.7 to 372.4)	33,729,011 (18,586,036 to 58,822,680)	113,199 (73,683 to 168,125)	11.5 (7.5 to 17.2)	99.2 (64.9 to 136.9)	97,363,433 (63,741,963 to 134,361,302)
Southern Sub-Saharan Africa	1,741 (1,117 to 2,652)	20.2 (13.0 to 30.8)	189.4 (109.5 to 301.6)	1,716,531 (992,173 to 2,733,071)	4,726 (2,946 to 7,279)	6.1 (3.8 to 9.5)	107.9 (73.3 to 139.2)	8,351,367 (5,673,379 to 10,780,978)
Botswana	34 (14 to 62)	12.9 (5.4 to 23.6)	146.4 (80.0 to 253.1)	39,834 (21,782 to 68,876)	135 (62 to 246)	5.9 (2.7 to 10.7)	74.0 (49.1 to 98.0)	170,627 (113,052 to 225,832)
Lesotho	154 (88 to 250)	59.7 (34.0 to 96.9)	232.3 (132.5 to 378.0)	58,646 (33,446 to 95,445)	428 (220 to 734)	20.0 (10.3 to 34.4)	111.3 (73.6 to 147.7)	237,020 (156,692 to 314,531)
Namibia	100 (51 to 173)	30.1 (15.4 to 51.9)	225.8 (122.1 to 377.9)	76,909 (41,588 to 128,715)	201 (110 to 315)	8.1 (4.4 to 12.6)	98.8 (64.4 to 132.8)	247,593 (161,495 to 332,927)

Location	Under-5				All ages			
	Deaths	Deaths per 100,000	Incidence per 1000	Cases	Deaths	Deaths per 100,000	Incidence per 1000	Cases
South Africa	412 (237 to 671)	8.2 (4.7 to 13.4)	176.7 (101.8 to 279.4)	955,202 (550,403 to 1,509,864)	2,137 (1,241 to 3,535)	4.1 (2.4 to 6.7)	113.0 (78.2 to 143.7)	6,006,660 (4,154,649 to 7,639,370)
Swaziland	89 (49 to 150)	42.6 (23.3 to 71.9)	213.7 (119.5 to 366.1)	43,689 (24,425 to 74,839)	166 (98 to 258)	12.4 (7.3 to 19.3)	105.9 (69.3 to 143.6)	141,291 (92,393 to 191,557)
Zimbabwe	951 (563 to 1,540)	37.6 (22.2 to 60.8)	209.0 (120.7 to 335.9)	541,004 (312,564 to 869,608)	1,660 (1,013 to 2,573)	10.4 (6.3 to 16.1)	96.4 (63.2 to 130.4)	1,543,578 (1,012,362 to 2,088,404)
Western Sub-Saharan Africa	29,027 (17,665 to 45,045)	44.9 (27.3 to 69.7)	210.6 (117.0 to 355.1)	13,751,383 (7,637,743 to 23,188,393)	45,813 (28,828 to 68,003)	11.5 (7.2 to 17.1)	82.5 (53.2 to 116.0)	32,898,776 (21,218,837 to 46,292,357)
Benin	715 (321 to 1,302)	37.4 (16.8 to 68.1)	133.4 (51.9 to 276.0)	260,626 (101,305 to 539,041)	1,129 (621 to 1,832)	10.0 (5.5 to 16.2)	54.1 (30.4 to 87.5)	615,371 (345,655 to 996,369)
Burkina Faso	304 (146 to 559)	9.5 (4.6 to 17.5)	73.3 (31.0 to 141.1)	236,017 (99,803 to 454,121)	795 (456 to 1,285)	4.3 (2.4 to 6.9)	33.8 (18.8 to 52.1)	630,563 (350,248 to 970,979)
Cameroon	646 (258 to 1,278)	16.7 (6.7 to 33.0)	181.4 (69.5 to 373.6)	713,293 (273,229 to 1,469,201)	1,386 (760 to 2,378)	5.8 (3.2 to 9.9)	63.8 (35.0 to 104.5)	1,535,376 (840,879 to 2,514,174)
Cape Verde	1 (0 to 2)	1.4 (0.6 to 2.6)	99.7 (37.5 to 212.5)	7,414 (2,785 to 15,798)	4 (2 to 8)	0.8 (0.4 to 1.4)	44.6 (25.7 to 69.1)	24,456 (14,097 to 37,861)
Chad	1,772 (774 to 3,261)	67.1 (29.3 to 123.5)	267.3 (103.2 to 535.4)	689,231 (266,122 to 1,380,410)	2,427 (1,293 to 4,071)	16.8 (9.0 to 28.2)	91.2 (49.6 to 149.8)	1,309,920 (712,221 to 2,153,375)
Cote d'Ivoire	1,241 (558 to 2,353)	34.9 (15.7 to 66.1)	190.3 (73.4 to 393.1)	665,285 (256,535 to 1,374,333)	1,829 (991 to 3,136)	7.9 (4.3 to 13.6)	62.9 (35.0 to 102.0)	1,446,747 (804,874 to 2,345,553)
The Gambia	72 (38 to 123)	19.6 (10.5 to 33.6)	227.7 (122.8 to 380.8)	83,625 (45,107 to 139,841)	130 (76 to 221)	6.4 (3.7 to 10.8)	94.4 (60.6 to 132.6)	193,003 (123,878 to 271,157)
Ghana	179 (79 to 341)	4.1 (1.8 to 7.9)	117.2 (47.5 to 242.4)	541,022 (219,383 to 1,118,684)	537 (296 to 946)	1.9 (1.0 to 3.3)	45.9 (26.1 to 72.4)	1,308,023 (743,892 to 2,063,019)
Guinea	332 (138 to 656)	16.3 (6.8 to 32.2)	183.9 (71.6 to 374.0)	367,956 (143,370 to 748,563)	761 (434 to 1,331)	5.9 (3.4 to 10.3)	66.2 (36.6 to 106.0)	850,690 (470,801 to 1,362,690)

Location	Under-5 Deaths	Under-5 Deaths per 100,000	Under-5 Incidence per 1000	Under-5 Cases	All ages Deaths	All ages Deaths per 100,000	All ages Incidence per 1000	All ages Cases
Guinea-Bissau	29 (13 to 53)	9.4 (4.3 to 17.2)	57.4 (19.3 to 123.5)	17,538 (5,882 to 37,726)	64 (35 to 111)	3.4 (1.8 to 5.9)	22.9 (11.2 to 37.7)	43,445 (21,277 to 71,602)
Liberia	269 (121 to 484)	37.7 (17.0 to 67.8)	223.9 (86.7 to 451.7)	158,960 (61,559 to 320,693)	495 (276 to 848)	10.7 (6.0 to 18.4)	79.6 (44.0 to 127.4)	367,453 (203,094 to 587,969)
Mali	402 (180 to 771)	12.8 (5.7 to 24.5)	32.9 (5.2 to 77.7)	101,354 (15,890 to 239,409)	742 (389 to 1,254)	4.1 (2.2 to 7.0)	15.4 (5.8 to 27.9)	275,518 (104,610 to 499,438)
Mauritania	78 (34 to 145)	15.2 (6.6 to 28.1)	175.0 (70.4 to 348.3)	87,363 (35,126 to 173,829)	204 (107 to 371)	5.0 (2.6 to 9.1)	60.4 (34.5 to 94.6)	245,092 (139,716 to 383,657)
Niger	1,213 (556 to 2,326)	32.9 (15.1 to 63.1)	108.8 (47.3 to 210.6)	403,580 (175,489 to 781,123)	1,963 (1,069 to 3,300)	9.8 (5.3 to 16.4)	49.6 (28.3 to 76.4)	997,180 (568,032 to 1,536,493)
Nigeria	20,320 (11,818 to 32,537)	68.8 (40.0 to 110.2)	272.1 (157.3 to 437.5)	8,170,468 (4,722,548 to 13,136,816)	30,275 (18,478 to 45,698)	16.4 (10.0 to 24.7)	108.3 (70.8 to 146.9)	20,069,335 (13,122,511 to 27,216,995)
Sao Tome and Principe	2 (1 to 5)	7.0 (2.5 to 14.4)	157.0 (61.2 to 322.0)	5,425 (2,115 to 11,127)	4 (2 to 7)	2.0 (1.0 to 3.6)	58.4 (32.1 to 96.7)	11,627 (6,394 to 19,249)
Senegal	844 (461 to 1,417)	33.2 (18.1 to 55.7)	346.8 (203.3 to 553.5)	888,887 (520,951 to 1,418,567)	1,862 (1,164 to 2,837)	12.0 (7.5 to 18.4)	136.4 (90.2 to 187.5)	2,111,326 (1,395,894 to 2,901,519)
Sierra Leone	477 (206 to 880)	45.8 (19.8 to 84.7)	159.2 (61.9 to 330.3)	165,716 (64,429 to 343,794)	817 (441 to 1,331)	12.3 (6.7 to 20.1)	61.3 (34.1 to 99.7)	405,868 (225,974 to 660,543)
Togo	130 (52 to 249)	11.9 (4.7 to 22.7)	160.8 (63.3 to 326.7)	175,690 (69,119 to 356,981)	388 (213 to 690)	5.2 (2.9 to 9.3)	61.0 (34.5 to 96.1)	451,819 (256,067 to 712,247)
Eastern Sub-Saharan Africa	14,934 (9,448 to 22,501)	23.9 (15.1 to 36.0)	259.0 (142.8 to 448.5)	16,154,581 (8,907,765 to 27,978,045)	57,473 (36,018 to 90,963)	14.8 (9.3 to 23.5)	133.1 (87.0 to 181.5)	51,479,886 (33,640,524 to 70,237,981)
Burundi	1,628 (837 to 2,839)	76.0 (39.1 to 132.5)	338.6 (176.5 to 615.2)	716,299 (373,306 to 1,301,369)	3,037 (1,774 to 4,899)	26.2 (15.3 to 42.3)	150.0 (95.6 to 216.7)	1,731,913 (1,103,785 to 2,501,169)
Comoros	14 (6 to 28)	14.8 (6.6 to 29.6)	265.7 (134.0 to 499.6)	25,830 (13,026 to 48,564)	98 (50 to 182)	12.6 (6.4 to 23.3)	139.0 (88.0 to 196.8)	108,873 (68,951 to 154,150)

	Under-5				All ages			
Location	Deaths	Deaths per 100,000	Incidence per 1000	Cases	Deaths	Deaths per 100,000	Incidence per 1000	Cases
Djibouti	17 (7 to 36)	9.6 (3.8 to 20.7)	256.1 (125.5 to 507.9)	32,098 (15,725 to 63,660)	74 (36 to 148)	7.7 (3.8 to 15.2)	101.2 (63.7 to 145.8)	93,289 (58,740 to 134,414)
Eritrea	265 (126 to 474)	33.3 (15.8 to 59.6)	290.9 (143.4 to 570.3)	218,322 (107,591 to 427,956)	774 (450 to 1,259)	14.5 (8.4 to 23.6)	130.6 (82.0 to 189.0)	690,501 (433,595 to 999,242)
Ethiopia	3,577 (1,987 to 5,786)	23.4 (13.0 to 37.9)	332.9 (188.5 to 535.4)	4,918,388 (2,785,016 to 7,910,055)	16,454 (9,932 to 27,358)	16.1 (9.7 to 26.7)	163.3 (106.4 to 221.8)	16,628,055 (10,829,949 to 22,577,930)
Kenya	1,046 (677 to 1,533)	15.9 (10.3 to 23.3)	168.7 (94.0 to 277.3)	1,111,054 (619,059 to 1,826,660)	10,593 (5,798 to 17,018)	22.7 (12.4 to 36.5)	126.0 (84.2 to 165.5)	5,870,399 (3,924,222 to 7,708,623)
Madagascar	2,418 (1,132 to 4,394)	62.2 (29.1 to 113.0)	320.7 (159.1 to 605.8)	1,238,874 (614,467 to 2,340,101)	4,788 (2,777 to 7,634)	19.2 (11.1 to 30.6)	130.4 (81.2 to 188.5)	3,248,822 (2,022,073 to 4,697,024)
Malawi	792 (408 to 1,389)	24.7 (12.7 to 43.4)	290.4 (144.6 to 556.1)	937,954 (467,099 to 1,796,100)	2,593 (1,528 to 4,271)	14.4 (8.5 to 23.8)	133.4 (82.8 to 194.4)	2,398,509 (1,489,045 to 3,493,624)
Mozambique	675 (375 to 1,143)	13.6 (7.5 to 23.0)	147.9 (84.5 to 237.1)	742,805 (424,490 to 1,190,479)	1,930 (1,034 to 3,323)	6.7 (3.6 to 11.5)	80.8 (53.0 to 109.4)	2,331,033 (1,530,635 to 3,157,831)
Rwanda	316 (154 to 569)	16.9 (8.2 to 30.4)	214.9 (111.2 to 405.1)	398,829 (206,263 to 751,674)	1,025 (526 to 1,739)	8.5 (4.4 to 14.4)	108.7 (69.7 to 158.1)	1,310,356 (840,267 to 1,906,377)
Somalia	543 (250 to 1,075)	41.2 (18.9 to 81.5)	426.4 (256.6 to 692.9)	504,236 (303,446 to 819,373)	2,262 (1,156 to 4,441)	21.8 (11.1 to 42.7)	191.9 (127.7 to 258.0)	1,967,604 (1,309,288 to 2,644,555)
South Sudan	692 (300 to 1,443)	24.2 (10.5 to 50.5)	353.7 (174.0 to 689.6)	1,054,417 (518,802 to 2,055,617)	2,437 (1,415 to 4,158)	17.9 (10.4 to 30.6)	197.7 (122.9 to 287.8)	2,709,955 (1,684,750 to 3,944,467)
Tanzania	1,138 (612 to 1,969)	12.6 (6.8 to 21.9)	160.9 (86.6 to 277.9)	1,511,020 (813,210 to 2,609,639)	6,357 (3,582 to 10,857)	11.7 (6.6 to 19.9)	96.5 (63.4 to 132.2)	5,298,059 (3,481,527 to 7,255,338)
Uganda	1,434 (733 to 2,423)	19.1 (9.8 to 32.2)	302.7 (148.5 to 586.8)	2,284,551 (1,120,811 to 4,428,753)	3,688 (2,183 to 5,985)	9.1 (5.4 to 14.8)	143.2 (88.7 to 211.0)	5,788,480 (3,584,856 to 8,528,305)
Zambia	381 (186 to 677)	13.4 (6.5 to 23.8)	162.7 (81.7 to 295.6)	459,101 (230,504 to 834,332)	1,363 (773 to 2,233)	8.2 (4.6 to 13.4)	77.9 (49.2 to 116.1)	1,294,997 (818,931 to 1,930,364)

Location	Under-5				All ages			
	Deaths	Deaths per 100,000	Incidence per 1000	Cases	Deaths	Deaths per 100,000	Incidence per 1000	Cases
Central Sub-Saharan Africa	2,094 (950 to 3,702)	10.1 (4.6 to 17.8)	97.1 (33.6 to 205.2)	2,062,550 (714,274 to 4,357,213)	5,186 (2,966 to 8,346)	4.4 (2.5 to 7.1)	39.4 (19.5 to 69.7)	4,660,748 (2,307,383 to 8,243,199)
Angola	177 (56 to 384)	3.6 (1.1 to 7.8)	20.6 (1.7 to 66.9)	102,811 (8,331 to 334,658)	395 (187 to 703)	1.5 (0.7 to 2.7)	8.9 (1.5 to 21.6)	232,559 (37,846 to 562,378)
Central African Republic	406 (217 to 715)	54.7 (29.2 to 96.3)	308.6 (158.5 to 525.9)	244,149 (125,374 to 416,041)	1,051 (616 to 1,678)	21.0 (12.3 to 33.4)	120.3 (75.9 to 169.7)	609,285 (384,556 to 859,471)
Congo	52 (19 to 111)	7.0 (2.6 to 14.9)	110.5 (35.7 to 239.7)	82,344 (26,590 to 178,625)	222 (111 to 409)	4.7 (2.4 to 8.7)	42.4 (20.7 to 75.1)	199,876 (97,640 to 353,834)
Democratic Republic of the Congo	1,450 (524 to 2,847)	10.3 (3.7 to 20.3)	111.5 (36.1 to 240.8)	1,599,408 (518,399 to 3,454,820)	3,467 (1,835 to 5,890)	4.4 (2.3 to 7.4)	44.2 (21.5 to 79.0)	3,527,824 (1,714,505 to 6,309,337)
Equatorial Guinea	1 (0 to 2)	0.9 (0.3 to 2.0)	111.7 (34.4 to 245.9)	10,955 (3,372 to 24,126)	7 (2 to 21)	0.8 (0.2 to 2.5)	42.1 (20.5 to 72.0)	35,217 (17,119 to 60,222)
Gabon	9 (3 to 18)	3.5 (1.3 to 7.4)	96.6 (30.2 to 207.9)	23,988 (7,496 to 51,617)	44 (20 to 92)	2.5 (1.1 to 5.2)	36.7 (18.2 to 63.2)	64,982 (32,112 to 111,705)

Supplementary Table 4. Estimated country-level results for ETEC diarrhoea in 2016

Values in parentheses are 95% uncertainty intervals.

	Under-5				All ages			
Location	Deaths	Deaths per 100,000	Incidence per 1000	Cases	Deaths2	Deaths per 100,0003	Incidence per 10004	Cases5
Global	18,669 (9,800 to 30,659)	3.0 (1.6 to 4.9)	116.8 (61.7 to 202.6)	75,163,376 (39,689,144 to 130,352,142)	51,186 (26,757 to 83,064)	0.7 (0.4 to 1.1)	30.1 (19.6 to 43.6)	222,637,561 (144,947,450 to 322,845,099)
High-income	3 (1 to 6)	0.0 (0.0 to 0.0)	8.1 (2.4 to 18.9)	473,026 (138,811 to 1,101,291)	183 (48 to 398)	0.0 (0.0 to 0.0)	1.4 (0.8 to 2.4)	1,518,579 (819,630 to 2,500,418)
High-income North America	0 (0 to 0)	0.0 (0.0 to 0.0)	0.2 (0.1 to 0.3)	4,301 (2,817 to 6,078)	6 (4 to 7)	0.0 (0.0 to 0.0)	0.1 (0.0 to 0.1)	24,644 (17,158 to 31,715)
Canada	0 (0 to 0)	0.0 (0.0 to 0.0)	0.1 (0.1 to 0.2)	260 (163 to 377)	1 (1 to 1)	0.0 (0.0 to 0.0)	0.0 (0.0 to 0.1)	1,605 (1,099 to 2,095)
Greenland	0 (0 to 0)	0.0 (0.0 to 0.0)	0.2 (0.1 to 0.3)	1 (0 to 1)	0 (0 to 0)	0.0 (0.0 to 0.0)	0.1 (0.0 to 0.1)	3 (2 to 4)
United States	0 (0 to 0)	0.0 (0.0 to 0.0)	0.2 (0.1 to 0.3)	4,041 (2,651 to 5,702)	5 (3 to 6)	0.0 (0.0 to 0.0)	0.1 (0.0 to 0.1)	23,037 (16,044 to 29,602)
Australasia	0 (0 to 0)	0.0 (0.0 to 0.0)	0.2 (0.0 to 1.0)	313 (71 to 1,813)	0 (0 to 0)	0.0 (0.0 to 0.0)	0.1 (0.0 to 0.1)	1,532 (791 to 4,153)
Australia	0 (0 to 0)	0.0 (0.0 to 0.0)	0.1 (0.0 to 0.9)	222 (50 to 1,301)	0 (0 to 0)	0.0 (0.0 to 0.0)	0.0 (0.0 to 0.1)	1,119 (578 to 3,032)
New Zealand	0 (0 to 0)	0.0 (0.0 to 0.0)	0.3 (0.1 to 1.8)	90 (21 to 528)	0 (0 to 0)	0.0 (0.0 to 0.0)	0.1 (0.0 to 0.3)	413 (209 to 1,154)
High-income Asia Pacific	0 (0 to 0)	0.0 (0.0 to 0.0)	0.3 (0.1 to 1.6)	2,157 (463 to 12,190)	2 (1 to 3)	0.0 (0.0 to 0.0)	0.0 (0.0 to 0.1)	7,655 (3,726 to 22,502)
Brunei	0 (0 to 0)	0.0 (0.0 to 0.0)	0.2 (0.1 to 1.3)	8 (2 to 45)	0 (0 to 0)	0.0 (0.0 to 0.0)	0.0 (0.0 to 0.2)	19 (8 to 69)
Japan	0 (0 to 0)	0.0 (0.0 to 0.0)	0.3 (0.1 to 1.8)	1,685 (341 to 9,440)	2 (1 to 2)	0.0 (0.0 to 0.0)	0.0 (0.0 to 0.1)	6,046 (2,923 to 17,743)
Singapore	0 (0 to 0)	0.0 (0.0 to 0.0)	0.2 (0.1 to 1.4)	43 (10 to 246)	0 (0 to 0)	0.0 (0.0 to 0.0)	0.0 (0.0 to 0.1)	142 (66 to 446)
South Korea	0 (0 to 0)	0.0 (0.0 to 0.0)	0.2 (0.0 to 1.1)	422 (94 to 2,493)	1 (0 to 1)	0.0 (0.0 to 0.0)	0.0 (0.0 to 0.1)	1,448 (691 to 4,345)
Western Europe	3 (1 to 6)	0.0 (0.0 to 0.0)	20.9 (5.9 to 48.6)	464,291 (131,365 to 1,079,493)	174 (41 to 388)	0.0 (0.0 to 0.1)	3.5 (1.9 to 5.7)	1,487,136 (797,182 to 2,439,141)
Andorra	0 (0 to 0)	0.0 (0.0 to 0.0)	17.8 (0.9 to 54.2)	53 (3 to 162)	0 (0 to 0)	0.0 (0.0 to 0.0)	2.2 (0.5 to 4.5)	171 (43 to 354)

Location	Under-5 Deaths	Under-5 Deaths per 100,000	Under-5 Incidence per 1000	Under-5 Cases	All ages Deaths2	All ages Deaths per 100,0003	All ages Incidence per 10004	All ages Cases5
Austria	0 (0 to 0)	0.0 (0.0 to 0.0)	31.2 (3.8 to 82.6)	12,299 (1,511 to 32,581)	1 (0 to 2)	0.0 (0.0 to 0.0)	4.5 (1.7 to 8.2)	38,730 (14,914 to 71,544)
Belgium	0 (0 to 0)	0.0 (0.0 to 0.0)	22.2 (1.0 to 66.7)	13,758 (651 to 41,434)	3 (0 to 14)	0.0 (0.0 to 0.1)	3.1 (0.7 to 6.6)	34,784 (8,458 to 74,414)
Cyprus	0 (0 to 0)	0.0 (0.0 to 0.1)	21.9 (1.1 to 66.1)	1,133 (58 to 3,420)	0 (0 to 1)	0.0 (0.0 to 0.1)	3.2 (0.8 to 6.9)	2,879 (731 to 6,268)
Denmark	0 (0 to 0)	0.0 (0.0 to 0.0)	20.0 (1.1 to 60.4)	5,729 (308 to 17,250)	1 (0 to 6)	0.0 (0.0 to 0.1)	2.9 (0.7 to 6.1)	16,373 (4,235 to 34,785)
Finland	0 (0 to 0)	0.0 (0.0 to 0.0)	12.7 (0.5 to 43.5)	3,705 (142 to 12,677)	0 (0 to 0)	0.0 (0.0 to 0.0)	1.9 (0.3 to 4.6)	10,278 (1,847 to 25,601)
France	1 (0 to 2)	0.0 (0.0 to 0.1)	24.8 (4.5 to 71.8)	96,784 (17,457 to 280,251)	40 (3 to 111)	0.1 (0.0 to 0.2)	4.2 (1.7 to 7.9)	270,480 (111,830 to 513,195)
Germany	0 (0 to 1)	0.0 (0.0 to 0.0)	32.2 (8.9 to 69.8)	111,770 (30,973 to 242,423)	75 (12 to 159)	0.1 (0.0 to 0.2)	5.7 (3.3 to 8.9)	460,266 (263,550 to 722,294)
Greece	0 (0 to 0)	0.0 (0.0 to 0.0)	34.2 (11.4 to 75.9)	16,275 (5,441 to 36,108)	1 (0 to 1)	0.0 (0.0 to 0.0)	5.6 (3.3 to 8.7)	60,620 (35,602 to 94,433)
Iceland	0 (0 to 0)	0.0 (0.0 to 0.0)	18.9 (0.9 to 57.5)	403 (19 to 1,230)	0 (0 to 0)	0.0 (0.0 to 0.0)	2.9 (0.7 to 6.4)	975 (239 to 2,110)
Ireland	0 (0 to 0)	0.0 (0.0 to 0.0)	7.6 (0.3 to 31.9)	2,847 (97 to 11,921)	0 (0 to 0)	0.0 (0.0 to 0.0)	1.2 (0.1 to 3.6)	5,524 (417 to 16,942)
Israel	0 (0 to 0)	0.0 (0.0 to 0.0)	0.9 (0.1 to 5.9)	777 (118 to 4,929)	0 (0 to 0)	0.0 (0.0 to 0.0)	0.2 (0.0 to 0.9)	1,623 (369 to 7,116)
Italy	0 (0 to 0)	0.0 (0.0 to 0.0)	0.2 (0.1 to 0.3)	610 (372 to 864)	0 (0 to 0)	0.0 (0.0 to 0.0)	0.0 (0.0 to 0.1)	2,333 (1,590 to 3,048)
Luxembourg	0 (0 to 0)	0.0 (0.0 to 0.0)	19.7 (1.1 to 58.1)	574 (31 to 1,691)	0 (0 to 0)	0.0 (0.0 to 0.1)	2.7 (0.7 to 5.5)	1,532 (388 to 3,182)
Malta	0 (0 to 0)	0.0 (0.0 to 0.0)	7.2 (0.4 to 22.2)	153 (9 to 473)	0 (0 to 0)	0.0 (0.0 to 0.0)	1.0 (0.3 to 2.1)	418 (108 to 906)
Netherlands	0 (0 to 0)	0.0 (0.0 to 0.0)	25.7 (1.3 to 78.8)	22,385 (1,122 to 68,555)	3 (0 to 12)	0.0 (0.0 to 0.1)	3.6 (0.9 to 7.7)	62,477 (15,415 to 131,610)
Norway	0 (0 to 0)	0.0 (0.0 to 0.0)	16.6 (0.9 to 49.5)	4,713 (248 to 14,006)	2 (0 to 7)	0.0 (0.0 to 0.1)	2.3 (0.6 to 4.8)	12,030 (3,048 to 25,331)
Portugal	0 (0 to 0)	0.0 (0.0 to 0.0)	0.6 (0.1 to 4.1)	268 (67 to 1,908)	0 (0 to 0)	0.0 (0.0 to 0.0)	0.1 (0.0 to 0.3)	806 (344 to 3,065)
Spain	0 (0 to 0)	0.0 (0.0 to 0.0)	11.7 (0.6 to 36.1)	27,716 (1,429 to 85,808)	4 (0 to 18)	0.0 (0.0 to 0.0)	1.7 (0.4 to 3.7)	79,865 (19,236 to 170,737)

	Under-5				All ages			
Location	Deaths	Deaths per 100,000	Incidence per 1000	Cases	Deaths2	Deaths per 100,0003	Incidence per 10004	Cases5
Sweden	0 (0 to 0)	0.0 (0.0 to 0.0)	22.5 (6.9 to 48.7)	12,784 (3,949 to 27,660)	2 (0 to 7)	0.0 (0.0 to 0.1)	3.1 (1.4 to 5.6)	30,792 (13,607 to 55,496)
Switzerland	0 (0 to 0)	0.0 (0.0 to 0.0)	0.5 (0.3 to 0.8)	206 (122 to 346)	0 (0 to 0)	0.0 (0.0 to 0.0)	0.1 (0.1 to 0.1)	793 (534 to 1,210)
England	1 (0 to 1)	0.0 (0.0 to 0.0)	35.0 (16.2 to 67.2)	111,314 (51,461 to 213,316)	35 (14 to 61)	0.1 (0.0 to 0.1)	6.0 (3.7 to 9.0)	329,661 (203,610 to 491,952)
Northern Ireland	0 (0 to 0)	0.0 (0.0 to 0.1)	21.1 (5.6 to 46.1)	2,508 (669 to 5,468)	0 (0 to 1)	0.0 (0.0 to 0.1)	3.9 (2.1 to 6.5)	7,358 (3,830 to 12,207)
Scotland	0 (0 to 0)	0.0 (0.0 to 0.0)	24.5 (8.0 to 52.6)	6,673 (2,170 to 14,333)	3 (1 to 5)	0.1 (0.0 to 0.1)	4.5 (2.6 to 7.0)	24,403 (14,162 to 37,723)
Wales	0 (0 to 0)	0.1 (0.0 to 0.1)	55.6 (21.1 to 112.3)	8,938 (3,396 to 18,061)	3 (1 to 5)	0.1 (0.0 to 0.2)	10.6 (6.7 to 15.6)	33,095 (20,842 to 48,564)
Southern Latin America	0 (0 to 0)	0.0 (0.0 to 0.0)	1.7 (0.4 to 10.3)	8,722 (1,872 to 51,414)	1 (0 to 1)	0.0 (0.0 to 0.0)	0.2 (0.1 to 0.9)	15,869 (5,518 to 61,028)
Argentina	0 (0 to 0)	0.0 (0.0 to 0.0)	2.0 (0.4 to 11.8)	7,008 (1,505 to 41,729)	0 (0 to 1)	0.0 (0.0 to 0.0)	0.3 (0.1 to 1.1)	12,395 (4,174 to 48,624)
Chile	0 (0 to 0)	0.0 (0.0 to 0.0)	1.3 (0.3 to 7.5)	1,561 (343 to 9,202)	0 (0 to 0)	0.0 (0.0 to 0.0)	0.2 (0.1 to 0.6)	3,189 (1,210 to 11,684)
Uruguay	0 (0 to 0)	0.0 (0.0 to 0.0)	0.6 (0.1 to 3.9)	152 (33 to 937)	0 (0 to 0)	0.0 (0.0 to 0.0)	0.1 (0.0 to 0.3)	278 (101 to 1,108)
Central Europe, Eastern Europe, and Central Asia	86 (44 to 144)	0.3 (0.2 to 0.5)	125.6 (66.6 to 214.9)	3,820,504 (2,026,582 to 6,534,889)	151 (90 to 229)	0.0 (0.0 to 0.1)	25.5 (16.8 to 37.2)	10,632,367 (7,031,368 to 15,537,020)
Eastern Europe	8 (4 to 13)	0.1 (0.0 to 0.1)	123.9 (71.3 to 204.2)	1,695,395 (976,167 to 2,794,484)	23 (14 to 33)	0.0 (0.0 to 0.0)	23.1 (15.4 to 32.7)	4,907,596 (3,267,953 to 6,934,008)
Belarus	0 (0 to 0)	0.0 (0.0 to 0.0)	119.7 (64.8 to 210.9)	71,455 (38,670 to 125,927)	1 (0 to 1)	0.0 (0.0 to 0.0)	20.7 (13.4 to 29.7)	197,545 (128,260 to 284,380)
Estonia	0 (0 to 0)	0.0 (0.0 to 0.0)	232.4 (128.1 to 375.1)	17,845 (9,834 to 28,804)	0 (0 to 0)	0.0 (0.0 to 0.0)	45.8 (30.0 to 65.4)	60,729 (39,833 to 86,723)
Latvia	0 (0 to 0)	0.0 (0.0 to 0.0)	190.4 (103.2 to 326.0)	24,620 (13,337 to 42,142)	0 (0 to 0)	0.0 (0.0 to 0.0)	34.7 (22.7 to 49.8)	69,622 (45,536 to 99,768)
Lithuania	0 (0 to 0)	0.0 (0.0 to 0.0)	225.8 (122.1 to 390.9)	39,866 (21,554 to 69,039)	0 (0 to 1)	0.0 (0.0 to 0.0)	40.4 (26.7 to 57.4)	117,971 (77,961 to 167,690)

	Under-5				All ages			
**Location**	**Deaths**	**Deaths per 100,000**	**Incidence per 1000**	**Cases**	**Deaths2**	**Deaths per 100,0003**	**Incidence per 10004**	**Cases5**
Moldova	0 (0 to 0)	0.1 (0.0 to 0.2)	105.2 (57.7 to 179.5)	23,460 (12,855 to 40,026)	0 (0 to 1)	0.0 (0.0 to 0.0)	15.6 (10.3 to 22.6)	63,527 (41,856 to 91,840)
Russia	7 (3 to 11)	0.1 (0.0 to 0.1)	114.6 (67.2 to 186.7)	1,132,040 (664,318 to 1,844,758)	18 (11 to 27)	0.0 (0.0 to 0.0)	21.9 (14.6 to 31.0)	3,214,617 (2,142,707 to 4,547,022)
Ukraine	1 (0 to 2)	0.0 (0.0 to 0.1)	148.4 (80.9 to 253.0)	386,124 (210,486 to 658,009)	3 (2 to 5)	0.0 (0.0 to 0.0)	25.9 (17.0 to 36.8)	1,183,503 (778,769 to 1,683,797)
Central Europe	4 (2 to 8)	0.1 (0.0 to 0.1)	213.7 (90.2 to 416.3)	1,220,079 (514,888 to 2,376,811)	40 (22 to 64)	0.0 (0.0 to 0.1)	29.7 (18.0 to 46.7)	3,441,408 (2,088,315 to 5,412,845)
Albania	0 (0 to 0)	0.0 (0.0 to 0.1)	162.7 (62.7 to 336.5)	28,237 (10,875 to 58,401)	0 (0 to 1)	0.0 (0.0 to 0.0)	22.8 (12.8 to 38.4)	65,711 (36,889 to 110,649)
Bosnia and Herzegovina	0 (0 to 0)	0.0 (0.0 to 0.1)	68.0 (27.5 to 134.9)	11,736 (4,752 to 23,289)	0 (0 to 0)	0.0 (0.0 to 0.0)	9.0 (5.1 to 14.5)	34,436 (19,607 to 55,325)
Bulgaria	0 (0 to 1)	0.1 (0.0 to 0.2)	177.3 (68.8 to 367.0)	60,273 (23,377 to 124,761)	1 (1 to 2)	0.0 (0.0 to 0.0)	23.0 (13.6 to 37.3)	166,651 (98,822 to 270,500)
Croatia	0 (0 to 0)	0.0 (0.0 to 0.1)	231.5 (95.9 to 435.5)	46,053 (19,070 to 86,632)	1 (1 to 2)	0.0 (0.0 to 0.0)	28.8 (17.0 to 44.8)	121,601 (72,014 to 189,164)
Czech Republic	0 (0 to 0)	0.0 (0.0 to 0.1)	204.5 (82.7 to 405.3)	115,768 (46,833 to 229,392)	10 (5 to 17)	0.1 (0.0 to 0.2)	32.9 (19.6 to 52.0)	350,670 (208,792 to 553,874)
Hungary	0 (0 to 0)	0.1 (0.0 to 0.1)	172.6 (66.9 to 357.0)	87,828 (34,058 to 181,657)	10 (5 to 16)	0.1 (0.1 to 0.2)	29.2 (17.4 to 45.3)	291,174 (173,712 to 451,623)
Macedonia	0 (0 to 0)	0.1 (0.0 to 0.3)	149.1 (60.3 to 294.2)	16,983 (6,869 to 33,509)	0 (0 to 1)	0.0 (0.0 to 0.0)	21.0 (12.1 to 34.7)	43,682 (25,149 to 72,047)
Montenegro	0 (0 to 0)	0.0 (0.0 to 0.1)	102.6 (40.8 to 207.8)	3,793 (1,511 to 7,683)	0 (0 to 0)	0.0 (0.0 to 0.0)	16.1 (9.2 to 26.5)	10,086 (5,793 to 16,606)
Poland	0 (0 to 1)	0.0 (0.0 to 0.0)	214.5 (83.4 to 442.8)	426,283 (165,733 to 879,865)	9 (4 to 14)	0.0 (0.0 to 0.0)	29.3 (16.9 to 47.6)	1,135,408 (654,563 to 1,846,201)

	Under-5				All ages			
**Location**	**Deaths**	**Deaths per 100,000**	**Incidence per 1000**	**Cases**	**Deaths2**	**Deaths per 100,0003**	**Incidence per 10004**	**Cases5**
Romania	2 (1 to 4)	0.3 (0.1 to 0.5)	314.5 (154.5 to 579.7)	250,694 (123,128 to 462,044)	5 (3 to 8)	0.0 (0.0 to 0.0)	40.4 (26.3 to 60.0)	778,917 (506,325 to 1,157,545)
Serbia	0 (0 to 1)	0.1 (0.0 to 0.1)	187.3 (79.9 to 346.1)	76,865 (32,803 to 142,067)	2 (1 to 4)	0.0 (0.0 to 0.0)	23.5 (14.0 to 37.0)	206,100 (122,657 to 324,203)
Slovakia	0 (0 to 0)	0.1 (0.0 to 0.1)	230.2 (88.7 to 470.0)	68,551 (26,411 to 139,945)	1 (0 to 2)	0.0 (0.0 to 0.0)	31.2 (17.8 to 51.6)	170,325 (97,220 to 282,304)
Slovenia	0 (0 to 0)	0.0 (0.0 to 0.0)	245.9 (94.6 to 492.9)	26,147 (10,057 to 52,407)	0 (0 to 1)	0.0 (0.0 to 0.0)	31.2 (17.9 to 50.3)	64,475 (37,062 to 103,965)
Central Asia	74 (37 to 127)	0.8 (0.4 to 1.3)	82.6 (45.8 to 138.7)	909,028 (504,335 to 1,527,024)	89 (48 to 144)	0.1 (0.1 to 0.2)	25.5 (16.6 to 37.1)	2,286,663 (1,489,518 to 3,330,103)
Armenia	0 (0 to 1)	0.2 (0.1 to 0.3)	115.8 (64.1 to 197.1)	26,042 (14,407 to 44,334)	1 (0 to 1)	0.0 (0.0 to 0.0)	27.3 (18.0 to 39.2)	83,143 (54,766 to 119,427)
Azerbaijan	7 (3 to 15)	0.8 (0.3 to 1.6)	132.1 (72.9 to 224.4)	122,315 (67,552 to 207,861)	8 (4 to 16)	0.1 (0.0 to 0.2)	33.1 (21.6 to 47.7)	324,832 (211,848 to 467,125)
Georgia	0 (0 to 1)	0.1 (0.1 to 0.3)	117.3 (63.5 to 202.2)	32,755 (17,722 to 56,462)	1 (1 to 1)	0.0 (0.0 to 0.0)	22.4 (14.8 to 32.2)	89,363 (58,834 to 128,236)
Kazakhstan	3 (1 to 5)	0.1 (0.1 to 0.3)	79.4 (43.7 to 134.6)	155,199 (85,434 to 262,995)	3 (2 to 6)	0.0 (0.0 to 0.0)	23.2 (14.9 to 33.9)	415,043 (266,985 to 605,368)
Kyrgyzstan	8 (4 to 14)	1.1 (0.5 to 1.9)	80.5 (46.2 to 132.3)	65,313 (37,487 to 107,319)	9 (5 to 15)	0.2 (0.1 to 0.3)	26.3 (17.1 to 38.6)	159,236 (103,533 to 233,321)
Mongolia	0 (0 to 0)	0.0 (0.0 to 0.0)	111.3 (62.6 to 187.4)	41,396 (23,282 to 69,732)	0 (0 to 0)	0.0 (0.0 to 0.0)	29.3 (18.8 to 43.2)	89,105 (56,959 to 131,258)
Tajikistan	45 (18 to 83)	3.9 (1.6 to 7.3)	146.5 (80.8 to 243.3)	160,915 (88,688 to 267,133)	53 (24 to 94)	0.6 (0.3 to 1.1)	41.0 (26.3 to 61.3)	350,199 (224,955 to 524,237)
Turkmenistan	5 (2 to 10)	0.8 (0.3 to 1.7)	154.4 (84.2 to 260.0)	82,774 (45,145 to 139,353)	6 (3 to 10)	0.1 (0.0 to 0.2)	40.7 (26.2 to 59.3)	221,619 (142,693 to 322,531)

Location	Under-5				All ages			
	Deaths	Deaths per 100,0000	Incidence per 1000	Cases	Deaths2	Deaths per 100,0003	Incidence per 10004	Cases5
Uzbekistan	6 (2 to 12)	0.2 (0.1 to 0.4)	46.3 (24.6 to 79.8)	222,601 (118,548 to 383,903)	8 (4 to 14)	0.0 (0.0 to 0.0)	17.4 (11.1 to 25.7)	553,721 (355,033 to 820,753)
Latin America and Caribbean	348 (183 to 564)	0.7 (0.4 to 1.1)	179.2 (96.9 to 300.3)	8,474,935 (4,582,797 to 14,202,885)	1,006 (596 to 1,466)	0.2 (0.1 to 0.3)	44.6 (29.2 to 64.3)	25,630,683 (16,791,287 to 36,938,790)
Central Latin America	150 (77 to 242)	0.7 (0.3 to 1.1)	111.4 (54.9 to 205.4)	2,490,169 (1,227,153 to 4,590,038)	462 (276 to 677)	0.2 (0.1 to 0.3)	29.8 (18.6 to 45.7)	7,578,241 (4,715,595 to 11,602,381)
Colombia	11 (4 to 23)	0.3 (0.1 to 0.6)	170.2 (67.8 to 365.7)	550,713 (219,344 to 1,183,489)	39 (22 to 60)	0.1 (0.0 to 0.1)	46.7 (27.4 to 74.9)	2,252,063 (1,318,572 to 3,609,572)
Costa Rica	0 (0 to 1)	0.1 (0.0 to 0.3)	145.5 (66.0 to 285.7)	47,183 (21,400 to 92,661)	4 (2 to 6)	0.1 (0.0 to 0.1)	46.1 (28.1 to 71.6)	222,937 (135,913 to 346,039)
El Salvador	3 (1 to 6)	0.5 (0.1 to 1.1)	210.6 (65.6 to 472.8)	98,721 (30,757 to 221,690)	11 (5 to 23)	0.2 (0.1 to 0.4)	55.0 (29.0 to 94.6)	335,595 (177,104 to 576,899)
Guatemala	90 (50 to 142)	4.5 (2.6 to 7.2)	437.4 (251.0 to 685.5)	837,627 (480,645 to 1,312,743)	247 (159 to 356)	1.5 (1.0 to 2.2)	125.5 (82.0 to 179.5)	2,067,058 (1,349,910 to 2,956,981)
Honduras	10 (3 to 23)	1.0 (0.3 to 2.4)	194.9 (64.4 to 406.3)	182,669 (60,306 to 380,702)	37 (15 to 69)	0.4 (0.2 to 0.8)	55.3 (28.2 to 97.2)	460,050 (234,137 to 808,813)
Mexico	24 (9 to 46)	0.2 (0.1 to 0.4)	45.3 (21.1 to 86.6)	528,213 (246,501 to 1,010,315)	97 (48 to 152)	0.1 (0.0 to 0.1)	11.5 (7.0 to 17.7)	1,485,401 (895,182 to 2,279,441)
Nicaragua	8 (4 to 14)	1.3 (0.6 to 2.3)	235.4 (130.2 to 402.4)	143,498 (79,372 to 245,257)	18 (10 to 32)	0.3 (0.2 to 0.5)	80.0 (53.0 to 114.2)	492,834 (326,501 to 703,845)
Panama	4 (1 to 7)	1.0 (0.3 to 2.1)	195.6 (62.8 to 429.5)	71,144 (22,852 to 156,207)	7 (4 to 13)	0.2 (0.1 to 0.3)	56.4 (29.4 to 98.0)	224,557 (117,235 to 390,441)
Venezuela	0 (0 to 1)	0.0 (0.0 to 0.0)	3.6 (1.0 to 26.2)	10,117 (2,808 to 74,140)	1 (0 to 1)	0.0 (0.0 to 0.0)	1.2 (0.4 to 5.2)	38,950 (13,409 to 164,334)
Andean Latin America	43 (24 to 73)	0.6 (0.4 to 1.1)	224.4 (126.1 to 367.5)	1,472,041 (826,886 to 2,410,738)	127 (75 to 213)	0.2 (0.1 to 0.4)	81.1 (54.3 to 113.8)	4,849,344 (3,247,750 to 6,800,556)
Bolivia	19 (9 to 34)	1.4 (0.6 to 2.5)	272.2 (144.6 to 489.0)	363,430 (193,080 to 652,909)	48 (25 to 83)	0.4 (0.2 to 0.8)	100.1 (65.1 to 145.5)	1,104,382 (718,448 to 1,605,421)

Location	Under-5				All ages			
	Deaths	Deaths per 100,000	Incidence per 1000	Cases	Deaths2	Deaths per 100,0003	Incidence per 10004	Cases5
Ecuador	10 (5 to 18)	0.5 (0.3 to 1.0)	245.2 (129.4 to 417.9)	414,629 (218,719 to 706,561)	24 (15 to 35)	0.1 (0.1 to 0.2)	80.4 (52.7 to 114.1)	1,323,904 (867,107 to 1,878,829)
Peru	15 (7 to 26)	0.4 (0.2 to 0.7)	196.3 (115.4 to 310.4)	693,772 (407,953 to 1,096,932)	55 (30 to 106)	0.2 (0.1 to 0.3)	75.0 (50.6 to 104.0)	2,420,476 (1,633,608 to 3,357,342)
Caribbean	104 (47 to 203)	2.6 (1.2 to 5.1)	145.4 (80.3 to 246.0)	599,771 (331,223 to 1,015,079)	211 (112 to 359)	0.5 (0.2 to 0.8)	47.2 (31.1 to 67.0)	2,164,245 (1,425,531 to 3,073,148)
Antigua and Barbuda	0 (0 to 0)	0.2 (0.1 to 0.4)	161.7 (85.7 to 283.4)	747 (396 to 1,310)	0 (0 to 0)	0.1 (0.0 to 0.1)	45.1 (29.6 to 64.2)	4,065 (2,665 to 5,784)
The Bahamas	0 (0 to 0)	0.1 (0.0 to 0.3)	138.5 (72.0 to 241.8)	4,424 (2,300 to 7,722)	0 (0 to 0)	0.0 (0.0 to 0.1)	45.9 (29.9 to 66.0)	18,143 (11,825 to 26,094)
Barbados	0 (0 to 0)	0.1 (0.0 to 0.2)	133.1 (69.7 to 238.1)	2,087 (1,094 to 3,736)	0 (0 to 0)	0.1 (0.0 to 0.1)	48.5 (31.8 to 69.3)	13,743 (9,014 to 19,648)
Belize	0 (0 to 0)	0.3 (0.1 to 0.8)	139.6 (77.2 to 240.2)	5,768 (3,190 to 9,921)	0 (0 to 1)	0.1 (0.1 to 0.2)	50.0 (32.1 to 73.0)	18,396 (11,810 to 26,844)
Bermuda	0 (0 to 0)	0.0 (0.0 to 0.1)	149.0 (75.2 to 268.5)	660 (333 to 1,189)	0 (0 to 0)	0.0 (0.0 to 0.0)	48.8 (31.8 to 69.9)	3,431 (2,241 to 4,923)
Cuba	0 (0 to 1)	0.1 (0.0 to 0.1)	90.1 (47.3 to 161.2)	55,599 (29,178 to 99,455)	12 (7 to 18)	0.1 (0.1 to 0.2)	34.8 (22.8 to 49.0)	397,204 (260,654 to 560,167)
Dominica	0 (0 to 0)	0.2 (0.1 to 0.5)	131.2 (69.4 to 232.7)	709 (375 to 1,258)	0 (0 to 0)	0.1 (0.0 to 0.1)	46.7 (30.6 to 67.1)	3,476 (2,274 to 4,993)
Dominican Republic	8 (3 to 14)	0.8 (0.4 to 1.5)	154.8 (82.6 to 269.9)	157,582 (84,084 to 274,664)	20 (10 to 36)	0.2 (0.1 to 0.3)	51.4 (33.2 to 74.0)	545,659 (352,591 to 785,052)
Grenada	0 (0 to 0)	0.2 (0.1 to 0.3)	132.1 (67.4 to 235.1)	1,576 (804 to 2,805)	0 (0 to 0)	0.1 (0.0 to 0.1)	68.6 (44.5 to 99.0)	7,506 (4,863 to 10,830)
Guyana	1 (0 to 1)	1.0 (0.4 to 1.8)	99.0 (54.4 to 168.6)	7,070 (3,883 to 12,037)	2 (1 to 3)	0.3 (0.2 to 0.4)	39.1 (25.6 to 55.9)	30,087 (19,710 to 43,085)

	Under-5				All ages			
**Location**	**Deaths**	**Deaths per 100,0000**	**Incidence per 1000**	**Cases**	**Deaths2**	**Deaths per 100,0003**	**Incidence per 10004**	**Cases5**
Haiti	93 (40 to 184)	6.1 (2.6 to 12.1)	197.5 (111.2 to 330.6)	298,625 (168,171 to 499,847)	167 (84 to 299)	1.5 (0.8 to 2.7)	70.7 (45.6 to 102.1)	786,329 (507,916 to 1,136,057)
Jamaica	1 (0 to 2)	0.3 (0.1 to 0.6)	84.2 (45.4 to 144.3)	22,540 (12,138 to 38,623)	3 (1 to 7)	0.1 (0.0 to 0.2)	30.4 (20.0 to 43.3)	87,062 (57,456 to 124,306)
Puerto Rico	0 (0 to 0)	0.1 (0.0 to 0.1)	108.9 (56.7 to 195.2)	26,170 (13,628 to 46,926)	3 (2 to 5)	0.1 (0.1 to 0.1)	42.6 (28.1 to 60.2)	157,829 (104,003 to 223,048)
Saint Lucia	0 (0 to 0)	0.2 (0.1 to 0.4)	190.4 (101.9 to 330.1)	1,906 (1,020 to 3,304)	0 (0 to 0)	0.1 (0.0 to 0.1)	72.9 (47.8 to 103.3)	13,310 (8,736 to 18,862)
Saint Vincent and the Grenadines	0 (0 to 0)	0.3 (0.1 to 0.7)	114.7 (61.6 to 200.5)	1,093 (586 to 1,910)	0 (0 to 0)	0.1 (0.1 to 0.2)	45.2 (29.9 to 64.9)	5,014 (3,316 to 7,199)
Suriname	1 (0 to 1)	1.2 (0.5 to 2.5)	121.0 (65.3 to 211.1)	6,384 (3,446 to 11,139)	2 (1 to 3)	0.3 (0.1 to 0.5)	47.4 (31.0 to 67.9)	26,186 (17,118 to 37,489)
Trinidad and Tobago	0 (0 to 0)	0.2 (0.1 to 0.5)	71.0 (37.8 to 126.8)	6,305 (3,354 to 11,262)	1 (1 to 2)	0.1 (0.0 to 0.1)	28.3 (18.7 to 40.7)	38,406 (25,463 to 55,273)
Virgin Islands, U.S.	0 (0 to 0)	0.0 (0.0 to 0.1)	142.3 (73.2 to 258.7)	778 (400 to 1,413)	0 (0 to 0)	0.1 (0.0 to 0.1)	49.8 (32.7 to 71.3)	5,216 (3,426 to 7,474)
Tropical Latin America	51 (24 to 89)	0.3 (0.2 to 0.6)	273.6 (149.7 to 453.2)	3,901,845 (2,134,792 to 6,462,186)	206 (120 to 306)	0.1 (0.1 to 0.1)	51.4 (33.3 to 74.2)	11,042,389 (7,153,152 to 15,919,339)
Brazil	49 (23 to 86)	0.3 (0.1 to 0.6)	267.5 (147.1 to 442.7)	3,730,923 (2,051,899 to 6,174,146)	198 (116 to 296)	0.1 (0.1 to 0.1)	50.4 (32.7 to 72.7)	10,498,085 (6,818,983 to 15,141,635)
Paraguay	2 (1 to 4)	0.4 (0.1 to 0.7)	546.5 (288.0 to 899.7)	170,934 (90,078 to 281,405)	7 (4 to 13)	0.1 (0.1 to 0.2)	85.4 (54.1 to 125.0)	543,930 (344,586 to 796,053)
Southeast Asia, East Asia, and Oceania	430 (181 to 805)	0.3 (0.1 to 0.7)	41.0 (18.5 to 80.8)	5,169,128 (2,329,592 to 10,200,694)	1,828 (896 to 3,120)	0.1 (0.0 to 0.1)	10.5 (6.4 to 16.1)	21,839,768 (13,255,008 to 33,500,640)
East Asia	1 (0 to 1)	0.0 (0.0 to 0.0)	0.3 (0.2 to 0.4)	17,775 (11,027 to 27,005)	3 (2 to 5)	0.0 (0.0 to 0.0)	0.1 (0.1 to 0.1)	151,553 (102,926 to 200,656)
China	1 (0 to 1)	0.0 (0.0 to 0.0)	0.2 (0.1 to 0.4)	14,630 (9,066 to 22,594)	3 (2 to 5)	0.0 (0.0 to 0.0)	0.1 (0.1 to 0.1)	135,183 (91,355 to 179,530)

	Under-5				All ages			
Location	Deaths	Deaths per 100,000	Incidence per 1000	Cases	Deaths2	Deaths per 100,0003	Incidence per 10004	Cases5
North Korea	0 (0 to 0)	0.0 (0.0 to 0.0)	0.8 (0.5 to 1.1)	2,593 (1,653 to 3,695)	0 (0 to 1)	0.0 (0.0 to 0.0)	0.4 (0.3 to 0.5)	10,766 (7,372 to 14,152)
Taiwan	0 (0 to 0)	0.0 (0.0 to 0.0)	0.5 (0.3 to 0.8)	555 (338 to 823)	0 (0 to 0)	0.0 (0.0 to 0.0)	0.2 (0.2 to 0.3)	5,615 (3,791 to 7,386)
Southeast Asia	390 (158 to 747)	0.7 (0.3 to 1.3)	82.0 (35.6 to 163.5)	4,820,721 (2,091,257 to 9,610,935)	1,632 (777 to 2,839)	0.2 (0.1 to 0.4)	30.8 (18.6 to 47.7)	20,197,076 (12,235,267 to 31,296,874)
Cambodia	16 (7 to 32)	0.9 (0.4 to 1.7)	187.4 (100.0 to 311.3)	343,475 (183,293 to 570,503)	83 (42 to 145)	0.5 (0.3 to 0.9)	76.1 (49.6 to 108.7)	1,209,875 (787,772 to 1,727,174)
Indonesia	62 (11 to 143)	0.3 (0.1 to 0.6)	37.5 (15.0 to 77.9)	901,187 (361,635 to 1,872,153)	403 (101 to 900)	0.2 (0.0 to 0.3)	13.0 (6.7 to 21.4)	3,379,119 (1,729,880 to 5,559,701)
Laos	84 (31 to 185)	7.4 (2.8 to 16.3)	321.0 (162.6 to 558.2)	346,531 (175,558 to 602,525)	127 (62 to 241)	1.8 (0.9 to 3.3)	125.3 (79.6 to 187.7)	897,311 (569,958 to 1,343,999)
Malaysia	3 (1 to 5)	0.1 (0.0 to 0.2)	124.1 (39.2 to 282.6)	322,905 (102,109 to 735,512)	25 (11 to 48)	0.1 (0.0 to 0.2)	51.8 (27.1 to 89.4)	1,593,486 (832,863 to 2,753,963)
Maldives	0 (0 to 0)	0.1 (0.0 to 0.2)	78.7 (26.6 to 172.3)	2,899 (979 to 6,349)	0 (0 to 0)	0.0 (0.0 to 0.1)	37.2 (19.6 to 62.7)	13,702 (7,235 to 23,107)
Mauritius	0 (0 to 0)	0.3 (0.1 to 0.6)	105.3 (36.5 to 233.6)	6,983 (2,418 to 15,491)	1 (1 to 2)	0.1 (0.1 to 0.2)	45.1 (24.3 to 76.6)	57,478 (30,947 to 97,595)
Myanmar	61 (19 to 137)	1.3 (0.4 to 2.9)	100.6 (34.3 to 214.6)	628,952 (214,604 to 1,342,034)	365 (158 to 695)	0.7 (0.3 to 1.3)	52.0 (28.4 to 86.4)	2,916,386 (1,596,251 to 4,850,291)
Philippines	148 (48 to 310)	1.3 (0.4 to 2.7)	116.2 (40.6 to 244.9)	1,344,610 (469,726 to 2,832,656)	362 (184 to 604)	0.4 (0.2 to 0.6)	42.9 (22.7 to 73.6)	4,391,040 (2,321,949 to 7,539,892)
Sri Lanka	1 (0 to 2)	0.1 (0.0 to 0.2)	114.3 (38.6 to 252.5)	159,150 (53,815 to 351,635)	28 (10 to 61)	0.1 (0.0 to 0.3)	42.1 (23.2 to 69.5)	866,474 (477,930 to 1,430,876)
Seychelles	0 (0 to 0)	0.1 (0.0 to 0.2)	116.3 (38.7 to 256.7)	977 (325 to 2,156)	0 (0 to 0)	0.1 (0.0 to 0.2)	45.6 (24.9 to 75.9)	4,426 (2,420 to 7,369)
Thailand	2 (1 to 5)	0.1 (0.0 to 0.2)	85.1 (45.1 to 152.8)	212,912 (112,745 to 382,110)	156 (71 to 318)	0.2 (0.1 to 0.5)	31.4 (20.3 to 44.5)	2,099,689 (1,357,975 to 2,979,165)

	Under-5				All ages			
**Location**	**Deaths**	**Deaths per 100,0003**	**Incidence per 1000**	**Cases**	**Deaths2**	**Deaths per 100,0003**	**Incidence per 10004**	**Cases5**
Timor-Leste	10 (3 to 23)	6.0 (1.9 to 13.8)	220.3 (76.6 to 474.7)	36,219 (12,591 to 78,049)	14 (5 to 28)	1.2 (0.4 to 2.4)	86.8 (44.5 to 153.6)	100,538 (51,554 to 177,860)
Vietnam	3 (1 to 6)	0.0 (0.0 to 0.1)	71.5 (34.0 to 133.1)	511,582 (243,307 to 952,179)	69 (23 to 154)	0.1 (0.0 to 0.2)	28.3 (17.7 to 42.1)	2,654,356 (1,660,758 to 3,949,033)
Oceania	39 (17 to 78)	2.8 (1.2 to 5.5)	243.2 (135.9 to 412.8)	335,773 (187,598 to 569,904)	193 (100 to 325)	1.7 (0.9 to 2.9)	131.1 (85.8 to 185.6)	1,464,861 (959,231 to 2,073,550)
American Samoa	0 (0 to 0)	0.2 (0.1 to 0.3)	194.2 (105.2 to 337.0)	1,451 (787 to 2,519)	0 (0 to 0)	0.1 (0.1 to 0.2)	117.6 (76.1 to 171.2)	9,492 (6,139 to 13,815)
Federated States of Micronesia	0 (0 to 0)	0.3 (0.1 to 0.7)	162.8 (88.9 to 280.9)	1,609 (879 to 2,776)	0 (0 to 1)	0.3 (0.2 to 0.7)	107.8 (69.6 to 155.9)	11,121 (7,179 to 16,086)
Fiji	1 (1 to 3)	2.6 (1.1 to 5.2)	195.7 (109.1 to 335.5)	10,660 (5,942 to 18,272)	6 (3 to 10)	0.7 (0.4 to 1.2)	96.9 (63.7 to 136.0)	83,828 (55,111 to 117,681)
Guam	0 (0 to 0)	0.1 (0.0 to 0.1)	202.4 (108.6 to 356.2)	3,536 (1,898 to 6,224)	0 (0 to 0)	0.1 (0.0 to 0.1)	119.6 (78.6 to 166.7)	20,957 (13,768 to 29,193)
Kiribati	1 (0 to 1)	4.9 (1.9 to 10.7)	247.3 (136.1 to 428.2)	3,361 (1,849 to 5,820)	3 (1 to 5)	2.4 (1.1 to 4.3)	128.0 (84.7 to 181.0)	14,528 (9,615 to 20,540)
Marshall Islands	0 (0 to 0)	0.5 (0.2 to 1.1)	186.6 (102.7 to 317.5)	1,834 (1,009 to 3,120)	0 (0 to 0)	0.2 (0.1 to 0.4)	111.3 (72.6 to 159.1)	8,263 (5,394 to 11,815)
Northern Mariana Islands	0 (0 to 0)	0.0 (0.0 to 0.1)	275.1 (145.1 to 503.7)	4,262 (2,248 to 7,805)	0 (0 to 0)	0.0 (0.0 to 0.1)	124.9 (81.4 to 177.4)	15,882 (10,347 to 22,553)
Papua New Guinea	34 (13 to 71)	3.2 (1.2 to 6.6)	258.2 (142.3 to 438.6)	268,996 (148,204 to 456,908)	173 (87 to 293)	2.2 (1.1 to 3.7)	145.0 (94.4 to 206.1)	1,133,144 (737,724 to 1,610,702)
Samoa	0 (0 to 0)	0.1 (0.0 to 0.3)	208.0 (112.4 to 365.6)	5,745 (3,104 to 10,099)	0 (0 to 1)	0.2 (0.1 to 0.5)	127.5 (82.9 to 182.6)	25,337 (16,479 to 36,281)
Solomon Islands	2 (1 to 4)	2.3 (1.0 to 4.4)	255.1 (140.4 to 430.1)	20,912 (11,512 to 35,262)	6 (3 to 12)	1.1 (0.5 to 1.9)	141.7 (93.3 to 201.8)	84,314 (55,540 to 120,123)

Location	Under-5 Deaths	Under-5 Deaths per 100,0003	Under-5 Incidence per 1000	Under-5 Cases	All ages Deaths2	All ages Deaths per 100,0003	All ages Incidence per 10004	All ages Cases5
Tonga	0 (0 to 0)	0.4 (0.2 to 0.9)	191.9 (106.9 to 326.7)	2,627 (1,464 to 4,472)	0 (0 to 1)	0.3 (0.2 to 0.6)	125.3 (81.8 to 180.6)	13,528 (8,832 to 19,492)
Vanuatu	1 (0 to 2)	1.9 (0.7 to 3.8)	266.9 (148.5 to 448.1)	10,779 (5,997 to 18,095)	3 (2 to 6)	1.1 (0.6 to 2.1)	151.7 (100.7 to 214.8)	41,938 (27,842 to 59,370)
North Africa and Middle East	2,076 (1,063 to 3,532)	3.3 (1.7 to 5.6)	266.0 (132.6 to 479.9)	16,894,231 (8,422,229 to 30,482,176)	2,815 (1,578 to 4,584)	0.5 (0.3 to 0.8)	71.2 (43.2 to 110.4)	40,936,712 (24,862,627 to 63,496,927)
North Africa and Middle East	2,076 (1,063 to 3,532)	3.3 (1.7 to 5.6)	266.0 (132.6 to 479.9)	16,894,231 (8,422,229 to 30,482,176)	2,815 (1,578 to 4,584)	0.5 (0.3 to 0.8)	71.2 (43.2 to 110.4)	40,936,712 (24,862,627 to 63,496,927)
Afghanistan	281 (100 to 639)	5.7 (2.0 to 12.9)	354.4 (136.0 to 755.8)	1,680,416 (644,767 to 3,583,935)	317 (125 to 671)	0.9 (0.4 to 2.0)	105.3 (53.1 to 190.0)	3,495,984 (1,763,786 to 6,304,186)
Algeria	14 (4 to 33)	0.3 (0.1 to 0.7)	153.2 (59.5 to 312.1)	779,442 (303,010 to 1,588,337)	44 (21 to 83)	0.1 (0.1 to 0.2)	50.0 (27.3 to 84.5)	2,040,860 (1,115,067 to 3,450,675)
Bahrain	0 (0 to 0)	0.1 (0.0 to 0.1)	180.7 (65.8 to 412.7)	17,424 (6,347 to 39,801)	1 (0 to 1)	0.0 (0.0 to 0.1)	42.1 (22.7 to 71.6)	58,370 (31,396 to 99,203)
Egypt	733 (334 to 1,373)	6.7 (3.1 to 12.6)	290.4 (170.9 to 453.9)	3,472,604 (2,043,908 to 5,427,409)	870 (441 to 1,546)	0.9 (0.5 to 1.7)	84.6 (56.2 to 120.6)	7,845,487 (5,210,393 to 11,183,060)
Iran	14 (4 to 33)	0.2 (0.1 to 0.4)	170.2 (90.4 to 302.0)	1,207,186 (641,482 to 2,141,583)	48 (24 to 89)	0.1 (0.0 to 0.1)	41.9 (26.6 to 62.2)	3,368,922 (2,139,297 to 4,994,494)
Iraq	104 (32 to 234)	1.3 (0.4 to 3.0)	236.8 (88.3 to 496.5)	1,753,217 (653,556 to 3,676,760)	150 (67 to 285)	0.4 (0.2 to 0.7)	78.9 (39.1 to 141.1)	3,085,377 (1,528,622 to 5,517,535)
Jordan	2 (1 to 3)	0.2 (0.1 to 0.3)	167.4 (63.1 to 357.6)	160,676 (60,524 to 343,188)	3 (2 to 6)	0.0 (0.0 to 0.1)	49.9 (26.0 to 88.5)	385,312 (200,850 to 683,257)
Kuwait	0 (0 to 0)	0.0 (0.0 to 0.1)	36.7 (10.0 to 83.7)	24,433 (6,659 to 55,709)	0 (0 to 0)	0.0 (0.0 to 0.0)	17.6 (8.6 to 30.7)	75,909 (37,232 to 132,531)
Lebanon	1 (0 to 2)	0.2 (0.1 to 0.5)	234.0 (89.4 to 495.6)	70,528 (26,963 to 149,404)	4 (2 to 10)	0.1 (0.0 to 0.2)	50.8 (28.2 to 84.0)	295,317 (163,799 to 487,877)
Libya	0 (0 to 0)	0.0 (0.0 to 0.1)	35.4 (3.8 to 87.6)	17,412 (1,867 to 43,074)	0 (0 to 1)	0.0 (0.0 to 0.0)	9.2 (2.5 to 19.1)	56,800 (15,719 to 118,205)

Location	Under-5				All ages			
	Deaths	Deaths per 100,000	Incidence per 1000	Cases	Deaths2	Deaths per 100,0003	Incidence per 10004	Cases5
Morocco	60 (20 to 126)	2.6 (0.9 to 5.5)	217.7 (82.9 to 472.5)	488,618 (186,071 to 1,060,445)	91 (41 to 172)	0.3 (0.1 to 0.5)	47.6 (26.0 to 79.8)	1,599,065 (875,261 to 2,683,778)
Palestine	2 (1 to 5)	0.2 (0.1 to 0.4)	216.2 (79.6 to 463.2)	231,311 (85,091 to 495,438)	4 (2 to 7)	0.1 (0.0 to 0.1)	78.3 (38.3 to 140.6)	403,208 (197,242 to 723,728)
Oman	0 (0 to 0)	0.1 (0.0 to 0.1)	207.7 (75.5 to 460.8)	89,415 (32,516 to 198,371)	1 (1 to 2)	0.0 (0.0 to 0.0)	53.5 (28.3 to 91.6)	250,423 (132,445 to 429,104)
Qatar	0 (0 to 0)	0.1 (0.0 to 0.2)	108.5 (40.7 to 242.4)	13,747 (5,159 to 30,717)	0 (0 to 0)	0.0 (0.0 to 0.0)	22.1 (12.1 to 37.0)	50,348 (27,603 to 84,442)
Saudi Arabia	3 (1 to 5)	0.1 (0.1 to 0.2)	143.4 (71.0 to 264.4)	365,616 (181,132 to 674,130)	18 (9 to 31)	0.1 (0.0 to 0.1)	44.1 (28.2 to 65.9)	1,392,819 (889,405 to 2,079,769)
Sudan	456 (155 to 1,014)	10.9 (3.7 to 24.2)	712.3 (377.9 to 1,234.5)	2,629,607 (1,395,303 to 4,557,559)	764 (345 to 1,420)	1.9 (0.9 to 3.6)	177.3 (112.1 to 261.3)	6,885,430 (4,354,438 to 10,145,311)
Syria	2 (1 to 5)	0.1 (0.0 to 0.3)	152.9 (56.5 to 343.3)	296,936 (109,729 to 666,783)	4 (2 to 8)	0.0 (0.0 to 0.0)	49.8 (25.5 to 89.4)	916,501 (468,384 to 1,644,147)
Tunisia	2 (1 to 4)	0.2 (0.1 to 0.5)	250.3 (136.3 to 430.8)	232,256 (126,451 to 399,807)	23 (10 to 56)	0.2 (0.1 to 0.5)	77.6 (52.2 to 108.6)	877,236 (590,078 to 1,227,414)
Turkey	14 (5 to 31)	0.2 (0.1 to 0.5)	193.3 (71.6 to 425.1)	1,204,860 (446,548 to 2,649,773)	41 (19 to 78)	0.1 (0.0 to 0.1)	50.4 (27.4 to 85.2)	4,004,786 (2,171,585 to 6,767,110)
United Arab Emirates	0 (0 to 1)	0.0 (0.0 to 0.1)	178.8 (65.0 to 401.9)	166,439 (60,531 to 374,225)	2 (1 to 5)	0.0 (0.0 to 0.0)	43.0 (23.4 to 73.2)	417,310 (227,271 to 710,412)
Yemen	387 (117 to 854)	8.4 (2.5 to 18.5)	433.4 (165.7 to 904.1)	1,957,814 (748,680 to 4,084,166)	430 (155 to 919)	1.5 (0.6 to 3.3)	118.8 (58.7 to 211.4)	3,329,602 (1,645,266 to 5,926,835)
South Asia	4,482 (2,318 to 7,382)	2.9 (1.5 to 4.8)	99.8 (58.7 to 161.4)	15,952,557 (9,373,596 to 25,785,888)	22,942 (10,613 to 42,231)	1.3 (0.6 to 2.5)	40.6 (27.1 to 57.1)	69,281,143 (46,178,724 to 97,325,901)
South Asia	4,482 (2,318 to 7,382)	2.9 (1.5 to 4.8)	99.8 (58.7 to 161.4)	15,952,557 (9,373,596 to 25,785,888)	22,942 (10,613 to 42,231)	1.3 (0.6 to 2.5)	40.6 (27.1 to 57.1)	69,281,143 (46,178,724 to 97,325,901)

	Under-5				All ages			
Location	Deaths	Deaths per 100,000	Incidence per 1000	Cases	Deaths2	Deaths per 100,0003	Incidence per 10004	Cases5
Bangladesh	131 (61 to 233)	0.9 (0.4 to 1.6)	90.3 (52.7 to 149.1)	1,347,289 (786,532 to 2,224,726)	1,052 (494 to 2,167)	0.7 (0.3 to 1.3)	39.8 (26.6 to 55.5)	6,465,361 (4,319,184 to 9,019,507)
Bhutan	1 (0 to 1)	0.8 (0.3 to 1.8)	345.3 (163.8 to 604.5)	26,114 (12,391 to 45,717)	3 (1 to 7)	0.4 (0.1 to 0.9)	132.4 (84.0 to 196.2)	105,309 (66,798 to 156,086)
India	1,895 (921 to 3,211)	1.7 (0.8 to 2.9)	57.9 (31.4 to 100.7)	6,728,430 (3,650,323 to 11,700,779)	17,506 (7,353 to 32,253)	1.3 (0.6 to 2.5)	31.5 (20.6 to 45.2)	41,599,405 (27,245,555 to 59,623,971)
Nepal	25 (8 to 55)	0.6 (0.2 to 1.4)	104.6 (53.0 to 177.7)	440,377 (223,177 to 747,790)	172 (57 to 358)	0.6 (0.2 to 1.2)	39.0 (23.8 to 58.3)	1,180,185 (720,419 to 1,767,715)
Pakistan	2,430 (1,255 to 4,114)	10.5 (5.4 to 17.8)	307.7 (184.7 to 480.9)	7,521,272 (4,514,107 to 11,754,001)	4,208 (2,294 to 7,270)	2.2 (1.2 to 3.8)	102.3 (70.1 to 139.6)	19,673,378 (13,478,312 to 26,851,286)
Sub-Saharan Africa	11,244 (5,266 to 19,427)	7.2 (3.4 to 12.4)	153.2 (78.0 to 274.1)	24,205,390 (12,328,386 to 43,295,519)	22,261 (12,092 to 35,882)	2.3 (1.2 to 3.7)	52.8 (33.0 to 80.5)	51,845,859 (32,391,550 to 79,012,688)
Southern Sub-Saharan Africa	212 (80 to 387)	2.5 (0.9 to 4.5)	71.3 (36.6 to 123.1)	645,778 (331,917 to 1,116,070)	379 (142 to 666)	0.5 (0.2 to 0.9)	24.0 (14.0 to 37.0)	1,857,703 (1,081,496 to 2,868,515)
Botswana	4 (1 to 9)	1.5 (0.5 to 3.2)	51.4 (23.7 to 97.5)	13,973 (6,439 to 26,534)	9 (3 to 17)	0.4 (0.1 to 0.7)	15.5 (8.6 to 24.7)	35,750 (19,789 to 56,839)
Lesotho	18 (6 to 35)	7.1 (2.5 to 13.6)	81.8 (39.3 to 146.6)	20,646 (9,932 to 37,020)	32 (11 to 60)	1.5 (0.5 to 2.8)	23.8 (13.1 to 37.9)	50,712 (27,962 to 80,691)
Namibia	12 (4 to 23)	3.6 (1.1 to 7.0)	78.9 (36.4 to 144.8)	26,876 (12,395 to 49,337)	17 (6 to 32)	0.7 (0.2 to 1.3)	22.2 (12.3 to 35.6)	55,617 (30,931 to 89,194)
South Africa	54 (20 to 105)	1.1 (0.4 to 2.1)	70.2 (35.5 to 122.3)	379,195 (191,916 to 661,071)	160 (54 to 299)	0.3 (0.1 to 0.6)	24.8 (14.6 to 38.2)	1,319,759 (775,717 to 2,029,738)
Swaziland	11 (4 to 21)	5.1 (1.7 to 10.0)	75.1 (35.4 to 141.6)	15,355 (7,234 to 28,956)	14 (5 to 28)	1.1 (0.4 to 2.1)	23.9 (13.2 to 38.6)	31,886 (17,665 to 51,541)
Zimbabwe	112 (42 to 213)	4.4 (1.6 to 8.4)	73.3 (35.5 to 129.6)	189,702 (91,899 to 335,588)	148 (55 to 268)	0.9 (0.3 to 1.7)	22.6 (12.7 to 35.9)	362,485 (203,182 to 575,451)

	Under-5				All ages			
**Location**	**Deaths**	**Deaths per 100,000**	**Incidence per 1000**	**Cases**	**Deaths2**	**Deaths per 100,0003**	**Incidence per 10004**	**Cases5**
Western Sub-Saharan Africa	5,197 (2,032 to 9,574)	8.0 (3.1 to 14.8)	106.4 (47.5 to 198.0)	6,950,968 (3,099,062 to 12,929,044)	6,487 (2,746 to 11,454)	1.6 (0.7 to 2.9)	29.0 (15.7 to 48.6)	11,577,006 (6,265,456 to 19,407,424)
Benin	183 (50 to 406)	9.6 (2.6 to 21.3)	98.5 (33.5 to 221.3)	192,367 (65,357 to 432,232)	232 (75 to 473)	2.0 (0.7 to 4.2)	29.7 (14.0 to 54.5)	338,188 (159,131 to 620,915)
Burkina Faso	63 (6 to 161)	2.0 (0.2 to 5.0)	56.6 (13.9 to 125.7)	182,149 (44,901 to 404,511)	85 (11 to 203)	0.5 (0.1 to 1.1)	14.8 (4.6 to 30.2)	276,119 (86,720 to 563,787)
Cameroon	159 (40 to 361)	4.1 (1.0 to 9.3)	132.1 (43.1 to 296.8)	519,571 (169,620 to 1,167,097)	249 (82 to 503)	1.0 (0.3 to 2.1)	35.7 (16.3 to 66.8)	857,817 (392,243 to 1,607,965)
Cape Verde	0 (0 to 1)	0.4 (0.1 to 0.8)	72.3 (23.0 to 168.5)	5,377 (1,712 to 12,524)	1 (0 to 1)	0.1 (0.0 to 0.3)	22.3 (10.7 to 40.4)	12,200 (5,854 to 22,134)
Chad	437 (117 to 967)	16.5 (4.4 to 36.6)	194.5 (64.0 to 421.0)	501,419 (164,973 to 1,085,439)	517 (171 to 1,078)	3.6 (1.2 to 7.5)	52.7 (23.5 to 98.9)	757,120 (338,346 to 1,420,917)
Cote d'Ivoire	314 (83 to 688)	8.8 (2.3 to 19.3)	139.7 (46.8 to 311.9)	488,608 (163,742 to 1,090,711)	383 (126 to 776)	1.7 (0.5 to 3.4)	35.3 (16.3 to 64.9)	811,090 (375,258 to 1,492,245)
The Gambia	36 (20 to 62)	9.9 (5.3 to 16.8)	296.8 (170.8 to 482.9)	108,988 (62,730 to 177,296)	53 (30 to 86)	2.6 (1.5 to 4.2)	94.3 (62.1 to 135.3)	192,865 (127,111 to 276,716)
Ghana	24 (4 to 56)	0.6 (0.1 to 1.3)	51.1 (15.8 to 106.7)	236,078 (72,708 to 492,684)	43 (9 to 97)	0.2 (0.0 to 0.3)	14.0 (5.8 to 25.3)	400,007 (164,594 to 720,976)
Guinea	85 (23 to 204)	4.2 (1.1 to 10.0)	134.1 (44.0 to 295.8)	268,317 (88,154 to 592,087)	135 (46 to 277)	1.0 (0.4 to 2.2)	36.2 (16.9 to 66.0)	465,293 (217,527 to 848,638)
Guinea-Bissau	32 (16 to 57)	10.4 (5.1 to 18.4)	200.2 (104.7 to 355.8)	61,171 (31,987 to 108,707)	56 (30 to 96)	2.9 (1.6 to 5.0)	63.4 (40.6 to 94.6)	120,467 (77,127 to 179,732)
Liberia	68 (18 to 147)	9.6 (2.6 to 20.7)	163.4 (54.1 to 361.7)	116,028 (38,373 to 256,759)	94 (33 to 180)	2.0 (0.7 to 3.9)	43.6 (20.2 to 79.4)	201,221 (93,018 to 366,273)
Mali	446 (209 to 818)	14.2 (6.6 to 26.0)	142.0 (79.2 to 240.5)	437,424 (244,033 to 740,952)	596 (301 to 1,026)	3.3 (1.7 to 5.7)	43.4 (27.5 to 66.2)	776,976 (491,135 to 1,183,344)

	Under-5				All ages			
Location	Deaths	Deaths per 100,0000	Incidence per 1000	Cases	Deaths2	Deaths per 100,0003	Incidence per 10004	Cases5
Mauritania	19 (5 to 45)	3.8 (1.0 to 8.8)	127.0 (43.0 to 275.4)	63,367 (21,440 to 137,473)	34 (11 to 69)	0.8 (0.3 to 1.7)	31.4 (15.1 to 56.2)	127,449 (61,096 to 227,946)
Niger	467 (111 to 1,053)	12.7 (3.0 to 28.6)	133.4 (45.3 to 299.7)	494,787 (168,161 to 1,111,473)	582 (181 to 1,201)	2.9 (0.9 to 6.0)	41.4 (19.3 to 76.4)	832,417 (387,605 to 1,536,026)
Nigeria	2,705 (1,063 to 5,077)	9.2 (3.6 to 17.2)	100.0 (50.0 to 179.4)	3,002,476 (1,500,643 to 5,386,642)	3,200 (1,315 to 5,801)	1.7 (0.7 to 3.1)	26.5 (14.8 to 42.3)	4,911,071 (2,743,968 to 7,832,774)
Sao Tome and Principe	1 (0 to 1)	1.7 (0.4 to 4.0)	114.4 (37.4 to 255.3)	3,955 (1,294 to 8,823)	1 (0 to 2)	0.4 (0.1 to 0.8)	32.7 (15.0 to 61.1)	6,509 (2,976 to 12,151)
Senegal	3 (1 to 18)	0.1 (0.0 to 0.7)	7.6 (0.9 to 36.2)	19,585 (2,348 to 92,737)	5 (2 to 21)	0.0 (0.0 to 0.1)	1.7 (0.4 to 6.4)	25,797 (6,014 to 99,610)
Sierra Leone	120 (35 to 259)	11.5 (3.4 to 24.9)	116.9 (39.2 to 264.9)	121,684 (40,827 to 275,652)	159 (56 to 327)	2.4 (0.8 to 4.9)	33.2 (15.5 to 60.9)	219,943 (102,502 to 403,555)
Togo	32 (8 to 71)	2.9 (0.8 to 6.5)	117.3 (39.3 to 259.4)	128,156 (42,980 to 283,439)	62 (22 to 122)	0.8 (0.3 to 1.6)	32.5 (15.3 to 58.8)	240,864 (113,290 to 435,584)
Eastern Sub-Saharan Africa	5,485 (2,889 to 8,941)	8.8 (4.6 to 14.3)	243.1 (126.8 to 426.8)	15,163,112 (7,912,373 to 26,621,991)	14,832 (8,531 to 23,472)	3.8 (2.2 to 6.1)	93.4 (60.1 to 137.6)	36,127,390 (23,237,093 to 53,252,943)
Burundi	585 (258 to 1,084)	27.3 (12.1 to 50.6)	320.4 (161.3 to 573.5)	677,712 (341,300 to 1,213,237)	912 (491 to 1,517)	7.9 (4.2 to 13.1)	114.7 (71.4 to 175.2)	1,323,831 (824,032 to 2,022,778)
Comoros	5 (2 to 10)	5.4 (2.2 to 11.2)	252.3 (123.7 to 465.3)	24,523 (12,022 to 45,229)	24 (12 to 45)	3.1 (1.5 to 5.7)	98.1 (62.7 to 146.5)	76,808 (49,118 to 114,698)
Djibouti	6 (2 to 14)	3.5 (1.3 to 7.9)	242.8 (114.9 to 475.1)	30,436 (14,401 to 59,554)	20 (10 to 38)	2.0 (1.0 to 3.9)	75.3 (47.6 to 115.9)	69,441 (43,911 to 106,863)
Eritrea	94 (40 to 188)	11.9 (5.0 to 23.6)	276.0 (132.6 to 528.8)	207,075 (99,486 to 396,786)	212 (116 to 375)	4.0 (2.2 to 7.0)	95.8 (60.1 to 146.3)	506,531 (317,838 to 773,643)
Ethiopia	1,105 (501 to 2,021)	7.2 (3.3 to 13.2)	255.5 (129.6 to 449.4)	3,775,174 (1,914,062 to 6,638,599)	3,506 (1,893 to 5,887)	3.4 (1.9 to 5.8)	93.3 (59.4 to 140.5)	9,496,981 (6,044,577 to 14,303,318)
Kenya	300 (163 to 511)	4.6 (2.5 to 7.8)	130.1 (72.4 to 220.3)	857,013 (477,135 to 1,451,204)	1,853 (961 to 3,295)	4.0 (2.1 to 7.1)	63.8 (42.7 to 89.7)	2,971,849 (1,990,863 to 4,179,743)

	Under-5				All ages			
**Location**	**Deaths**	**Deaths per 100,0000**	**Incidence per 1000**	**Cases**	**Deaths2**	**Deaths per 100,0003**	**Incidence per 10004**	**Cases5**
Madagascar	919 (404 to 1,752)	23.6 (10.4 to 45.1)	305.8 (148.2 to 571.2)	1,181,302 (572,618 to 2,206,485)	1,505 (786 to 2,529)	6.0 (3.2 to 10.1)	98.7 (61.8 to 151.1)	2,460,127 (1,538,458 to 3,764,865)
Malawi	287 (129 to 520)	9.0 (4.0 to 16.3)	274.8 (133.0 to 516.6)	887,485 (429,675 to 1,668,567)	717 (395 to 1,154)	4.0 (2.2 to 6.4)	101.2 (62.2 to 156.3)	1,819,727 (1,118,633 to 2,809,615)
Mozambique	275 (138 to 501)	5.5 (2.8 to 10.1)	150.0 (85.9 to 251.4)	753,428 (431,332 to 1,262,312)	572 (301 to 965)	2.0 (1.0 to 3.4)	59.8 (39.8 to 86.3)	1,726,101 (1,148,258 to 2,489,165)
Rwanda	124 (58 to 228)	6.6 (3.1 to 12.2)	222.9 (117.7 to 382.8)	413,630 (218,315 to 710,276)	306 (156 to 526)	2.5 (1.3 to 4.4)	86.8 (57.5 to 126.3)	1,046,899 (692,995 to 1,523,577)
Somalia	149 (62 to 338)	11.3 (4.7 to 25.6)	297.1 (156.6 to 514.3)	351,360 (185,199 to 608,162)	434 (208 to 849)	4.2 (2.0 to 8.2)	97.9 (62.3 to 146.3)	1,003,487 (638,518 to 1,500,272)
South Sudan	257 (99 to 579)	9.0 (3.5 to 20.2)	335.5 (158.9 to 636.2)	1,000,097 (473,814 to 1,896,508)	670 (354 to 1,245)	4.9 (2.6 to 9.2)	149.8 (93.5 to 232.2)	2,052,763 (1,281,894 to 3,182,392)
Tanzania	624 (302 to 1,108)	6.9 (3.4 to 12.3)	224.9 (119.3 to 395.6)	2,111,746 (1,120,151 to 3,715,332)	2,431 (1,304 to 3,974)	4.5 (2.4 to 7.3)	100.6 (66.5 to 145.2)	5,523,606 (3,649,920 to 7,968,717)
Uganda	529 (249 to 945)	7.0 (3.3 to 12.6)	287.3 (136.3 to 550.3)	2,168,115 (1,028,891 to 4,153,536)	1,067 (577 to 1,726)	2.6 (1.4 to 4.3)	108.9 (66.5 to 170.1)	4,400,547 (2,686,746 to 6,874,082)
Zambia	226 (101 to 411)	7.9 (3.6 to 14.5)	260.0 (130.0 to 463.7)	733,988 (366,905 to 1,308,900)	602 (327 to 991)	3.6 (2.0 to 6.0)	98.9 (62.2 to 150.5)	1,644,164 (1,034,562 to 2,502,627)
Central Sub-Saharan Africa	351 (46 to 811)	1.7 (0.2 to 3.9)	64.5 (24.0 to 126.6)	1,370,060 (508,515 to 2,688,078)	562 (91 to 1,239)	0.5 (0.1 to 1.1)	19.4 (8.4 to 34.2)	2,299,091 (996,979 to 4,043,864)
Angola	87 (10 to 217)	1.8 (0.2 to 4.4)	62.7 (22.3 to 131.3)	313,855 (111,300 to 656,909)	125 (20 to 293)	0.5 (0.1 to 1.1)	20.1 (8.5 to 36.4)	522,340 (221,483 to 946,165)
Central African Republic	33 (5 to 79)	4.5 (0.7 to 10.6)	82.2 (29.9 to 159.9)	64,997 (23,633 to 126,460)	54 (10 to 123)	1.1 (0.2 to 2.4)	22.5 (9.9 to 38.7)	113,969 (50,363 to 196,275)
Congo	8 (1 to 19)	1.1 (0.2 to 2.6)	63.7 (23.6 to 125.6)	47,493 (17,591 to 93,593)	20 (3 to 47)	0.4 (0.1 to 1.0)	18.0 (8.0 to 31.6)	84,879 (37,550 to 149,006)

	Under-5				All ages			
Location	Deaths	Deaths per 100,000	Incidence per 1000	Cases	Deaths2	Deaths per 100,0003	Incidence per 10004	Cases5
Democratic Republic of the Congo	221<br>(28 to 548)	1.6<br>(0.2 to 3.9)	64.4<br>(24.1 to 126.1)	923,717<br>(345,123 to 1,809,600)	359<br>(58 to 820)	0.5<br>(0.1 to 1.0)	19.3<br>(8.4 to 33.7)	1,537,336<br>(672,731 to 2,687,683)
Equatorial Guinea	0<br>(0 to 0)	0.1<br>(0.0 to 0.4)	63.1<br>(21.9 to 127.4)	6,191<br>(2,147 to 12,500)	1<br>(0 to 2)	0.1<br>(0.0 to 0.2)	16.3<br>(7.2 to 29.0)	13,669<br>(6,014 to 24,221)
Gabon	1<br>(0 to 3)	0.5<br>(0.1 to 1.2)	55.5<br>(20.0 to 109.0)	13,791<br>(4,968 to 27,071)	4<br>(1 to 9)	0.2<br>(0.0 to 0.5)	15.2<br>(6.8 to 26.3)	26,839<br>(11,969 to 46,485)

References

1 GBD 2016 Causes of Death Collaborators. Global, regional, and national age-sex specific mortality for 264 causes of death, 1980-2016: a systematic analysis for the Global Burden of Disease Study 2016. *Lancet Lond Engl* 2017; **390**: 1151–210.

2 GBD 2016 Disease and Injury Incidence and Prevalence Collaborators. Global, regional, and national incidence, prevalence, and years lived with disability for 328 diseases and injuries for 195 countries, 1990-2016: a systematic analysis for the Global Burden of Disease Study 2016. *Lancet Lond Engl* 2017; **390**: 1211–59.

3 GBD 2016 DALYs and HALE Collaborators. Global, regional, and national disability-adjusted life-years (DALYs) for 333 diseases and injuries and healthy life expectancy (HALE) for 195 countries and territories, 1990-2016: a systematic analysis for the Global Burden of Disease Study 2016. *Lancet Lond Engl* 2017; **390**: 1260–344.

4 GBD 2016 Risk Factors Collaborators. Global, regional, and national comparative risk assessment of 84 behavioural, environmental and occupational, and metabolic risks or clusters of risks, 1990-2016: a systematic analysis for the Global Burden of Disease Study 2016. *Lancet Lond Engl* 2017; **390**: 1345–422.

5 GBD Diarrhoeal Diseases Collaborators. Estimates of global, regional, and national morbidity, mortality, and aetiologies of diarrhoeal diseases: a systematic analysis for the Global Burden of Disease Study 2015. *Lancet Infect Dis* 2017; **17**: 909–48.

6 GBD 2016 Diarrhoeal Disease Collaborators. Estimates of the global, regional, and national morbidity, mortality, and aetiologies of diarrhoea in 195 countries: a systematic analysis for the Global Burden of Disease Study 2016. *Lancet Infect Dis* 2018.

7 Merson MH, Rowe B, Black RE, Huq I, Gross RJ, Eusof A. Use of antisera for identification of enterotoxigenic Escherichia coli. *Lancet Lond Engl* 1980; **2**: 222–4.

8 Galbadage T, Jiang Z-D, DuPont HL. Improvement in detection of enterotoxigenic Escherichia coli in patients with travelers' diarrhea by increasing the number of E. coli colonies tested. *Am J Trop Med Hyg* 2009; **80**: 20–3.
